# Exceptions to the rule: When does resistance evolution not undermine antibiotic therapy in human bacterial infections?

**DOI:** 10.1093/evlett/qrae005

**Published:** 2024-03-02

**Authors:** Amrita Bhattacharya, Anton Aluquin, David A Kennedy

**Affiliations:** Department of Biology, The Pennsylvania State University, University Park, PA, United States; Department of Biology & Schreyer Honors College, The Pennsylvania State University, University Park, PA, United States; Department of Biology, The Pennsylvania State University, University Park, PA, United States

**Keywords:** antibiotic resistance, drug resistance, antibiotic stewardship, nosocomial infection, indirect transmission, literature review

## Abstract

The use of antibiotics to treat bacterial infections often imposes strong selection for antibiotic resistance. However, the prevalence of antibiotic resistance varies greatly across different combinations of pathogens and drugs. What underlies this variation? Systematic reviews, meta-analyses, and literature surveys capable of integrating data across many studies have tried to answer this question, but the vast majority of these studies have focused only on cases where resistance is common or problematic. Yet much could presumably be learned from the cases where resistance is infrequent or absent. Here we conducted a literature survey and a systematic review to study the evolution of antibiotic resistance across a wide range of pathogen-by-drug combinations (57 pathogens and 53 antibiotics from 15 drug classes). Using Akaike information criterion-based model selection and model-averaged parameter estimation we explored 14 different factors posited to be associated with resistance evolution. We find that the most robust predictors of high resistance are nosocomial transmission (i.e., hospital-acquired pathogens) and indirect transmission (e.g., vector-, water-, air-, or vehicle-borne pathogens). While the former was to be expected based on prior studies, the positive correlation between high resistance frequencies and indirect transmission is, to our knowledge, a novel insight. The most robust predictor of low resistance is zoonosis from wild animal reservoirs. We also found partial support that resistance was associated with pathogen type, horizontal gene transfer, commensalism, and human-to-human transmission. We did not find support for correlations between resistance and environmental reservoirs, mechanisms of drug action, and global drug use. This work explores the relative explanatory power of various pathogen and drug factors on resistance evolution, which is necessary to identify priority targets of stewardship efforts to slow the spread of drug-resistant pathogens.

## Introduction

Antibiotics impose strong selection on pathogens, often leading to the evolution of antibiotic resistance (Davies & Davies, 2010; zur Wiesch et al., 2011). However, the evolution of resistance is variable among different pathogens and treatments (Kennedy & Read, 2017, 2020). For example, the majority of *Staphylococci* isolates in British hospitals were resistant to penicillin within 6 years of its introduction (Barber & Whitehead, 1949). In contrast, syphilis has been successfully treated with penicillin since 1944 (Tien et al., 2020). Likewise, antibiotic treatments for Lyme disease, brucellosis, and chlamydia have remained effective despite long histories of drug treatment (Tien et al., 2020; Trott et al., 2018). Nonetheless, previous studies on the evolution of drug resistance have largely focused on pathogens where drug resistance is widespread (Bell et al., 2014; Collignon et al., 2018). For example, efforts to document patterns of drug resistance at national or global scales have typically focused only on the pathogens where resistance evolution poses the greatest threat to public health (CDC., 2019a, Garner et al., 2015; Tacconelli et al., 2018). Less is known about cases where drug resistance is not yet a threat to treatment efficacy. Understanding this variation in outcomes could lead to new evolutionary insight and novel strategies or compounds that increase the evolutionary robustness of infectious disease treatments. Here we explore the factors that best explain this variation in antibiotic resistance across “pathogen × drug” combinations.

**Figure 1.**
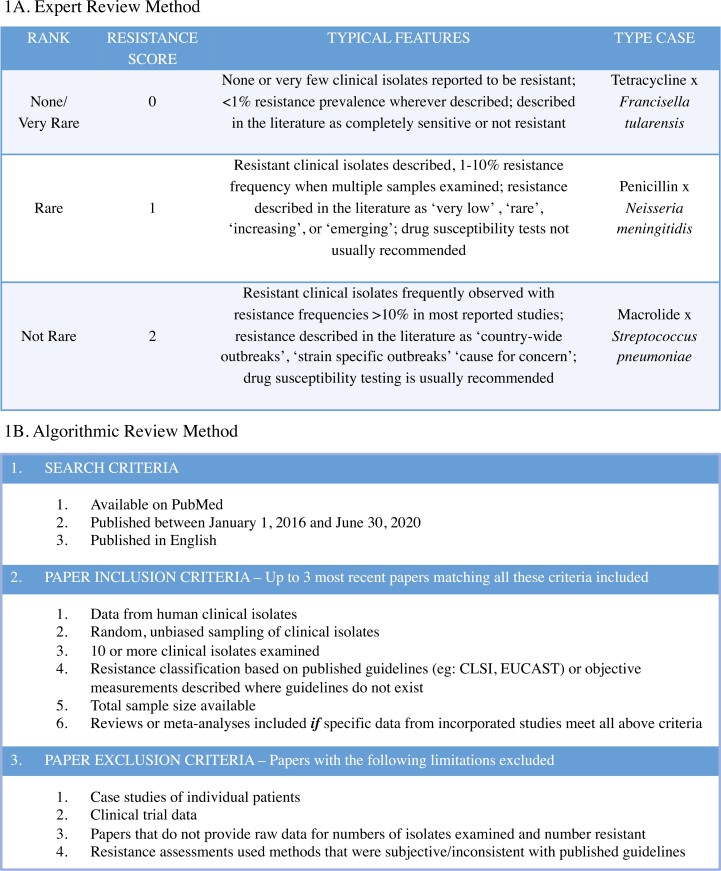
The data collection schemes used for the (A) expert review method (ERM) and (B) algorithmic review method (ARM).

We examined 182 unique “pathogen × drug class” combinations, including 57 pathogens and 53 antibiotics within 15 drug classes, derived from a modern medical microbiology textbook (Cornelissen & Hobbs, 2020). Note that not every drug class is used to treat every pathogen (in part due to inherent differences in the efficacy of various drugs against various pathogens, often termed “intrinsic resistance” which is distinct from the acquired resistance that we focus on here), resulting in fewer “pathogen × drug class” combinations than the product of pathogen and drug class numbers. We documented resistance frequencies using two complementary approaches that we term the “expert review method” (ERM) and “algorithmic review method” (ARM) (Figure 1, Supplementary Tables S1 and S2, see also *Methods*). Here and throughout the manuscript we use the term “resistance frequencies” to indicate the qualitative or quantitative prevalence of resistance. Briefly, the data for the ERM approach were collected via consensus between at least two authors (A.B. and A.A.) who independently conducted literature surveys to qualitatively classify resistance into one of three categories (Figure 1A) with corresponding numeric scores: Very rare/None (0), Rare (1), or Not rare (2). The ERM literature surveys utilized information from peer-reviewed scientific papers and included papers with data about resistance prevalence, clinical case studies, or descriptions of resistance patterns in peer-reviewed articles. In contrast, the data for the ARM approach were collected from prevalence data (number of isolates resistant out of total number studied) using peer-reviewed scientific papers that met a strict set of criteria (Figure 1B). The ARM search was expanded to include specific antibiotics within every drug class resulting in 376 “pathogen × antibiotic” combinations. While the ARM data are more precise and less subject to investigator bias than the ERM data, data that met the inclusion criteria were unavailable for 8 pathogens. On the other hand, by incorporating multiple types of data, such as information available from clinical case studies and the descriptions of resistance patterns in review articles (Figure 1A), the ERM approach provided information for all 182 “pathogen × drug class” combinations including those for which no papers met the strict selection criteria of the ARM approach (Figure 1).

We examined 14 different factors believed to be associated with resistance evolution (Beaber et al., 2004; Breijyeh et al., 2020; Chamchod & Ruan, 2012; Exner et al., 2017; Fantin et al., 2009; Holmes et al., 2016; Kristiansson et al., 2011; Laxminarayan et al., 2013; Llor & Bjerrum, 2014; Morley et al., 2019; Parsley et al., 2010; Schwarz et al., 2001; Stratton, 2003; Wellington et al., 2013; Zhang et al., 2013) to identify factors that best explain the variation in resistance (Table 1). Pathogen traits examined include whether the pathogen has documented cases of transmission in hospital settings (nosocomial), and if the pathogen has animal (zoonosis), human microbiome (commensal), or environmental reservoirs. We also examined “pathogen type” as defined in the medical microbiology textbook (Cornelissen & Hobbs, 2020) (Gram positive, Gram negative, anaerobic, or other), “transmission type” as defined by the CDC (CDC, 2012) (direct, meaning transfer from a reservoir to a new host via direct, physical contact or close proximity, or indirect, meaning transfer from a reservoir to a new host via suspended air particles, animate intermediaries, or inanimate objects), and whether human-to-human transmission has been documented (human transmission). Two modes of horizontal gene transfer (natural competence and conjugation) were examined. Pathogens were noted to have natural competence or conjugation if available evidence suggested a pathogen had the requisite genetic machinery. Drug factors examined in our analyses included the amount of time a drug has been in use (drug date), the estimated use of each drug measured in daily defined doses (global drug use), and the mechanism of drug action (bactericidal or bacteriostatic). Differences in research effort devoted to different pathogens and drugs were accounted for by measuring the total number of publications for each pathogen, drug class, or antibiotic on the database PubMed (research effort pathogen/drug). The distribution of each factor across the “pathogen × drug” combinations is provided in Supplementary Tables S1 and S2. To the best of our knowledge, this work is the first systematic investigation of resistance to include such a wide range of human bacterial pathogens and predictor variables.

**Table 1.** Factor definitions.

Factor	Levels	Definition
Nosocomial	Yes/ No	Bacteria that are thought to often be acquired from the hospital environment after admission
Zoonosis	Yes/No	Bacteria that replicate within animal reservoirs in addition to their infected human hosts. Sustained human to human transmission is independent of this classification
Naturally competent	Yes/No	Bacteria contain genetic machinery for horizontal gene transfer via uptake of DNA from the external environment
Conjugation	Yes/No	Bacteria contain genetic machinery for horizontal gene transfer between bacterial cells via direct cell-to-cell contact
Commensal	Yes/No	Bacteria are opportunistic pathogens that can be found within the microbiome of healthy human hosts
Transmission type	Direct/Indirect	Mode of transmission. Direct transmission describes bacteria that transfer from an infection source to a new host via direct, physical contact or close proximity (e.g., wound infections caused by contact with contaminated soil, sexually transmitted infections, or large droplets). Indirect transmission describes bacteria that transfer from an infection source to a new host via suspended air particles (airborne), animate intermediaries (vectors), or inanimate objects (vehicles such as canned food, contaminated water)
Human transmission	Yes/No	Bacteria where transmission has been documented to occur from one human host to another whether through direct or indirect modes
Pathogen type	Gram positive, Gram negative, anaerobic, other	Bacterial classification based on Gram staining or respiration mode as typically discussed in medical microbiology
Research effort pathogen	Continuous	Research effort as described by the total number of publications returned for each bacterial pathogen name when searching on the database PubMed
Research effort drug/antibiotic	Continuous	Research effort as described by the total number of publications returned for each drug class, or antibiotic name when searching on the database PubMed
Drug mechanism	Bactericidal/Bacteriostatic	Broad mechanism of drug action. Bactericidal drugs act by killing pathogens and bacteriostatic drugs inhibit the growth of pathogens without killing them
Drug/antibiotic date	Continuous	The year a drug class or antibiotic was first discovered defined by the date of the earliest reference found on PubMed
Global drug use	Continuous	Drug use as described by the average consumption of drugs across China, India, and the USA (the three countries with highest total drug use). Data are from 2015, the most recent year for which data were publicly available. Values are measured in defined daily doses.
Environmental reservoir	Yes/No	Bacteria that replicate in environmental reservoirs outside the human host. Sustained human to human transmission is independent of this classification.

## Results

### Antibiotic resistance varies widely within and between pathogens and drugs

**Figure 2.**
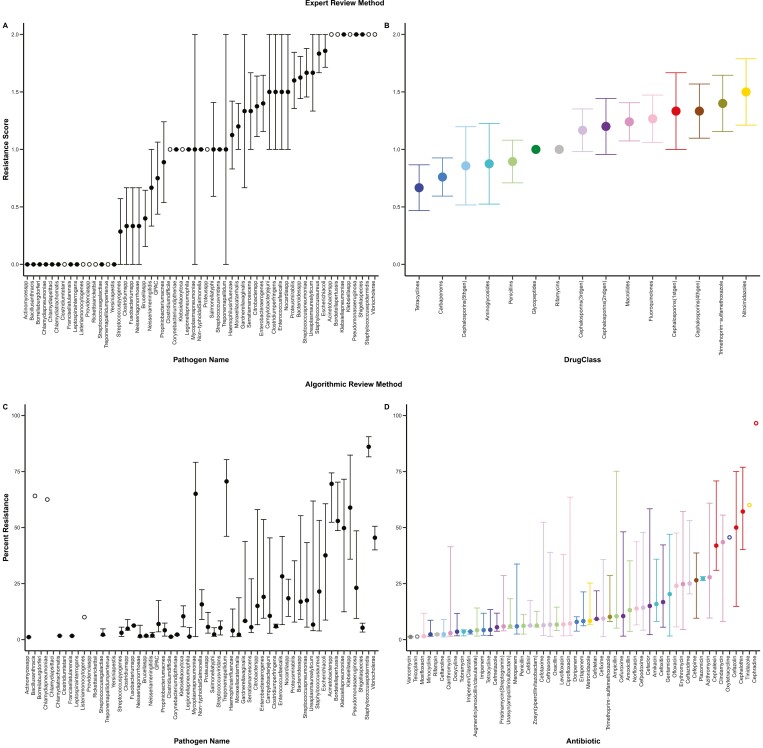
Drug resistance levels vary across pathogens and drugs. Panels A and B show data from the expert review method (ERM) and panels C and D show data from the algorithmic review method (ARM). Note that the order of pathogens in panels A and C are the same, and that the colors of the points in panels B and D match for antibiotics that belong to the corresponding drug class. Filled circles in panels A and B depict the mean resistance score and error bars show ± 1 standard error across, respectively, pathogens and drug classes in the ERM dataset. Resistance scores 0, 1, and 2 represent the categories Very rare/None, Rare, and Not rare as described in Figure 1A. Open circles depict resistance scores based on a single pathogen by drug class combination. Filled circles in panels C and D show the median resistance percentage with error bars depicting the 25th and 75th quantiles for the pathogens and antibiotics in the ARM dataset, respectively. Open circles depict resistance scores based on a single data point. Note that no ARM data were available for 8 pathogens (missing circles in panel C). These missing data points are predominantly pathogens that had low resistance in the ERM dataset.

Using our two approaches (ARM and ERM) we characterized the prevalence of resistance across 57 pathogens and 15 drug classes (Figure 1, Supplementary Tables S3 and S4). For the ERM approach, resistance was scored 0, 1, or 2 by author consensus (see *Methods* for details) with 0 meaning that resistance was extremely rare, and 2 meaning that resistance was common in at least some geographic locations (Figure 1A, Supplementary Table S3). Overall, the ERM dataset was comprised of 182 data points, with each data point being the score for a unique pathogen-by-drug-class combination. In that dataset, the overall mean resistance score of “pathogen × drug class” combinations was 1.05 (median: 1.0) with a standard deviation of 0.84 (Figure 2A and B). For the ARM approach, the prevalence of resistance was scored for 376 unique “pathogen × antibiotic” combinations (149 unique “pathogen × drug class” combinations) using the counts of resistant and not resistant isolates from three peer-reviewed scientific papers for each combination (or up to three papers if fewer than three were available), resulting in 825 unique data points (Figure 1B, see *Methods* for more details including inclusion/exclusion criteria). These data included 425,767 individual tests for resistance, collected from 352 unique scientific papers (Supplementary Table S4). For the ARM dataset, the overall mean percentage of resistant isolates was 24.29% (median: 9.02%) with a standard deviation of 28.69% (Figure 2, panels C and D). We thus observed that resistance varied widely between pathogens and drugs across both approaches (Figure 2). There is a positive correlation in the resistance score of “pathogen × drug class” combinations between the ERM and ARM data (Pearson’s product moment correlation, *r* = 0.61, *t* = 9.19, *df* = 146, *p* < .001), which can be partially visualized by comparing pathogen resistance scores in Figure 2, panels A and C.

#### Characteristics of pathogens and drugs varied across all putative explanatory variables

**Figure 3.**
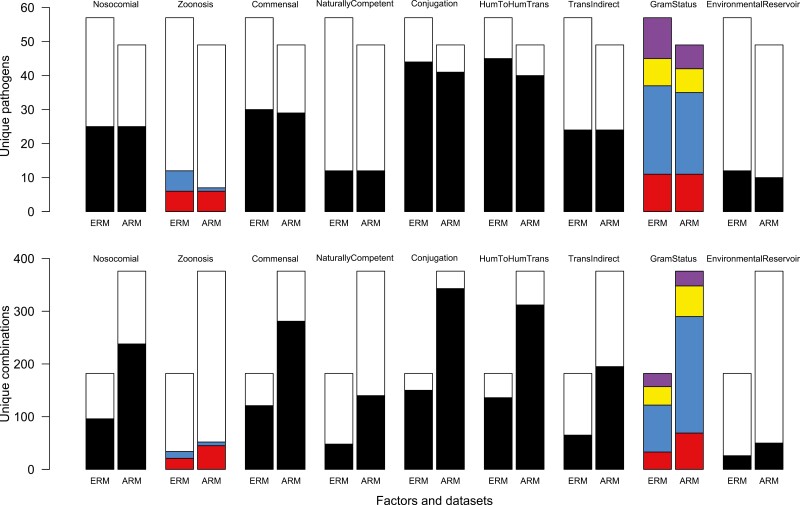
Distribution of pathogen factors across both datasets. (Top row) Bar plots indicate the number of unique bacterial pathogens in the expert review method (ERM) and algorithmic review method (ARM) datasets that had (black color) or lacked (white color) the feature labeled above each bar. For “Zoonosis,” red indicates bacteria with domesticated animal reservoirs, blue indicates bacteria with wild animal reservoirs, and white indicates non-zoonotic bacteria. For “GramStatus,” red indicates Gram-positive bacteria, blue indicates Gram-negative bacteria, yellow indicates anaerobic bacteria, and purple indicates bacteria classified as other. (Bottom row) Bar plots indicate the total number of unique bacteria × drug class (ERM) or bacteria × antibiotic (ARM) combinations in the respective datasets. Colors represent the same categories as above.

Data for 14 different pathogen and drug characteristics (Table 1) were collected by author consensus for each of the 57 pathogens and 53 antibiotics (see *Methods*, Supplementary Table S5). The characteristics of the pathogens and drugs included in our respective ERM and ARM datasets are shown in Figure 3 with the full data listed in Supplementary Tables S1 and S2. Note that the ARM dataset included only 49 of the original 57 pathogens because the other 8 pathogens had no data that fit the inclusion criteria for our ARM approach. The distribution of pathogen factors nevertheless remained similar between the two datasets (Figure 3) with the notable exception of zoonotic pathogens, where only 1 remaining pathogen in the ARM dataset had a wild animal reservoir. Notably, many of the drug and pathogen factors had significant correlations among them (Supplementary Figure S1) creating opportunities for false positive and false negative trends when each factor was analyzed independently. We, therefore, supplemented single-factor analyses with multi-factor analysis, model selection, Akaike information criterion (AIC)-based metrics (see *Methods* for details), and model-averaged parameter estimates to interrogate the explanatory importance of each factor.

#### Nosocomial transmission and indirect transmission associate with high frequencies of drug resistance in both datasets

We performed single-factor regression for each set of factors (linear models with normally distributed error for ERM, and logistic models for ARM, see *Methods* for details) to determine which factors significantly correlated with resistance frequencies. Both datasets were also analyzed using a suite of mixed-effects models (again, linear models with normally distributed error for ERM, and logistic models for ARM, see *Methods* for details) including all possible combinations of 14 explanatory variables resulting in 16,384 candidate models (see *Methods* for details). These models were compared using ΔAIC scores, which is the difference in AIC between our best-fitting model (lowest AIC model) and any focal model. We present ΔAIC for all models that differ from our best model by exactly one factor (either added or removed), keeping in mind that ΔAIC scores less than 2 indicate a similar level of support as the best-fitting model. Factors included in the best-fitting model that have the highest explanatory power produce high ΔAIC scores when removed (i.e., ΔAIC > 2). Among factors not included in the best model, those with reasonable support produce smaller ΔAIC scores when added (i.e., ΔAIC << 2). The AIC weight of a factor is the probability that the factor is part of the true process used to generate the data given one of the candidate models is correct (see *Methods* for details on how to calculate it). AIC weight scores range from 0 to 1, with 0 indicating that the factor is not part of the true underlying model and 1 implying absolute confidence of the factor’s inclusion. When a factor has an AIC weight greater than 0.5 it thus means that factor is more likely than not to be included in the true process underlying the generation of the data. To test the robustness of our AIC weights, we also conducted a “leave-one-factor-out” analysis, in which we iteratively removed a single factor from our model and recalculated the AIC weights for the remaining models (see *Methods* for more details). Except where noted, this analysis had no impact on our conclusions (Supplementary Figure S2). Lastly, we generated model-averaged parameter estimates to identify the direction and significance of the association between factors and resistance frequencies. Significance is indicated by 95% confidence intervals that do not overlap 0 for the ERM data and that do not overlap 1 for the ARM data (the ARM estimates are presented as odds ratios (OR)). Higher values than these thresholds represent positive correlations with resistance frequencies.

**Figure 4.**
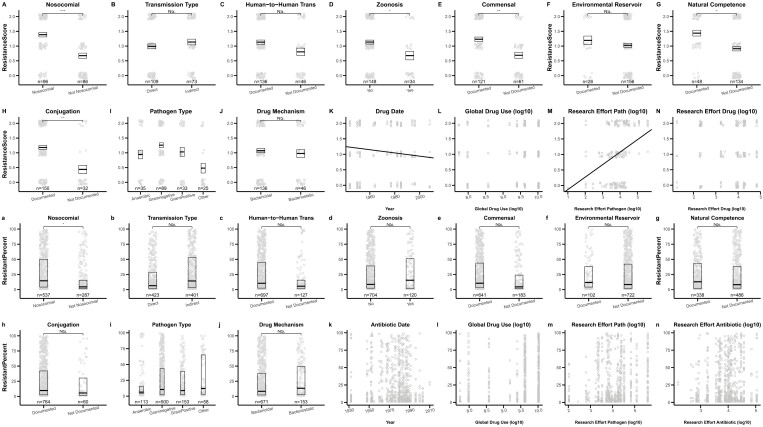
Single-factor analyses of drug resistance evolution in the ERM (top two rows) and ARM (bottom two rows) datasets. Each panel depicts the correlation between resistance and the factors described in Table 1. Grey open circles are the raw data points. Black box plots show the mean ± 1 standard error (top two rows) or the median and first and third quartiles (bottom two rows). Significance levels are represented as NS (*p* > .05), * (*p* < .05), ** (*p* < .01), and *** (*p* < .001). The presence of a black regression line indicates a significant association for continuous factors. For categorical factors and data, points are, respectively, jittered along the x- or y-axis to aid visualization.

**Figure 5.**
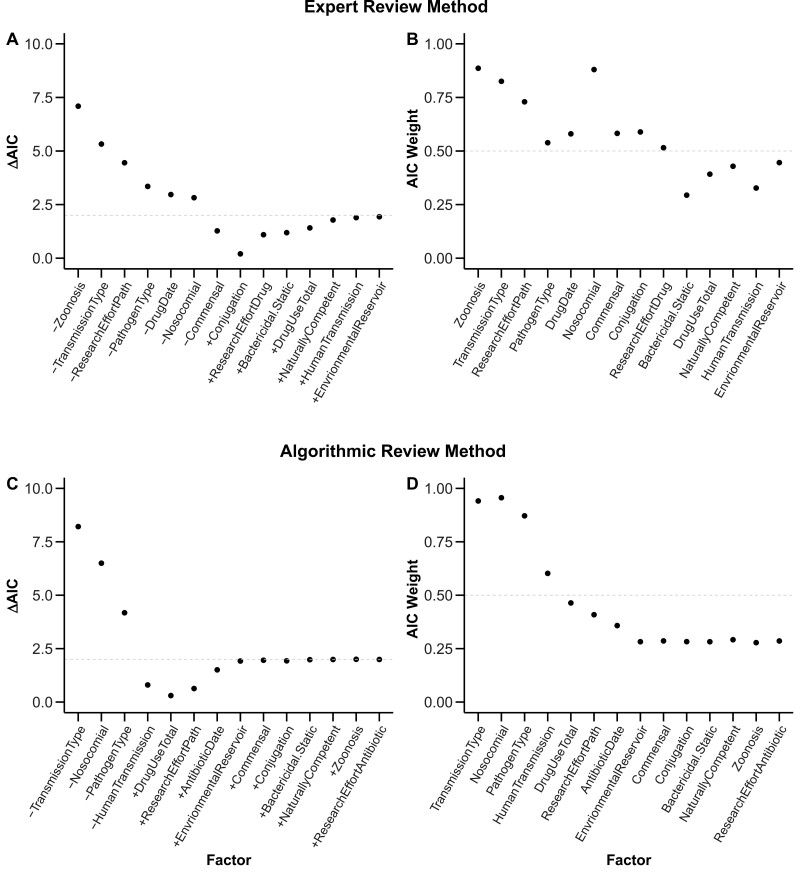
Relative order of importance of factors in explaining variation in drug resistance levels. Panels A and B represent the ΔAIC scores and AIC weights, respectively, of factors for the expert review method (ERM) dataset. Panels C and D represent the ΔAIC scores and AIC weights of factors, respectively, for the ARM dataset. ΔAIC scores represent the difference in AIC scores between the best-fitting model with the lowest AIC score and a model with one factor added or removed (denoted, respectively, by + and  − signs on x-axis labels). Horizontal dashed lines in panels A and C denote ΔAIC = 2. AIC weights of a factor represent the cumulative weights of all models that include the factor. All factors above the dashed line in panels B and D have AIC weight values greater than 0.5. Factors in panels A and B are arranged in decreasing order of support based on their ΔAIC score in panel A. Factors in panels C and D are arranged in decreasing order of support based on their ΔAIC score in panel C.

**Figure 6.**
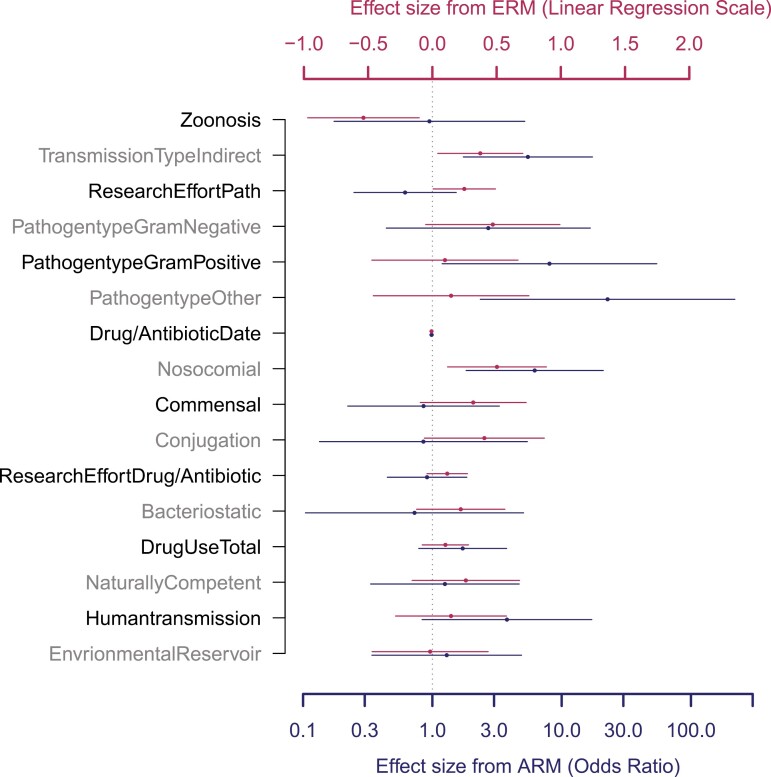
Model-averaged parameter estimates of all factors across both datasets. Model-averaged parameter estimates for each factor were calculated across all models that included the factor. Results for the expert review method (ERM) are represented on the linear regression scale and shown in red. Results from the algorithmic review method (ARM) are depicted as odds ratios (OR) calculated from the model-averaged logistic regression parameter estimates. Points show effect sizes with error bars depicting 95% confidence intervals. Note that for factors with continuous variables, estimated effect sizes are unscaled. Estimates are determined to be significant if the error bars do not overlap 0 for ERM or 1 for ARM (dashed line).

Our single-factor analyses showed a positive correlation between the frequency of resistance and the factor “nosocomial” in both the ERM (χ^2^ = 17.28, *df* = 1, *p* < .001, Table 2) and ARM datasets (χ^2^ = 6.52, *df* = 1, *p* = .010, Figure 4, Table 2), and a nonsignificant trend toward a positive correlation between resistance frequency and indirect transmission in the ARM dataset (χ^2^ = 2.66, *df* = 1, *p* = .102, Table 2). In general agreement with above, using our suite of mixed-effects models, the factors “nosocomial” and “transmission type” (direct or indirect) emerged with the highest explanatory power across both datasets according to our AIC-based metrics. For both datasets, the factors “nosocomial” and “transmission type” were included in the best model and had high ΔAIC scores when removed from that best model, further indicating their importance (Figure 5, panels A and C; “nosocomial”: 2.82 (ERM) and 6.49 (ARM); “transmission type”: 5.32 (ERM) and 8.21 (ARM)). This was also confirmed by model weights where “nosocomial” had AIC weights of 0.88 (ERM) and 0.96 (ARM) while “transmission type” had AIC weights of 0.82 (ERM) and 0.94 (ARM) indicating strong support for both factors in both datasets. Lastly, model-averaged parameter estimates (Figure 6) further confirmed that nosocomial transmission is significantly associated with increased frequencies of drug resistance in both datasets (ERM estimate: 0.50 [0.12, 0.89]; ARM estimate (OR): 6.18 [1.83, 20.95]), as is indirect transmission (ERM estimate: 0.37 [0.04, 0.70]; ARM estimate (OR): 5.48 [1.74, 17.30]).

**Table 2.** Estimated effect sizes and 95% confidence intervals from the single-factor analysis for each model factor. Reported confidence intervals were derived by adding or subtracting two standard errors from the point estimate. Bolding indicates that the confidence interval does not overlap zero for the ERM dataset or one for the ARM dataset. Note that likelihood ratio tests were used to determine factor significance in Figure 4.

Factor	ERM (Natural scale)	ARM (OR)
Nosocomial	**0.80** [**0.46, 1.13**]	**4.63** [**1.46, 14.71**]
Zoonosis	**−0.57** [**−1.02, −0.12**]	0.86 [0.15, 5.03]
Commensal	**0.60** [**0.24, 0.97**]	1.42 [0.40, 5.00]
NaturallyCompetent	**0.60** [**0.17, 1.03**]	1.40 [0.34, 5.82]
Conjugation	**0.88** [**0.45, 1.30**]	1.79 [0.34, 9.50]
HumanTransmission	0.15 [−0.08, 0.84]	3.76 [0.80, 17.65]
TransmissionType.Indirect	0.24 [−0.14, 0.63]	2.71 [0.81, 9.07]
PathogenType.GramNegative	0.37 [−0.17, 0.91]	3.31 [0.53, 20.55]
PathogenType.GramPositive	0.37 [−0.17, 0.84]	**8.03** [**1.03, 62.33**]
PathogenType.Other	−0.47 [−1.09, 0.16]	4.79 [0.50, 45.76]
Environmental	0.22 [−0.27, 0.71]	1.59 [0.34, 7.50]
Bacterostatic	0.16 [−0.18, 0.51]	0.83 [0.11, 6.16]
AntibioticDate	−0.01 [−0.01, 0.00]	**0.99** [**0.98, 1.00**]
DrugUseTotal	0.05 [−0.13, 0.23]	1.62 [0.70, 3.75]
ResearchEffortDrug	0.09 [−0.06, 0.23]	0.99 [0.54, 1.81]
ResearchEffortPathogen	**0.40** [**0.15, 0.65**]	1.27 [0.47, 3.42]

#### Zoonosis associates with low drug resistance in the ERM dataset

“Zoonosis” had the highest ΔAIC score (7.09) in the ERM dataset indicating high explanatory power for this factor (Figure 5A). The AIC weight of “zoonosis” for the ERM dataset was 0.89 with its model-averaged parameter estimate (−0.54 [−0.97, −0.27]) similarly indicating an association between zoonosis and low frequencies of antibiotic resistance (Figure 6). After observing this trend, we divided zoonotic pathogens into those with wild animal reservoirs and those with domestic reservoirs, which revealed that human bacterial pathogens with wild animal reservoirs have a substantially lower frequency of resistance than non-zoonotic pathogens and zoonotic pathogens with domesticated animal reservoirs (Supplementary Figure S3). In the ARM analysis, we did not find support for an association between “zoonosis” and resistance (ΔAIC score: 1.99, AIC weight: 0.27, OR: 0.95 [0.17, 5.18]), but this may be due to a lack of power because the ARM dataset included only 1 pathogen with a wild animal reservoir (Figure 3 and Supplementary Table S4). These results were also backed up by our single-factor analysis which found a negative association with resistance frequencies for “zoonosis” (χ^2^ = 4.95, *df* = 1, *p* = .026, Table 2) in the ERM dataset, but no significant correlation in the ARM dataset (χ^2^ = 0.03, *df* = 1, *p* = .869).

#### Gram positivity and research effort of pathogens associate with high frequency of resistance in the ARM and ERM analyses, respectively

The factor “pathogen type” which documented Gram status and aerobicity of pathogens (Supplementary Table S5) was included in the best-fitting models and had AIC weight scores of 0.54 and 0.87 in the ERM and ARM datasets, respectively (Figure 5, see *Methods* for details). However, the associations with resistance were statistically significant only in the ARM dataset with Gram-positive (OR = 8.05 [1.19, 54.38]) and Gram-unclassified (“other”) pathogens (OR = 22.65 [2.35, 218.27]) being associated with higher frequencies of resistance than anaerobic pathogens (Figure 6). Similar results were seen in the single-factor analysis where we found a significant effect of “pathogen type” in the ERM dataset (χ^2^ = 8.39, *df* = 3, *p* = .039, Table 2). The effect size of Gram positivity was only significant in the ARM dataset (Table 2). The factor “research effort pathogens” (defined in Table 1) was included in the best-fitting model of the ERM dataset with an AIC weight score of 0.72, and a model-averaged effect size of 0.25 [0.01, 0.49] (Figure 6) indicating a positive association between research effort and resistance in this dataset. This was also supported by a positive association in the single-factor analysis of the ERM dataset (χ^2^ = 7.25, *df* = 1, *p* = .007, Table 2).

#### Natural competence, conjugation, commensals, drug discovery date, and human-to-human transmission show mixed evidence of association with resistance

The factors “commensal” and “drug date” were included in the best model in the ERM dataset with respective ΔAIC scores of 1.27 and 2.97 and respective AIC weights of 0.58 and 0.58 (Figure 5). They were also supported by single-factor analyses of the ERM dataset (commensal χ^2^ = 8.25, *df* = 1, *p* = .003; drug date χ^2^ = 7.29, *df* = 1, *p* = .007, Table 2). The factor “human-to-human transmission” was included in the best model in the ARM dataset only with ΔAIC score 0.79 and had AIC weight 0.60 (Figure 4). It also had a suggestive association in the single-factor analysis of the ARM dataset (χ^2^ = 2.83, *df* = 1, *p* = .092, Table 2). However, all three factors had non-significant model-averaged effect sizes (Figure 6).

The factors “natural competence” and “conjugation” which are modes of horizontal gene transfer, did not emerge as important variables in our AIC-based multi-factor analyses (Figure 5), although both showed positive correlations with resistance frequency in our single-factor analysis of the ERM dataset (natural competence χ^2^ = 6.09, *df* = 1, *p* = .013; conjugation χ^2^ = 13.94, *df* = 1, *p* < .001, Table 2). Notably, in our leave-one-factor-out analysis, when the factor “commensal” was eliminated, “natural competence” became highly important in both the ERM and ARM datasets (respective AIC weights of 0.96 and 0.92, Supplementary Figure S2). We thus cannot draw strong conclusions regarding the importance of natural competence. This lack of certainty is unavoidable given that 8 of our 12 naturally competent pathogens were also commensal (Supplementary Table S5).

#### Environmental reservoirs, total drug use, and mechanisms of drug actions do not show evidence of association with resistance

Some factors found no support from any of our analyses across either dataset in explaining variation in resistance across “pathogen × drug” combinations. These factors include “environmental reservoir,” “drug mechanism” (bactericidal/bacteriostatic), and “global drug use.” These factors did not show significance in our single-factor analyses, were not included in the best model, had AIC weights < 0.5, and had nonsignificant model-averaged parameter estimates (Figure 6). These factors also did not emerge as important predictors in any of the scenarios in our leave-one-factor-out analyses (Supplementary Figure S2), although “drug mechanism” did cross the 0.5 line in the ERM dataset when the factor drug date was removed.

## Discussion

**Figure 7.**
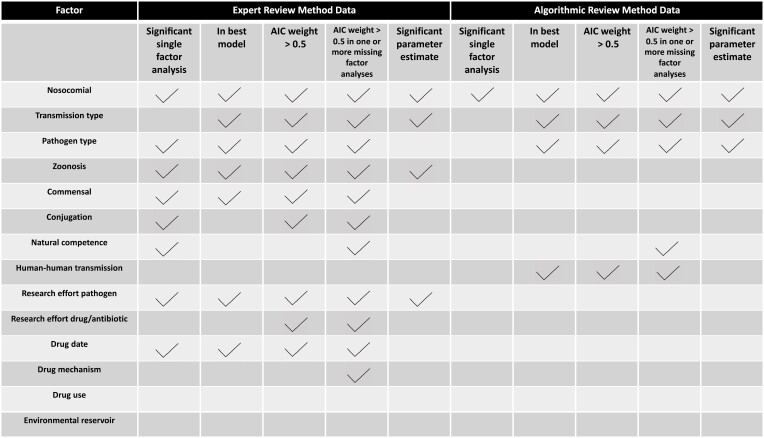
Summary of results.

Insights about drivers of antibiotic resistance have largely relied on studies focused on small sets of bacterial pathogens, or the bacterial pathogens where resistance poses the greatest threats to human health (CDC, 2019; Lin & Lan, 2014; Lutgring, 2019; Pachori et al., 2019; Paterson, 2006; Santajit & Indrawattana, 2016; Tacconelli et al., 2018). These approaches leave out information that can be gained by contrasting pathogens where resistance does and does not pose an urgent threat. Here, we bridge this knowledge gap by documenting the variation in antibiotic resistance across 57 human bacterial pathogens and 53 antibiotics across 15 drug classes. We explore associations between resistance frequencies and 14 factors theorized to be correlated with the evolution of resistance (Table 1). Our results show that the factors “nosocomial” and “indirect transmission” are associated with increased frequencies of resistance while the factor “zoonosis,” particularly with wild animal reservoirs, is associated with reduced frequencies. We also found partial support for the effects of pathogen type, horizontal gene transfer, commensalism, and human-to-human transmission, but this support was inconsistent across our datasets and analyses. We did not find support for environmental reservoirs, mechanisms of drug action, and global drug use in explaining the observed variation in resistance frequencies in either dataset. Notably, our study is observational and correlative, so it is not possible to state from this study alone which factors if any drive resistance evolution. Instead, our results should be used in conjunction with evolutionary theory to challenge existing hypotheses and generate novel ones. Results are summarized in Figure 7.

The lack of support for total drug use as an explanatory factor for frequencies of drug resistance seems curious given the evidence that drug use can drive resistance increases within individual hospitals or countries (Bell et al., 2014; Goossens et al., 2005; Shapiro et al., 2020). These studies, however, explored correlations between antibiotic use and resistance of specific drug-pathogen combinations across countries whereas our study is exploring these correlations across drug-pathogen combinations. Our lack of correlation may therefore be explainable by differences in the properties of the pathogens, the properties of the drugs, the fitness costs of resistance, or even the scale at which resistance was measured. We do note, however, that our results are consistent with the findings of a prior study (Collignon et al., 2018), which similarly found a lack of association between drug use and drug resistance at large geographic scales. In addition to the previously discussed possibilities, the lack of association we found might also be due to feedbacks between the frequency of drug resistance and the frequency with which particular drugs are prescribed, since drugs are presumably less likely to be employed when resistance to them is widespread.

Our results, across both datasets and all analyses, identify being nosocomial (hospital-acquired) as one of the strongest correlates of high resistance frequencies (Figure 7). The high prevalence of antibiotic resistance among nosocomial pathogens is often attributed to the interplay of high antibiotic usage in hospital environments, which may impose selection for resistance, and imperfect infection control practices, which may promote transmission of resistant pathogens in hospitals (Avershina et al., 2021; Shapiro et al., 2020; Struelens, 1998). Our results not only demonstrate that hospital environments are associated with higher resistance, which is well-appreciated (Levin & Udekwu, 2010; Struelens, 1998; Wang & Harnden, 2011), but highlight the relative importance of hospital transmission when compared against other factors (Figure 6 and Figure 7).

To the best of our knowledge, these results are the first to demonstrate a significant correlation between indirect transmission and high resistance frequencies (Figure 6), although the importance of pathogen transmission itself in resistance spread is well appreciated (Holmes et al., 2016; Laxminarayan et al., 2013). Our approach does not allow us to identify the mechanism underlying this correlation, but rather, we stress that our results can form the basis of future hypotheses. If the relationship is indeed causative, one speculative explanation for the observed pattern arises from the fact that pathogen transmission frequently declines when illness is observable (Kennedy, 2023), and that the decline in transmission is more pronounced for directly transmitted infections (Ewald, 1993; Gravina et al., 2020; Vigfusson et al., 2021). With the notable exception of bystander selection (Tedijanto et al., 2018), antibiotics are typically used to treat observed infections (Tedijanto et al., 2018). Therefore, if a resistant variant were to arise following the observation of infection and the commencement of drug treatment, it would be more likely to spread if it were indirectly transmitted than directly transmitted, and thus, all else equal, resistant variants would have higher fitness if they were indirectly transmitted than directly transmitted. If future research confirms this hypothesis, efforts to better understand the relationship between the mode of transmission and resistance may offer novel approaches toward mitigating the spread of resistance.

We find that zoonotic pathogens are significantly associated with lower frequencies of resistance in the ERM dataset (Figure 6). While previous studies have found the opposite (Cheng et al., 2019; Holmes et al., 2016; Silbergeld et al., 2017; Walusansa et al., 2019), those studies have focused on zoonotic pathogens of domesticated animals. We show that pathogens with wild animal reservoirs exhibit significantly lower frequencies of resistance than non-zoonotic pathogens, while pathogens with domesticated animal reservoirs exhibit frequencies of resistance comparable to non-zoonotic pathogens (Supplementary Figure S3). This pattern may arise due to limited antibiotic exposure within wild animal reservoirs thus limiting the strength of selection for resistance. This lack of selection for resistance would only be stronger for pathogens that have little to no transmission between human hosts which is often correlated with being zoonotic (Supplementary Figure S1, e.g., Lyme disease, anthrax, rickettsia, etc.). Notably, we only detect a relationship between “zoonosis” and low frequencies of resistance in the ERM dataset. One possible reason the same pattern is not observed in the ARM dataset is because this dataset only contains one pathogen with a wild animal reservoir (Figure 3).

We also find that differences in resistance are associated with Gram status (Figure 7, Figure 6). There has been a strong focus on drug resistance management for Gram-negative bacteria because there are few drugs to treat them (Breijyeh et al., 2020; Exner et al., 2017; Fitzpatrick, 2020). Our results, however, show that resistance frequencies are elevated in Gram-positive and Gram-unclassified pathogens relative to aerobic bacteria (Figure 6, Figure 7), highlighting the need to focus on sustainable approaches to treat these pathogens.

While we did not find strong support for conjugation as a predictor of resistance frequency, this result may be due in part to our classification criteria. We classified “conjugation” based on whether a pathogen is known to have the requisite genetic machinery or whether conjugation has been observed. However, it is likely that some pathogens are misclassified in that they undergo conjugation, but conjugation has not yet been documented. While this could in principle impact our conclusions, we note that in our leave-one-factor-out analysis where the factor “conjugation” was left out, we attained similar results to that of our full analysis (Supplementary Figure S2). Any misclassification regarding this factor is therefore unlikely to influence our main conclusions beyond those pertaining to the importance of “conjugation” itself.

Our efforts to examine resistance patterns across almost all human bacterial pathogens bring some inherent challenges. First, few data are available for systems where drug resistance is considered uncommon. Second, many bacterial-drug combinations do not have clear definitions of resistance that can be used to objectively categorize whether a particular bacterial isolate is resistant (e.g., CLSI, EUCAST). Third, resistance is rarely studied in pathogens that are difficult to culture. To overcome these challenges, we used two complementary approaches for resistance assessment (ERM and ARM, Figure 1). The ARM approach followed a systematic algorithm for determining which data should and should not be included (Figure 1). Due to the nature of this systematic approach, our data collection is highly repeatable, and we explicitly tested this for a subset of the drug × pathogen combinations by having two authors (A.A. and A.B.) collect and compare data. To avoid bias that may be due to temporal changes in resistance and surveillance efforts, the ARM data were restricted to studies that were published within a fairly narrow time window (January 1, 2016–June 30, 2020). However, some forms of potential bias could not be corrected with this approach. For example, no data were available for some combinations, particularly those where resistance was difficult to measure or considered to be unimportant to human health. Additionally, the availability of ARM data varied widely across different geographical locations, which can introduce bias when resistance frequencies vary by location, as they often do (Klein et al., 2019; OneHealthTrust, 2021). The ERM approach in contrast to the ARM approach was not systematic, but rather relied on individuals to synthesize data across many studies. These studies were not limited to those that fit stringent inclusion and exclusion criteria and thus allowed for the integration of many more studies using many different approaches to characterize resistance (Figure 1). While this approach cannot necessarily correct for all of the biases in the ARM data (which would likely require carefully designed systematic surveillance studies), the ERM approach enabled the coverage of many pathogen-drug combinations that did not have data suitable for the ARM approach. In contrast to the ARM approach, however, the ERM approach used coarse estimates of resistance prevalence and was vulnerable to inherent subjectivity. We attempted to minimize subjectivity by using consensus among multiple authors, but some degree of subjectivity was inevitable (see *Methods* for details). By using both approaches, and carefully considering the strengths and weaknesses of each, we were able to, for the first time, glean novel insights from a wide set of common human bacterial pathogens.

Occasional discrepancies are found between the ARM and ERM data. *Shigella* spp is one of the discrepancies with ERM classification being “Not rare” (score 2) and ARM data showing little resistance. Since ARM focuses on recent papers it is possible that resistance has been changing over time. However, it is also possible that these pathogens should have been classified as “Rare” in the ERM data. The *Shigella* spp data were notably borderline calls in the ERM dataset, with two authors arriving at different scores prior to their consensus call of “Not rare.” *Bacillus anthracis* and *Chlamydia pneumoniae* are two other examples of discrepancies between the ARM and ERM data (ERM scored “Very rare/None” and ARM found a relatively high prevalence of resistance). Here, we note that for both of these pathogens, only one paper was found that met the inclusion criteria of ARM, implying high uncertainty in the estimated prevalence. Since so few data are available, publication bias toward atypical results may arise. Moreover, standardized MIC cut-offs are not available for these pathogens to classify resistance but the papers met our inclusion criteria since they used quantitative approaches (disk diffusion and liquid MIC broth dilution, respectively). These ARM data points may therefore be atypical.

We note that there are at least three sets of additional constraints that were unavoidable in our study. First, the sample size of our study was constrained by virtue of there being a finite number of human bacterial pathogens and drugs used to treat them. It would only be possible to overcome this limitation by expanding to non-human pathogens, but such an expansion would fundamentally change the questions that we could ask. Due to this study size limitation, we cannot consider every factor that might be posited to correlate with drug resistance. The prevalence of resistance may thus correlate with factors not in our analysis including both bacterial features such as genome size (McOrist, 2000), obligate intracellularity (McOrist, 2000), the existence of serotypes (Linares et al., 2010), and drug features such as the level of environmental contamination (Kraemer et al., 2019), average treatment adherence (CDC, 2019b), over-the-counter availability (Rather et al., 2017), the prevalence of use in agriculture, and many others (CDC, 2019b). Second, we assumed that our categorization of drug and pathogen traits was correct even though some traits were difficult to categorize due to limited data availability. Although we used the most current available classification for each factor (Table 1), we note that these classifications may change over time as new research emerges. For instance, the number of human bacterial pathogens considered to be naturally competent has doubled since 1994 (Johnston et al., 2014; Lorenz & Wackernagel, 1994). Third, we have assumed independent random effects of each bacterial species and drug class in our analysis. This may not be correct, if, for example, phylogenetic relatedness between bacterial species caused more closely related bacterial species to evolve resistance at similar rates. However, resistance is often acquired through horizontal gene transfer among distantly related bacterial species (Dzidic & Bedekovic, 2003; Evans et al., 2020; Hedge & Wilson, 2014; Sakoparnig et al., 2021; Schierup & Hein, 2000), reducing the likely impact of phylogenetic relatedness among bacterial species. Likewise, chemical similarities between drug classes may lead to correlations in resistance. While we were unable to fully incorporate this into our analysis, we did explicitly examine the mechanism of drug action (bactericidal/bacteriostatic, see Table 1) in our analyses and did not find any evidence of a significant association with resistance.

Due to the absolute constraints imposed by the fixed number of drugs and fixed number of human bacterial pathogens that exist, we did not perform a Bonferroni correction (Dunn, 1961) for multiple testing even though our analyses included 14 different factors. While this does mean it is possible for our analysis to generate spurious associations between the frequency of resistance and our various factors, we analyzed two sets of data (the ARM and ERM datasets) to partially mitigate the issues with multiple testing. The data in these datasets are not fully independent (Pearson’s product moment correlation, *r* = 0.61), but the similarity of conclusions across both sets of analyses somewhat offsets the likelihood of spurious associations. Nevertheless, for this reason, and because our study is inherently observation, we recommend that our results be viewed as hypotheses that can be challenged through future experiments rather than as the final word on which factors drive the evolution of antibacterial resistance.

The evolution of drug resistance is among the greatest public health threats of modern times. Here, our goal was to (a) document variation in drug resistance across a wide range of bacterial pathogens and the drugs used to treat them, and (b) identify factors that best explain this variation. Our results confirm the importance of previously identified factors like hospital transmission in promoting resistance spread, highlight areas that may require greater focus (e.g., acquired resistance in Gram-positive pathogens), and motivate future work to explore novel associations (e.g., the link between pathogen transmission mode and resistance). Taken together, these findings provide novel insights and opportunities to explore interventions that could lead to more sustainable use of available drugs and manage the emergence and spread of drug resistance.

## Methods

### Data collection

Drug resistance for “pathogen × drug” combinations was measured using two approaches (a) ERM and (b) ARM. For the ERM approach, all 182 “pathogen × drug class” combinations were classified using author consensus into one of three resistance frequency levels with each level assigned a numeric score: Very rare/None (0), Rare (1), or Not rare (2). These categories were defined as described in Figure 1A. In practice, two authors (A.B. and A.A.) independently conducted literature surveys for each “pathogen × drug class” combination to classify them into one of the three ERM levels. A third author (D.A.K.) reviewed all combinations where the calls made by the two authors differed (25% of total calls). A final call was made on these combinations by consensus among all three authors after discussion (Supplementary Table S3). The ARM approach used resistance prevalence data (number of resistant and not resistant isolates) collected from papers available on PubMed using the search term “*PATHOGEN NAME* and *ANTIBIOTIC NAME* resistance” without quotation marks (e.g., Bacillus anthracis and Penicillin resistance). Note that literature searches included specific antibiotics within each drug class. Prevalence data for each combination was derived from the three most recent papers published between January 1, 2016 and June 30, 2020 that determined resistance for at least 10 human clinical isolates using published or quantitative guidelines (the full list of inclusion/exclusion criteria are listed in Figure 1B). In total, ARM resistance prevalence data was obtained for 376 “pathogen × antibiotic” combinations (Supplementary Table S4). These combinations encompassed 149 of the 182 “pathogen × drug class” combinations that were included in the ERM approach, including 49 out of 57 pathogens and all 15 drug classes.

### Data collection for drug and pathogen characteristics

To identify the factors that best explain the observed variation in resistance, 14 different factors believed to be associated with resistance evolution were investigated (Table 1, Supplementary Table S5). Focal pathogen traits include whether the pathogen is “nosocomial” (i.e., transmitted in hospital settings), if the pathogen has animal (“zoonosis”), human microbiome (“commensal”), or “environmental reservoirs,” whether the pathogen is Gram positive, Gram negative, anaerobic, or other (“pathogen type”) (Cornelissen & Hobbs, 2020), whether it has direct or indirect transmission (“transmission type”) (CDC, 2012), and whether human-to-human transmission has been documented (“human transmission”). Reservoirs were not treated as mutually exclusive. Pathogen type was treated as mutually exclusive such that pathogens classified as anaerobic were not also classified as Gram positive or negative. In cases of pathogens that are transmitted in multiple ways, we classified transmission as direct or indirect based on the most common mode of transmission. Two modes of horizontal gene transfer, “natural competence” and “conjugation,” were also examined. Consistent with currently accepted standards in the field, pathogens were noted to have natural competence or conjugation if evidence was available that a pathogen has the requisite genetic machinery and/or whether such events have been directly or indirectly observed outside of an artificial setting. Drug factors examined in our analyses included how long a drug has been in use (“drug date”; noted as the date of earliest publication for each drug class name or antibiotic name on PubMed), “global drug use” (determined by calculating the national total for defined daily doses of drug used in 2015 averaged over the three countries that report the highest drug usage, i.e., China, India, and the USA) (OneHealthTrust, 2023; World Bank Group, 2021), and whether the drug is bactericidal (killing) or bacteriostatic (growth-inhibiting). For drugs that can have either mechanism depending on dose, the more common mode was chosen. For example, macrolides, which are primarily bacteriostatic but might act bactericidal in some cases, were classified as bacteriostatic. Finally, differences in research effort devoted to different pathogens and drugs were accounted for by recording the total number of results returned on the database PubMed when conducting a general search for each of the 57 pathogen names, 15 drug classes, and 53 antibiotics.

### Statistical analyses

All statistical analyses were performed in RStudio (Version 1.2.5033) (R Core Team, 2019). Mixed-effects models were used to determine the factors that best explained the variation observed in drug resistance. Models were fit with the “lme4” package (Bates et al., 2015) in RStudio using the functions “lmer” for the ERM data and “glmer” with option “family=binomial” for the ARM data.

#### Single-factor analyses

Linear mixed-effects models with normally distributed error were used to analyze the ERM data, while generalized linear mixed-effects models with a logit link were used for the ARM analyses. We used these model structures because the ARM data are inherently binomial (number of resistant isolates vs. number of non-resistant isolates), whereas the ERM data are not. For the single-factor analyses (Figure 4), the ERM data were analyzed using mixed-effects models with single factors as fixed effects and “pathogen name” and “drug class” included in each model as random effects. The ARM data were analyzed using binomial generalized linear mixed-effects models with single factors again used as fixed effects and “pathogen name,” “drug class,” and “antibiotic” as random effects.

#### Best-fitting models

To assess the relative importance of 14 different factors implicated in resistance evolution, each of the ERM and ARM datasets was fit to a suite of mixed-effects models representing all possible combinations of the 14 factors as fixed effects (16,384 total models for each dataset). As in the single-factor analysis, the ERM data were analyzed using linear mixed-effects models, while the ARM data were analyzed using binomial generalized linear mixed-effects models. The ERM data included “pathogen name” and “drug class” as random effects. The ARM data included “pathogen name,” “drug class,” and “antibiotic” as random effects. The models with the lowest AIC score for each respective dataset (ERM and ARM) were identified as the best-fitting models. The factors included in these best models are listed in Figure 7.

#### ΔAIC scores and AIC weights

The relative importance of the 14 different factors was assessed using two AIC-based metrics: ΔAIC score and AIC weight. AIC is a model selection tool that takes into account model fit and model complexity, with preference given to simple models (i.e., models that include as few factors as possible) that explain the data well. ΔAIC is the difference in AIC scores between the best model and a focal model. The ΔAIC score represents the degree of support for the focal model relative to the best model, where smaller values indicate stronger model support (Burnham & Anderson, 1998). It can also be used to determine the support for a particular factor in the best model by either removing a factor that was already in the model or adding a factor that was not part of the model and measuring the change in the AIC score. For factors already included in the best model, a large change in AIC (large ΔAIC) following the removal of a factor indicates that the factor is important to explaining the data, whereas a small change in AIC indicates that explanatory value of the factor just barely outweighs the penalty for increasing the complexity of the model. For factors not included in the best model, a large change in AIC indicates that the factor has little explanatory power whereas a smaller change in AIC (small ΔAIC) following the introduction of a factor indicates that the explanatory value of the factor partially compensates for the increased model complexity, although not sufficiently to be included in the best model.

The AIC weight of a model is the probability of that model being the “true” model given that the set of all models contains the true model (Burnham & Anderson, 1998). The AIC weight of a factor is similarly the probability that a particular factor is included in the true model. We calculate AIC weights for each factor by summing the AIC weights for each model that contains the focal factor. In practice, these AIC weights are calculated as


$$\begin{array}{l} AICweight=\frac{\underset{j}{\sum }e^{-\;\Delta \;AIC_{j}/2}}{\underset{i}{\sum }e^{-\;\Delta \;AIC_{i}/2}} \end{array}$$

where *j* represents the set of models that include the factor of interest and *i* represents the set of all the models in the analysis. AIC weights greater than 0.5 for a particular factor imply that it is more likely than not to be included in the true model, demonstrating support for the factor. Larger AIC weights demonstrate more support.

#### Leave-one-factor-out analysis

To test for robustness and potential confounding in the importance of individual factors, we used AIC weights measured in 14 different scenarios. In each scenario, one of the 14 factors was eliminated from analysis and the AIC weights of the remaining 13 factors were calculated as described above. Calculated AIC weights should remain nearly constant if the analysis is robust to removing factors. Large changes in AIC weights indicate potential confounding between factors.

#### Model-averaged parameter estimates

Model-averaged parameter estimates for each factor were calculated across all models that included the factor to estimate effect sizes for each focal factor. In practice, this was performed using the “modavg” package (Mazerolle, 2023) in R Studio. For the ARM dataset, effect sizes were translated to the odds ratio scale by calculating the exponential value of the mean and the confidence intervals. Factors with 95% confidence intervals not overlapping “0” for the ERM dataset and “1” for the ARM dataset were considered statistically significant. The mean and 95% CI of parameter estimates are plotted in Figure 6.

## Supplementary Material

qrae005_suppl_Supplementary_Material

## Data Availability

The code and data are accessible at https://github.com/dkenned1/DrugResistanceReview2024.
