# Supplementary material for: Exceptions to the rule: When does resistance evolution not undermine antibiotic therapy in human bacterial infections?
Source: Evol Lett. 2024 Mar 2;8(4):478–93. doi: 10.1093/evlett/qrae005 (PMC11291617; doi:10.1093/evlett/qrae005)
Supplement: qrae005_suppl_Supplementary_Material [file qrae005_suppl_supplementary_material.docx]

**Supplemental Figure S1**

A.


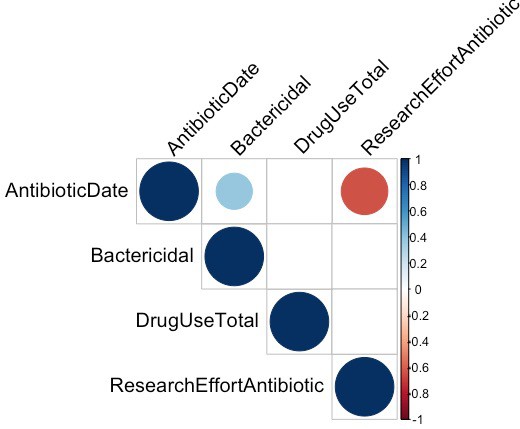


B. Drug Class Traits (n = 15)


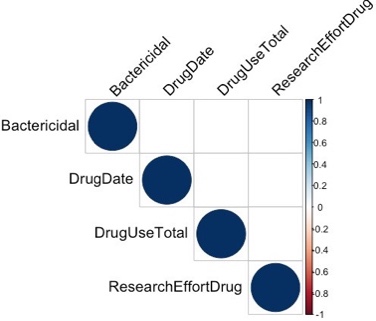


C. Antibiotic Traits (n = 53)


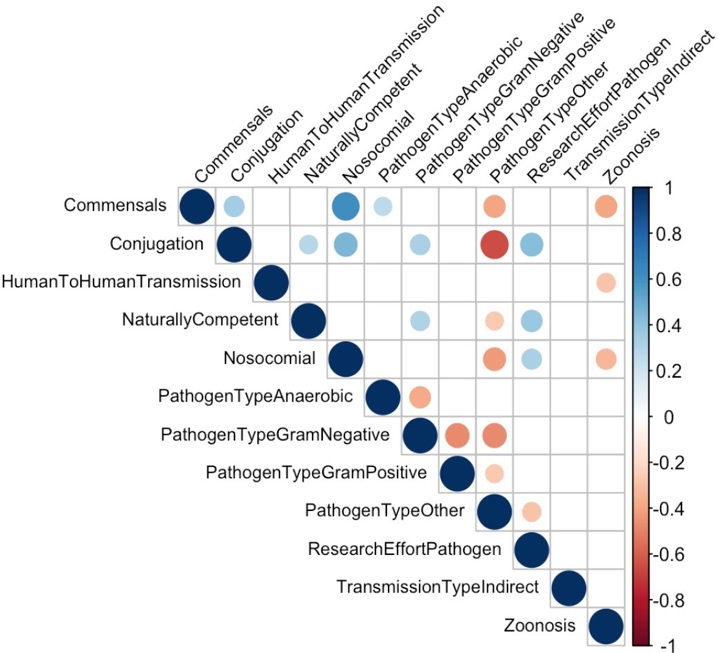


A. Pathogen Traits (n = 57)

**Supplemental Figure S1: Significant correlations between pathogen traits, drug class factors, and antibiotic factors.** Significant correlations (p < 0.05) between different pathogen traits (panel A), between different drug class traits used in ERM dataset (panel B), and between different antibiotic traits used in the ARM dataset (panel C). Color and size of the symbols represent the direction and size of correlations. The number of pathogens and drugs with each trait in each category is provided in Supplemental Table S1.

**Supplemental Figure S2**

**Supplemental Figure S2: Relative importance of factors is robust to the exclusion of other factors from analysis.** To examine the robustness of the relative order of factor weights (Main Text, Figure 5), the AIC weights of all factors were calculated in 14 different scenarios. In each scenario, one of the 14 factors was eliminated and the analysis was rerun to calculate the AIC weights for the 13 remaining factors. Panels A and B represent results from the Expert Review Method (ERM) and Algorithmic Review Method (ARM) respectively. The x-axis lists the factor excluded from each analysis. In both panels the y-axis represents AIC weights with values greater than 0.5 (marked by dashed line). The unique color-symbol combinations represent each factor. Note that the legend is inherently built into this figure since the points with zero AIC weight at each location on the x-axis depict the color-symbol combination for the model lacking that factor. Where no factors were excluded (None) the AIC weights represent results from the full analysis including all 14 factors, also shown in Figure 5. In both ERM and ARM datasets, the AIC weight of ‘natural competence’ increased substantially when ‘commensal’ was removed from the analysis.


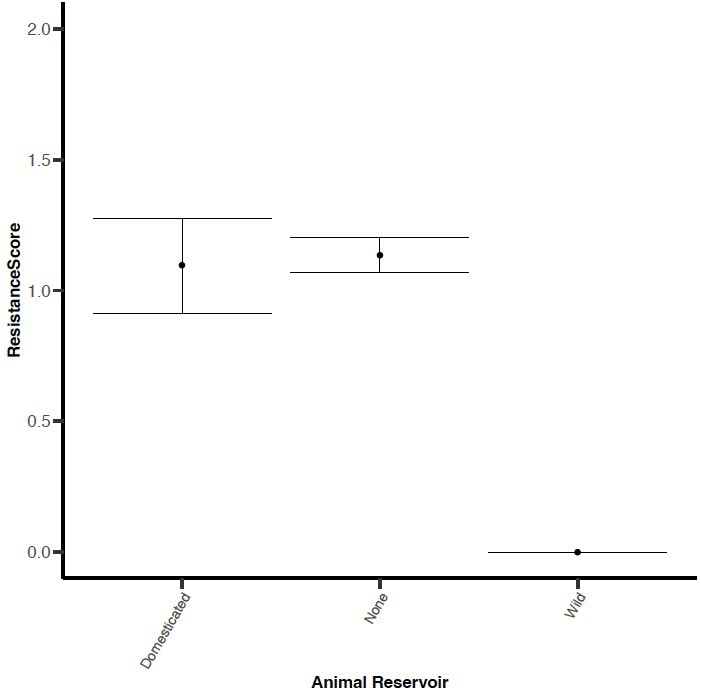


**Supplemental Figure S3: Resistance scores of zoonotic pathogens subdivided into wild or domesticated reservoirs.** Zoonotic pathogens with wild animal reservoirs have significantly lower frequencies of resistance than zoonotic pathogens with domesticated reservoirs, and non-zoonotic pathogens. Mean +/- 1 standard error of resistance score data from the Expert Review Method data classified by type of animal reservoir (F(2,179) = 12.72, p < 0.001).

## Supplemental Table S1: Expert Review Method Data

| Category | Pathogens | Drug Classes | Pathogen x Drug combinations |
| --- | --- | --- | --- |
| Pathogens Drug Classes  Combinations | 57 | 15 | 182 |
| Nosocomial | | | |
| Yes  No | 25  32 | N/A  N/A | 96  86 |
| Zoonosis | | | |
| Yes  No | 12  45 | N/A  N/A | 34  148 |
| Commensal | | | |
| Yes  No | 30  27 | N/A  N/A | 121  61 |
| Naturally competent | | | |
| Yes  No | 12  45 | N/A  N/A | 48  134 |
| Conjugation | | | |
| Yes  No | 44  13 | N/A  N/A | 150  32 |
| Human-human transmission | | | |
| Yes  No | 45  12 | N/A  N/A | 136  46 |
| Transmission mode | | | |
| Direct  Indirect | 33  24 | N/A  N/A | 117  65 |

| Category | Pathogens |  | Drug Classes | | Pathogen x Drug combinations |
| --- | --- | --- | --- | --- | --- |
| Pathogen type | | | | | |
| Gram positive | 11 |  | N/A |  | 33 |
| Gram negative | 26 |  | N/A |  | 89 |
| Anaerobic | 8 |  | N/A |  | 35 |
| Other | 12 |  | N/A |  | 25 |
| Environmental reservoir | | | | | |
| Yes  No | 12  45 |  | N/A  N/A |  | 26  156 |
| Drug mechanism | | | | | |
| Bactericidal  Bacteriostatic | N/A  N/A |  | 13  2 |  | 136  46 |
| Research effort pathogens (log_10_ transformed number of published papers on PubMed) | | | | | |
| 56 unique values | Mean  3.85 | Min.  1.0 | 1st Qu.  3.53 | Median  3.85 | 3rd Qu. Max.  4.24 5.59 |
| Research effort drugs (log_10_ transformed number of published papers on PubMed) | | | | | |
| 15 unique values | Mean  3.65 | Min.  1.25 | 1st Qu.  3.23 | Median  3.98 | 3rd Qu. Max.  4.24 4.83 |
| Drug date (year) | | | | | |
| 14 unique dates | Mean  1970 | Min.  1944 | 1st Qu.  1950 | Median  1974 | 3rd Qu. Max.  1985 2008 |
| Drug use (log_10_ transformed) | | | | | |
| 10 unique values  (Generation-specific cephalosporin use not available) | Mean  9.42 | Min.  7.73 | 1st Qu.  9.39 | Median  9.63 | 3rd Qu. Max.  10.02 10.02 |

Supplemental Table S2: Algorithmic Review Data

| Category | Pathogens | Antibiotics | Pathogen x Antibiotic combinations |
| --- | --- | --- | --- |
| Pathogens  Antibiotics Combinations | 49 | 53 | 376 |
| Nosocomial | | | |
| Yes | 25 | N/A | 238 |
| No | 24 | N/A | 138 |
| Zoonosis | | | |
| Yes | 7 | N/A | 52 |
| No | 42 | N/A | 324 |
| Commensal | | | |
| Yes | 29 | N/A | 281 |
| No | 20 | N/A | 95 |
| Naturally competent | | | |
| Yes | 12 | N/A | 140 |
| No | 37 | N/A | 236 |
| Conjugation | | | |
| Yes | 41 | N/A | 343 |
| No | 8 | N/A | 33 |
| Human-human transmission | | | |
| Yes | 40 | N/A | 312 |
| No | 9 | N/A | 64 |
| Transmission mode | | | |
| Direct | 25 | N/A | 181 |
| Indirect | 24 | N/A | 195 |

| Category Pathogens Drug Classes Pathogen x Antibiotic  combinations | | | | | | |
| --- | --- | --- | --- | --- | --- | --- |
| Pathogen type | | | | | | |
| Gram positive | 11 |  | N/A |  | 69 |  |
| Gram negative | 24 |  | N/A |  | 221 |  |
| Anaerobic | 7 |  | N/A |  | 58 |  |
| Other | 7 |  | N/A |  | 28 |  |
| Environmental reservoir | | | | | | |
| Yes | 10 |  | N/A |  | 50 |  |
| No | 39 |  | N/A |  | 326 |  |
| Drug mechanism | | | | | | |
| Bactericidal | N/A |  | 44 |  | 306 |  |
| Bacteriostatic | N/A |  | 9 |  | 70 |  |
| Research effort pathogens (log_10_ transformed) | | | | | | |
| 46 unique values | Mean  4.11 | Min.  2.03 | 1st Qu.  3.76 | Median  4.12 | 3rd Qu.  4.53 | Max.  5.59 |
| Research effort antibiotics (log_10_ transformed) | | | | | | |
| 51 unique values | Mean  3.94 | Min.  2.18 | 1st Qu.  3.68 | Median  4.00 | 3rd Qu.  4.25 | Max.  5.04 |
| Antibitoic date | | | | | | |
| 33 unique dates | Mean  1975 | Min.  1932 | 1st Qu.  1968 | Median  1980 | 3rd Qu.  1986 | Max.  2010 |
| Drug use (log transformed) | | | | | | |
| 10 unique values  (Generation-specific cephalosporin use not available) | Mean  9.49 | Min.  7.73 | 1st Qu.  9.39 | Median  9.63 | 3rd Qu.  10.02 | Max.  10.02 |

# Supplemental Table S3 : Expert Review Method raw data and citations

| **Pathogen Name** | **Drug Class** | **Classification** | **Citations** |
| --- | --- | --- | --- |
| *Acinetobacter* spp | Carbapenems | **Not Rare** | 1–5 |
| *Actinomyces spp* | Beta-lactams /Penicillins | **None/ Very Rare** | 6–10 |
| *Actinomyces spp* | Carbapenems | **None/ Very Rare** | 9,11 |
| *Actinomyces spp* | Macrolides | **None/ Very Rare** | 9,11 |
| *Bacillus anthracis* | Beta-lactams /Penicillins | **None/ Very Rare** | 12–15 |
| *Bacillus anthracis* | Fluoroquinolones | **None/ Very Rare** | 12,16,17 |
| *Bacillus anthracis* | Tetracyclines | **None/ Very Rare** | 3,12,18 |
| *Bacteroides spp* | Beta-lactams/ Penicillins | **Not Rare** | 19–22 |
| *Bacteroides spp* | Carbapenems | **Rare** | 19,20,23–27 |
| *Bacteroides spp* | Cephalosporins (1st gen) | **Not Rare** | 22,28,29 |
| *Bacteroides spp* | Cephalosporins (2nd gen) | **Rare** | 21,22,27,28,30–32 |
| *Bacteroides spp* | Cephalosporins (3rd gen) | **Not Rare** | 25,28,33,34 |
| *Bacteroides spp* | Macrolides | **Not Rare** | 19,20,28,35,36 |
| *Bacteroides spp* | Nitroimidazoles | **Rare** | 20,28,37,38 |
| *Bacteroides spp* | Tetracyclines | **Not Rare** | 19,20,23,30 |
| *Bordetella pertussis* | Macrolides | **Not Rare** | 39–42 |
| *Borrelia burgdorferi* | Macrolides | **None/ Very Rare** | 43–46 |
| *Borrelia burgdorferi* | Tetracyclines | **None/ Very Rare** | 43,44 |
| *Brucella spp* | Aminoglycosides | **None/ Very Rare** | 47–49 |
| *Brucella spp* | Fluoroquinolones | **None/ Very Rare** | 16,50 |
| *Brucella spp* | Rifampin | **Rare** | 47,49,50 |
| *Brucella spp* | Tetracyclines | **None/ Very Rare** | 16,47,50 |
| *Brucella spp* | Trimethoprim-sulfamethoxazole | **Rare** | 47,50 |
| *Campylobacter jejuni* | Fluoroquinolones | **Not Rare** | 3,51,52 |
| *Campylobacter jejuni* | Macrolides | **Rare** | 51,53,54 |
| *Chlamydia pneumoniae* | Macrolides | **None/ Very Rare** | 55–58 |
| *Chlamydia pneumoniae* | Tetracyclines | **None/ Very Rare** | 58–60 |
| *Chlamydia psittaci* | Macrolides | **None/ Very Rare** | 55,61,62 |

| *Chlamydia psittaci* | Tetracyclines | **None/ Very Rare** | 62 |
| --- | --- | --- | --- |
| *Chlamydia trachomatis* | Macrolides | **None / Very Rare** | 55,56,63,64 |
| *Chlamydia trachomatis* | Tetracyclines | **None / Very Rare** | 56,58,59,64 |
| *Citrobacter* spp | Aminoglycosides | **Rare** | 65–68 |
| *Citrobacter* spp | Beta-lactams /Penicillins | **Not Rare** | 65,67,69 |
| *Citrobacter* spp | Carbapenems | **Rare** | 65,67,69–72 |
| *Citrobacter* spp | Cephalosporins (2nd gen) | **Not Rare** | 67,69,73 |
| *Citrobacter* spp | Cephalosporins (3rd gen) | **Not Rare** | 67,69,70,74,75 |
| *Citrobacter* spp | Macrolides | **None/ Very Rare** | 67 |
| *Citrobacter* spp | Tetracyclines | **Not Rare** | 67,76–78 |
| *Citrobacter* spp | Trimethoprim-sulfamethoxazole | **Rare** | 65–67,72,79 |
| *Clostridium difficile* | Vancomycin | **Rare** | 80–83 |
| *Clostridium perfringens* | Beta-lactams /Penicillins | **Rare** | 81,84–88 |
| *Clostridium perfringens* | Tetracyclines | **Not Rare** | 81,84,89,90 |
| *Clostridium spp* | Carbapenems | **None/ Very Rare** | 7,86,91 |
| *Clostridium spp* | Tetracyclines | **None/ Very Rare** | 28,92 |
| *Clostridium spp* | Beta-lactams /Penicillins | **Rare** | 7,86,91–93 |
| *Clostridium tetani* | Tetracyclines | **None/ Very Rare** | 94 |
| *Corynebacterium diptheriae* | Beta-lactams /Penicillins | **Rare** | 95–97 |
| *Corynebacterium diptheriae* | Macrolides | **Rare** | 95–97 |
| *Enterobacter aerogenes* | Carbapenems | **Rare** | 98–100 |
| *Enterobacter aerogenes* | Cephalosporins (2nd gen) | **Not Rare** | 98,101 |
| *Enterobacter aerogenes* | Cephalosporins (3rd gen) | **Not Rare** | 98,101 |
| *Enterobacter aerogenes* | Cephalosporins (4th gen) | **Rare** | 98,100,102,103 |
| *Enterobacter aerogenes* | Fluoroquinolones | **Rare** | 100,101,104–106 |
| *Enterococcus faecalis* | Aminoglycosides | **Not Rare** | 107,108 |
| *Enterococcus faecalis* | Carbapenems | **Rare** | 109–114 |
| *Escherichia coli (ETEC)* | Carbapenems | **Rare** | 115–117 |

| *Escherichia coli (ETEC)* | Cephalosporins (1st gen) | **Not Rare** | 115 |
| --- | --- | --- | --- |
| *Escherichia coli (ETEC)* | Cephalosporins (2nd gen) | **Not Rare** | 118,119 |
| *Escherichia coli (ETEC)* | Cephalosporins (3rd gen) | **Not Rare** | 115,118 |
| *Escherichia coli (ETEC)* | Cephalosporins (4th gen) | **Not Rare** | 119–121 |
| *Escherichia coli (ETEC)* | Cephalosporins (5th gen) | **Not Rare** | 122–124 |
| *Escherichia coli (ETEC)* | Fluoroquinolones | **Not Rare** | 125 |
| *Francisella tularensis* | Aminoglycosides | **None/ Very Rare** | 126–128 |
| *Francisella tularensis* | Fluoroquinolones | **None/ Very Rare** | 126,129,130 |
| *Francisella tularensis* | Tetracyclines | **None/ Very Rare** | 128,131 |
| *Fusobacterium spp* | Beta-lactams /Penicillins | **Rare** | 28,92,132–134 |
| *Fusobacterium spp* | Carbapenems | **None/ Very Rare** | 28,92,134,135 |
| *Fusobacterium spp* | Cephalosporins (2nd gen) | **None/ Very Rare** | 7,28,133,134,136–138 |
| *Gardnerella vaginalis* | Carbapenems | **None/ Very Rare** | 139 |
| *Gardnerella vaginalis* | Macrolides | **Not Rare** | 139–141 |
| *Gardnerella vaginalis* | Metronidazole | **Not Rare** | 139–141 |
| *GPAC* | Carbapenems | **None/ Very Rare** | 142–145 |
| *GPAC* | Cephalosporins (1st gen) | **None/ Very Rare** | 142,146 |
| *GPAC* | Cephalosporins (2nd gen) | **None/ Very Rare** | 142,144,145 |
| *GPAC* | Cephalosporins (3rd gen) | **None/ Very Rare** | 142,146 |
| *GPAC* | Macrolides | **Not Rare** | 142–144 |
| *GPAC* | Metronidazole | **Rare** | 142–145,147 |
| *GPAC* | Tetracyclines | **Not Rare** | 142,144,148 |
| *GPAC* | Beta-lactams /Penicillins | **Rare** | 142–145 |
| *Haemophilus influenzae* | Beta-lactams /Penicillins | **Not Rare** | 149,150 |
| *Haemophilus influenzae* | Carbapenems | **None / Very Rare** | 151–153 |
| *Haemophilus influenzae* | Cephalosporins (2nd gen) | **Not Rare** | 151,153–159 |
| *Haemophilus influenzae* | Cephalosporins (3rd gen) | **Rare** | 152,153,156,157,160 |
| *Haemophilus influenzae* | Cephalosporins (5th gen) | **Very Rare/ None** | 122,151,161 |

| *Haemophilus influenzae* | Fluoroquinolones | **Rare** | 150,151,153,154,162 |
| --- | --- | --- | --- |
| *Haemophilus influenzae* | Macrolides | **Rare** | 150,152–154,160 |
| *Haemophilus influenzae* | Trimethoprim-sulfamethoxazole | **Not Rare** | 151,153,155,160 |
| *Klebsiella oxytoca* | Cephalosporins (5th gen) | **Rare** | 163–165 |
| *Klebsiella pneumoniae* | Carbapenems | **Not Rare** | 166–170 |
| *Klebsiella pneumoniae* | Cephalosporins (1st gen) | **Not Rare** | 171–173 |
| *Klebsiella pneumoniae* | Cephalosporins (2nd gen) | **Not Rare** | 167,172–174 |
| *Klebsiella pneumoniae* | Cephalosporins (3rd gen) | **Not Rare** | 166,167,175,176 |
| *Klebsiella pneumoniae* | Cephalosporins (4th gen) | **Not Rare** | 166,167,170 |
| *Klebsiella pneumoniae* | Cephalosporins (5th gen) | **Not Rare** | 171,177,178 |
| *Klebsiella pneumoniae* | Fluoroquinolones | **Not Rare** | 170,179,180 |
| *Klebsiella spp* | Aminoglycosides | **Not Rare** | 68,181–183 |
| *Legionella pneumophila* | Fluoroquinolones | **Rare** | 184–186 |
| *Legionella pneumophila* | Macrolides | **Rare** | 184–186 |
| *Leptospira interrogans* | Beta-lactams /Penicillins | **None / Very Rare** | 187,188 |
| *Leptospira interrogans* | Cephalosporins (3rd gen) | **None/ Very Rare** | 187 |
| *Leptospira interrogans* | Tetracyclines | **None/ Very Rare** | 187 |
| *Listeria monocytogenes* | Carbapenems | **None/ Very Rare** | 178,189–191 |
| *Moraxella catarrhalis* | Beta-lactams /Penicillins | **Not Rare** | 192–194 |
| *Moraxella catarrhalis* | Cephalosporins (2nd gen) | **Rare** | 178,189,191,195–198 |
| *Moraxella catarrhalis* | Fluoroquinolones | **Rare** | 189–191,195 |
| *Moraxella catarrhalis* | Macrolides | **Rare** | 178,189,191,196–198 |
| *Moraxella catarrhalis* | Tetracyclines | **Rare** | 178,189–191 |
| *Mycoplasma pneumoniae* | Macrolides | **Not Rare** | 199,200 |
| *Mycoplasma pneumoniae* | Tetracyclines | **Very Rare/ None** | 199,200 |
| *Neisseria gonorrhoeae* | Carbapenems | **Very Rare/ None** | 201 |
| *Neisseria gonorrhoeae* | Cephalosporins (2nd gen) | **Very Rare/ None** | 202,203 |
| *Neisseria gonorrhoeae* | Cephalosporins (3rd gen) | **Rare** | 202–206 |

| *Neisseria meningitidis* | Beta-lactams /Penicillins | **Rare** | 207–213 |
| --- | --- | --- | --- |
| *Neisseria meningitidis* | Carbapenems | **None / Very Rare** | 214–216 |
| *Neisseria meningitidis* | Cephalosporins (3rd gen) | **Rare** | 209–212,214,217–219 |
| *Nocardia spp* | Carbapenems | **Not Rare** | 220–225 |
| *Nocardia spp* | Trimethoprim-sulfamethoxazole | **Rare** | 220,222,226,227 |
| *Non-typhoidal Salmonella* | Cephalosporins (3rd gen) | **Rare** | 228,229 |
| *Non-typhoidal Salmonella* | Macrolides | **Rare** | 230–232 |
| *Propiniobacterium acnes* | Beta-lactams /Penicillins | **Very Rare/ None** | 233–236 |
| *Propiniobacterium acnes* | Carbapenems | **Very Rare / None** | 85,134,235,237,238 |
| *Propiniobacterium acnes* | Cephalosporins (1st gen) | **Very Rare / None** | 234,235,239,240 |
| *Propiniobacterium acnes* | Cephalosporins (2nd gen) | **Very Rare / None** | 237,239,241,242 |
| *Propiniobacterium acnes* | Cephalosporins (3rd gen) | **Very Rare / None** | 85,235,237,240 |
| *Propiniobacterium acnes* | Macrolides | **Not Rare** | 243,244 |
| *Propiniobacterium acnes* | Metronidazole | **Not Rare** | 237,239,242,245 |
| *Propiniobacterium acnes* | Tetracyclines | **Not Rare** | 243,246–248 |
| *Propiniobacterium acnes* | Trimethoprim-sulfamethoxazole | **Not Rare** | 247–249 |
| *Proteus mirabilis* | Cephalosporins (1st gen) | **Not Rare** | 250–252 |
| *Proteus mirabilis* | Cephalosporins (2nd gen) | **Not Rare** | 250–253 |
| *Proteus mirabilis* | Cephalosporins (3rd gen) | **Rare** | 250–254 |
| *Proteus mirabilis* | Cephalosporins (4th gen) | **Rare** | 250–252,254,255 |
| *Proteus mirabilis* | Fluoroquinolones | **Not Rare** | 250–253 |
| *Proteus spp* | Carbapenems | **Rare** | 256–259 |
| *Providencia spp* | Carbapenems | **Very Rare / None** | 70,260,261 |
| *Pseudomonas aeruginosa* | Aminoglycosides | **Not Rare** | 262–264 |
| *Pseudomonas aeruginosa* | Carbapenems | **Not Rare** | 262,263,265,266 |
| *Pseudomonas aeruginosa* | Cephalosporins (3rd gen) | **Not Rare** | 4,265,267 |
| *Pseudomonas aeruginosa* | Cephalosporins (4th gen) | **Not Rare** | 262–264 |
| *Rickettsia rickettsii* | Tetracyclines | **Very Rare / None** | 268,269 |

| *Salmonella typhi* | Carbapenems | **Very Rare / None** | 270,271 |
| --- | --- | --- | --- |
| *Salmonella typhi* | Cephalosporins (3rd gen) | **Rare** | 270–273 |
| *Salmonella typhi* | Fluoroquinolones | **Not Rare** | 272–274 |
| *Salmonella typhi* | Macrolides | **Rare** | 270–272 |
| *Serratia marcescens* | Carbapenems | **Rare** | 275–277 |
| *Serratia marcescens* | Cephalosporins (3rd gen) | **Not Rare** | 276–279 |
| *Serratia marcescens* | Fluoroquinolones | **Rare** | 277,279 |
| *Shigella* species | Fluoroquinolones | **Not Rare** | 280–285 |
| *Shigella species* | Macrolides | **Not Rare** | 280,281,286 |
| *Staphylococcus aureus* | Carbapenems | **Not Rare** | 287–290 |
| *Staphylococcus aureus* | Cephalosporins (1st gen) | **Not Rare** | 287,288,290 |
| *Staphylococcus aureus* | Cephalosporins (2nd gen) | **Not Rare** | 287,288,291–296 |
| *Staphylococcus aureus* | Cephalosporins (4th gen) | **Not Rare** | 287,288,291,297 |
| *Staphylococcus aureus* | Cephalosporins (5th gen) | **Rare** | 178,288,290,292–296 |
| *Staphylococcus aureus* | Macrolides | **Not Rare** | 298–300 |
| *Staphylococcus epidermidis* | Carbapenems | **Rare** | 301–304 |
| *Staphylococcus epidermidis* | Cephalosporins (1st gen) | **Not Rare** | 302,304 |
| *Streptococcus agalactiae* | Aminoglycosides | **Very Rare / None** | 305–307 |
| *Streptococcus agalactiae* | Cephalosporins (5th gen) | **Very Rare / None** | 165,308,309 |
| *Streptococcus pneumoniae* | Beta-lactams /Penicillins | **Not Rare** | 310–312 |
| *Streptococcus pneumoniae* | Carbapenems | **Not Rare** | 287,310,313,314 |
| *Streptococcus pneumoniae* | Cephalosporins (2nd gen) | **Not Rare** | 312,313,315,316 |
| *Streptococcus pneumoniae* | Cephalosporins (3rd gen) | **Rare** | 151,310,317 |
| *Streptococcus pneumoniae* | Cephalosporins (4th gen) | **Rare** | 313,318–320 |
| *Streptococcus pneumoniae* | Macrolides | **Not Rare** | 310,321–325 |
| *Streptococcus pyogenes* | Beta-lactams /Penicillins | **None / Very Rare** | 309,326,327 |
| *Streptococcus pyogenes* | Cephalosporins (1st gen) | **None / Very Rare** | 328,329 |
| *Streptococcus pyogenes* | Cephalosporins (2nd gen) | **None / Very Rare** | 295,315,330,331 |

| *Streptococcus pyogenes* | Cephalosporins (3rd gen) | **None / Very Rare** | 309,326,331,332 |
| --- | --- | --- | --- |
| *Streptococcus pyogenes* | Cephalosporins (4th gen) | **None / Very Rare** | 333,334 |
| *Streptococcus pyogenes* | Cephalosporins (5th gen) | **None / Very Rare** | 165,308,309 |
| *Streptococcus pyogenes* | Macrolides | **Not Rare** | 326–328,333 |
| *Streptococcus viridans* | Beta-lactams /Penicillins | **Rare** | 300,335–340 |
| *Streptococcus viridans* | Cephalosporins (4th gen) | **Rare** | 341–343 |
| *Treponema pallidum* | Beta-lactams /Penicillins | **None / Very Rare** | 344–346 |
| *Treponema pallidum* | Macrolides | **Not Rare** | 344,347,348 |
| *Treponema pallidum pertenue* | Beta-lactams /Penicillins | **None / Very Rare** | 349,350 |
| *Ureaplasma urealyticum* | Fluoroquinolones | **Not Rare** | 351–353 |
| *Ureaplasma urealyticum* | Macrolides | **Not Rare** | 351–354 |
| *Ureaplasma urealyticum* | Tetracyclines | **Rare** | 351–356 |
| *Vibrio cholerae* | Tetracyclines | **Not Rare** | 357–359 |
| *Yersinia pestis* | Aminoglycosides | **None / Very Rare** | 360,361 |
| *Yersinia pestis* | Tetracyclines | **None / Very Rare** | 16,360,361 |

1. Chatterjee, S. *et al.* Carbapenem resistance in Acinetobacter baumannii and other acinetobacter spp. causing neonatal sepsis: Focus on NDM-1 and its linkage to ISAba125. *Front. Microbiol.* **7**, 1– 13 (2016).
2. Alvarez-Uria, G. & Midde, M. Trends and factors associated with antimicrobial resistance of Acinetobacter spp. invasive isolates in Europe: A country-level analysis. *J. Glob. Antimicrob. Resist.* **14**, 29–32 (2018).
3. US Department of Health and Human Services & CDC. Antibiotic Resistance Threats in the United States. *Centers Dis. Control Prev.* 1–113 (2019).
4. Potron, A., Poirel, L. & Nordmann, P. Emerging broad-spectrum resistance in Pseudomonas aeruginosa and Acinetobacter baumannii: Mechanisms and epidemiology. *Int. J. Antimicrob. Agents* **45**, 568–585 (2015).
5. Alexander Viehman, J. *et al.* Treatment Options for Carbapenem-Resistant and Extensively Drug- Resistant Acinetobacter baumannii Infections. *Drugs* **74**, 1315–1333 (2014).
6. Barberis, C. *et al.* Antimicrobial susceptibility of clinical isolates of Actinomyces and related genera reveals an unusual clindamycin resistance among Actinomyces urogenitalis strains. *J. Glob. Antimicrob. Resist.* **8**, 115–120 (2017).
7. Marchand-Austin, A. *et al.* Antimicrobial susceptibility of clinical isolates of anaerobic bacteria in

Ontario, 2010-2011. *Anaerobe* **28**, 120–125 (2014).

1. Smith, A. J., Hall, V., Thakker, B. & Gemmell, C. G. Antimicrobial susceptibility testing of Actinomyces species with 12 antimicrobial agents. *J. Antimicrob. Chemother.* **56**, 407–409 (2005).
2. Steininger, C. & Willinger, B. Resistance patterns in clinical isolates of pathogenic Actinomyces species. *J. Antimicrob. Chemother.* **71**, 422–427 (2016).
3. Ready, D. *et al.* Composition and antibiotic resistance profile of microcosm dental plaques before and after exposure to tetracycline. *J. Antimicrob. Chemother.* **49**, 769–775 (2002).
4. Valour, F. *et al.* Actinomycosis: Etiology, clinical features, diagnosis, treatment, and management.

*Infect. Drug Resist.* **7**, 183–197 (2014).

1. Bryskier, A. Bacillus anthracis and antibacterial agents. *Clin. Microbiol. Infect.* **8**, 467–478 (2002).
2. Gargis, A. S. *et al.* crossm Analysis of Whole-Genome Sequences for the Prediction of. *Am. Soc. Microbiol.* 1–14 (2018).
3. Lalitha, M. . & Thomas, M. K. Penicillin Resistance in Bacillus Anthracis. *Lancet* **349**, 1522 (1997).
4. Turnbull, P. C. B. *et al.* MICs of Selected Antibiotics for Bacillus anthracis, Bacillus cereus, Bacillus thuringiensis, and Bacillus mycoides from a Range of Clinical and Environmental Sources as Determined by the Etest. *J. Clin. Microbiol.* **42**, 3626–3634 (2004).
5. Brouillard, J. E., Terriff, C. M., Tofan, A. & Garrison, M. W. Antibiotic selection and resistance issues with fluoroquinolones and doxycycline against bioterrorism agents. *Pharmacotherapy* **26**, 3–14 (2006).
6. Frean, J., Klugman, K. P., Arntzen, L. & Bukofzer, S. Susceptibility of Bacillus anthracis to eleven antimicrobial agents including novel fluoroquinolones and a ketolide. *J. Antimicrob. Chemother.* **52**, 297–299 (2003).
7. Steenbergen, J., Tanaka, S. K., Miller, L. L., Halasohoris, S. A. & Hershfield, J. R. In vitro and In vivo activity of omadacycline against two biothreat pathogens, bacillus anthracis and Yersinia pestis. *Am. Soc. Microbiol.* **61**, 1–9 (2017).
8. Veloo, A. C. M., Baas, W. H., Haan, F. J., Coco, J. & Rossen, J. W. Prevalence of antimicrobial resistance genes in Bacteroides spp. and Prevotella spp. Dutch clinical isolates. *Clin. Microbiol. Infect.* **25**, 1156.e9-1156.e13 (2019).
9. Sethi, S. *et al.* Emerging metronidazole resistance in Bacteroides spp. and its association with the nim gene: a study from North India. *J. Glob. Antimicrob. Resist.* **16**, 210–214 (2019).
10. Boyanova, L., Kolarov, R. & Mitov, I. Recent evolution of antibiotic resistance in the anaerobes as compared to previous decades. *Anaerobe* **31**, 4–10 (2015).
11. Nakano, V., e Silva, A. do N., Merino, V. R. C., Wexler, H. M. & Avila-Campos, M. J. Antimicrobial resistance and prevalence of resistance genes in intestinal Bacteroidales strains. *Clinics* **66**, 543– 547 (2011).
12. Wexler, H. M. Bacteroides: The good, the bad, and the nitty-gritty. *Clin. Microbiol. Rev.* **20**, 593– 621 (2007).
13. Cobo, F. *et al.* Clinical findings and antimicrobial susceptibility of anaerobic bacteria isolated in

bloodstream infections. *Antibiotics* **9**, 1–9 (2020).

1. Shimura, S. *et al.* Antimicrobial susceptibility surveillance of obligate anaerobic bacteria in the Kinki area. *J. Infect. Chemother.* **25**, 837–844 (2019).
2. Zeng, L. *et al.* Genetic characterization of a blaVIM-24-Carrying IncP-7ß plasmid p1160-VIM and a blaVIM-4-harboring integrative and conjugative element Tn6413 from clinical pseudomonas aeruginosa. *Front. Microbiol.* **10**, 1–9 (2019).
3. Schuetz, A. N. Antimicrobial resistance and susceptibility testing of anaerobic bacteria. *Clin. Infect. Dis.* **59**, 698–705 (2014).
4. Brook, I., Wexler, H. M. & Goldstein, E. J. C. Antianaerobic antimicrobials: Spectrum and susceptibility testing. *Clin. Microbiol. Rev.* **26**, 526–546 (2013).
5. Edwards, R. Resistance to β-lactam antibiotics in bacteroides spp. *J. Med. Microbiol.* **46**, 979–986 (1997).
6. Boente, R. F. *et al.* Detection of resistance genes and susceptibility patterns in Bacteroides and Parabacteroides strains. *Anaerobe* **16**, 190–194 (2010).
7. Wang, G., Zhao, G., Chao, X., Xie, L. & Wang, H. The characteristic of virulence, biofilm and antibiotic resistance of klebsiella pneumoniae. *Int. J. Environ. Res. Public Health* **17**, 1–17 (2020).
8. Maraki, S., Mavromanolaki, V. E., Stafylaki, D. & Kasimati, A. Antimicrobial susceptibility patterns of clinically significant Gram-positive anaerobic bacteria in a Greek tertiary-care hospital, 2017– 2019. *Anaerobe* **64**, (2020).
9. Piérard, D. *et al.* In vitro activity of ertapenem against anaerobes isolated from the respiratory tract. *Pathol. Biol.* **51**, 508–511 (2003).
10. Ogane, K. *et al.* Antimicrobial susceptibility and prevalence of resistance genes in Bacteroides fragilis isolated from blood culture bottles in two tertiary care hospitals in Japan. *Anaerobe* **64**, 102215 (2020).
11. Kierzkowska, M. *et al.* In vitro effect of clindamycin against Bacteroides and Parabacteroides isolates in Poland. *J. Glob. Antimicrob. Resist.* **13**, 49–52 (2018).
12. Vedantam, G. Antimicrobial resistance in Bacteroides spp.: occurrence and dissemination. *Future Microbiol.* **4**, 413–423 (2009).
13. Nakano, V. *et al.* Antimicrobial resistance and prevalence of resistance genes in intestinal Bacteroidales strains. *Bacteroidales strains. Clin.* **66**, 543–547 (2011).
14. Cordero-Laurent, E., Rodríguez, C., Rodríguez-Cavallini, E., Gamboa-Coronado, M. M. & Quesada- Gómez, C. Resistance of Bacteroides isolates recovered among clinical samples from a major Costa Rican hospital between 2000 and 2008 to ß-lactams, clindamycin, metronidazole, and chloramphenicol. *Rev. Esp. Quimioter.* **25**, 261–5 (2012).
15. Li, L. *et al.* High prevalence of macrolide-resistant bordetella pertussis and ptxP1 Genotype, Mainland China, 2014-2016. *Emerging Infectious Diseases* vol. 25 2205–2214 (2019).
16. Fu, P., Wang, C., Tian, H., Kang, Z. & Zeng, M. Bordetella pertussis Infection in Infants and Young Children in Shanghai, China, 2016-2017: Clinical Features, Genotype Variations of Antigenic

Genes and Macrolides Resistance. *Pediatr. Infect. Dis. J.* **38**, 370–376 (2019).

1. Barkoff, A.-M. & He, Q. Molecular Epidemiology of Bordetella pertussis. in *Pertussis Infection and Vaccines: Advances in Microbiology, Infectious Diseases and Public Health Volume 12* (eds. Fedele, G. & Ausiello, C. M.) 19–33 (Springer International Publishing, 2019). doi:10.1007/5584_2019_402.
2. Xu, Z. *et al.* Genomic epidemiology of erythromycin-resistant Bordetella pertussis in China.

*Emerg. Microbes Infect.* **8**, 461–470 (2019).

1. Baker, P. J. A Review of Antibiotic-Tolerant Persisters and Their Relevance to Posttreatment Lyme Disease Symptoms. *Am. J. Med.* **133**, 429–431 (2020).
2. Hunfeld, K. P. & Brade, V. Antimicrobial susceptibility of Borrelia burgdorferi sensu lato: What we know, what we don’t know, and what we need to know. *Wien. Klin. Wochenschr.* **118**, 659–668 (2006).
3. Terekhova, D., Sartakova, M. L., Wormser, G. P., Schwartz, I. & Cabello, F. C. Erythromycin resistance in Borrelia burgdorferi. *Antimicrob. Agents Chemother.* **46**, 3637–3640 (2002).
4. Jackson, C. R., Boylan, J., Frye, J. G. & Gherardini, F. C. Evidence of a conjugal erythromycin resistance element in the Lyme disease spirochete Borrelia burgdorferi. *Int. J. Antimicrob. Agents* **30**, 496–504 (2007).
5. Alamian, S., Dadar, M., Etemadi, A., Afshar, D. & Alamian, M. M. Antimicrobial susceptibility of Brucella spp. isolated from Iranian patients during 2016 to 2018. *Iran. J. Microbiol.* **11**, 363–367 (2019).
6. Ozhak-Baysan, B. *et al.* Evaluation of in vitro activities of tigecycline and various antibiotics against Brucella spp. *Polish J. Microbiol.* **59**, 55–60 (2010).
7. Torkaman Asadi, F., Hashemi, S. H., Yousef Alikhani, M., Moghimbeigi, A. & Naseri, Z. Clinical and diagnostic aspects of brucellosis and antimicrobial susceptibility of brucella isolates in Hamedan, Iran. *Jpn. J. Infect. Dis.* **70**, 235–238 (2017).
8. Abdel-Maksoud, M. *et al.* In vitro antibiotic susceptibility testing of Brucella isolates from Egypt between 1999 and 2007 and evidence of probable rifampin resistance. *Ann. Clin. Microbiol. Antimicrob.* **11**, 1 (2012).
9. Bolinger, H. & Kathariou, S. The current state of macrolide resistance in Campylobacter spp.: Trends and impacts of resistance mechanisms. *Appl. Environ. Microbiol.* **83**, 1–9 (2017).
10. Whelan, M. V. X. *et al.* Acquisition of fluoroquinolone resistance leads to increased biofilm formation and pathogenicity in Campylobacter jejuni. *Sci. Rep.* **9**, 1–13 (2019).
11. Abbasi, E., Abtahi, H., van Belkum, A. & Ghaznavi-Rad, E. Multidrug-resistant shigella infection in pediatric patients with diarrhea from central Iran. *Infect. Drug Resist.* **12**, 1535–1544 (2019).
12. Luangtongkum, T. *et al.* Antibiotic resistance in Campylobacter: Emergence, transmission and persistence. *Future Microbiol.* **4**, 189–200 (2009).
13. Sandoz, K. M. & Rockey, D. D. Antibiotic resistance in Chlamydiae. *Future Microbiol.* **5**, 1427–1442 (2010).
14. Borel, N., Leonard, C., Slade, J. & Schoborg, R. V. Chlamydial Antibiotic Resistance and Treatment Failure in Veterinary and Human Medicine. *Curr. Clin. Microbiol. Reports* **3**, 10–18 (2016).
15. Wang, Q. Y., Li, R. H., Zheng, L. Q. & Shang, X. H. Prevalence and antimicrobial susceptibility of Ureaplasma urealyticum and Mycoplasma hominis in female outpatients, 2009–2013. *J. Microbiol. Immunol. Infect.* **49**, 359–362 (2016).
16. Stamm, W. E. Potential for antimicrobial resistance in Chlamydia pneumoniae. *J. Infect. Dis.* **181**, 456–459 (2000).
17. Welsh, L. E., Gaydos, C. A. & Quinn, T. C. In vitro evaluation of activities of azithromycin, erythromycin, and tetracycline against Chlamydia trachomatis and Chlamydia pneumoniae. *Antimicrob. Agents Chemother.* **36**, 291–294 (1992).
18. Burillo, A. & Bouza, E. Chlamydophila pneumoniae. *Infect. Dis. Clin. North Am.* **24**, 61–71 (2010).
19. Binet, R. & Maurelli, A. T. Frequency of development and associated physiological cost of azithromycin resistance in Chlamydia psittaci 6BC and C. trachomatis L2. *Antimicrob. Agents Chemother.* **51**, 4267–4275 (2007).
20. Pathogen Safety Data Sheet - Infectious Substances (Chlamydia psittaci). *Canada Department of Health* [https://w](http://www.canada.ca/en/public-health/services/laboratory-biosafety-)ww.[canada.](http://www.canada.ca/en/public-health/services/laboratory-biosafety-)ca/e[n/publ](http://www.canada.ca/en/public-health/services/laboratory-biosafety-)i[c-health/services/laboratory-biosafety-](http://www.canada.ca/en/public-health/services/laboratory-biosafety-) biosecurity/pathogen-safety-data-sheets-risk-assessment/chlamydophila-psittaci.html (2011).
21. O’Brien, K. S. *et al.* Antimicrobial resistance following mass azithromycin distribution for trachoma: a systematic review. *Lancet Infect. Dis.* **19**, e14–e25 (2019).
22. AR, B., H, V., P, S., S, S. & A, M. Decreased susceptibility to azithromycin and doxycycline in clinical isolates of Chlamydia trachomatis obtained from recurrently infected female patients in India. *Chemotherapy* **56**, 371–377 (2010).
23. Doran, T. I. The role of Citrobacter in clinical disease of children: Review. *Clin. Infect. Dis.* **28**, 384– 394 (1999).
24. Pepperell, C., Kus, J. V., Gardam, M. A., Humar, A. & Burrows, L. L. Low-virulence Citrobacter species encode resistance to multiple antimicrobials. *Antimicrob. Agents Chemother.* **46**, 3555– 3560 (2002).
25. Liu, L. *et al.* Antimicrobial resistance and cytotoxicity of Citrobacter spp. in Maanshan Anhui Province, China. *Front. Microbiol.* **8**, 1–12 (2017).
26. Gür, D. *et al.* Comparative in vitro activity of plazomicin and older aminoglyosides against Enterobacterales isolates; prevalence of aminoglycoside modifying enzymes and 16S rRNA methyltransferases. *Diagn. Microbiol. Infect. Dis.* **97**, (2020).
27. Meini, S., Tascini, C., Cei, M., Sozio, E. & Rossolini, G. M. AmpC β-lactamase-producing Enterobacterales: what a clinician should know. *Infection* vol. 47 363–375 (2019).
28. Harris, P. N. A. & Ferguson, J. K. Antibiotic therapy for inducible AmpC β-lactamase-producing Gram-negative bacilli: What are the alternatives to carbapenems, quinolones and aminoglycosides? *Int. J. Antimicrob. Agents* **40**, 297–305 (2012).
29. Lalaoui, R. *et al.* Genomic characterization of Citrobacter freundii strains coproducing OXA-48 and VIM-1 carbapenemase enzymes isolated in leukemic patient in Spain. doi:10.1186/s13756-019-

0630-3.

1. BC, M., P, J. & BV, P. Antibiotic resistance in Citrobacter spp. isolated from urinary tract infection.

*Urol. Ann.* **5**, 312–313 (2013).

1. Lavigne, J. P., Defez, C., Bouziges, N., Mahamat, A. & Sotto, A. Clinical and molecular epidemiology of multidrug-resistant Citrobacter spp. infections in a French university hospital. *Eur. J. Clin. Microbiol. Infect. Dis.* **26**, 439–441 (2007).
2. Pfeifer, Y., Cullik, A. & Witte, W. Resistance to cephalosporins and carbapenems in Gram- negative bacterial pathogens. *Int. J. Med. Microbiol.* **300**, 371–379 (2010).
3. Mizrahi, A. *et al.* Infections caused by naturally AmpC-producing Enterobacteriaceae: Can we use third-generation cephalosporins? A narrative review. *Int. J. Antimicrob. Agents* **55**, 105834 (2020).
4. TA, L. *et al.* Finished Genome Sequence of the Highly Multidrug-Resistant Human Urine Isolate Citrobacter freundii Strain SL151. *Genome Announc.* **4**, (2016).
5. M, T. *et al.* Changing Trends in Prevalence and Antibiotics Resistance of Uropathogens in Patients Attending the Gondar University Hospital, Northwest Ethiopia. *Int. J. Bacteriol.* **2014**, 1–7 (2014).
6. Pfaller, M. A., Huband, M. D., Shortridge, D. & Flamm, R. K. Surveillance of omadacycline activity tested against clinical isolates from the United States and Europe: Report from the SENTRY antimicrobial surveillance program, 2016 to 2018. *Antimicrob. Agents Chemother.* **64**, 1–21

(2020).

1. Fischer, J. *et al.* Low-level antimicrobial resistance of Enterobacteriaceae isolated from the nares of pig-exposed persons. *Epidemiol. Infect.* **144**, 686–690 (2016).
2. Spigaglia, P., Mastrantonio, P. & Barbanti, F. Antibiotic resistances of Clostridium difficile. *Adv. Exp. Med. Biol.* **1050**, 137–159 (2018).
3. Khademi, F. & Sahebkar, A. The prevalence of antibiotic-resistant Clostridium species in Iran: a meta-analysis. *Pathog. Glob. Health* **113**, 58–66 (2019).
4. US Department of Health and Human Services & CDC. Antibiotic Resistance Threats in the United States. *Centers Dis. Control Prev.* 1–113 (2013).
5. Barkin, J. A., Sussman, D. A., Fifadara, N. & Barkin, J. S. Clostridium difficile Infection and Patient- Specific Antimicrobial Resistance Testing Reveals a High Metronidazole Resistance Rate. *Dig. Dis. Sci.* **62**, 1035–1042 (2017).
6. Fayez, M. *et al.* Genotyping and antimicrobial susceptibility of Clostridium perfringens isolated from dromedary camels, pastures and herders. *Comp. Immunol. Microbiol. Infect. Dis.* **70**, 101460 (2020).
7. Yadav, A. K. *et al.* Association of Antimicrobial Susceptibility and Treatment Outcome in Acne Vulgaris Patients: A Pilot Study. *J. Lab. Physicians* **12**, 233–238 (2020).
8. Sárvári, K. P. & Schoblocher, D. The antibiotic susceptibility pattern of gas gangrene-forming Clostridium spp. clinical isolates from South-Eastern Hungary. *Infect. Dis. (Auckl).* **52**, 196–201 (2020).
9. MT, A. *et al.* Antibiotic Sensitivity of Clostridium perfringens Isolated From Faeces in Tabriz, Iran.

*Jundishapur J. Microbiol.* **8**, (2015).

1. U, T., W, M. & L, S. Antimicrobial resistance among Clostridium perfringens isolated from various sources in Thailand. *Southeast Asian J. Trop. Med. Public Health* **36**, 954–961 (2005).
2. Adams, V., Han, X., Lyras, D. & Rood, J. I. Antibiotic resistance plasmids and mobile genetic elements of Clostridium perfringens. *Plasmid* **99**, 32–39 (2018).
3. JP, Y. *et al.* Molecular characterization and antimicrobial resistance profile of Clostridium perfringens type A isolates from humans, animals, fish and their environment. *Anaerobe* **47**, 120– 124 (2017).
4. Pathogen Safety Data Sheets: Infectious Substances – Clostridium spp. *Canada Department of Health* [https://w](http://www.canada.ca/en/public-health/services/laboratory-biosafety-)ww.[canada.](http://www.canada.ca/en/public-health/services/laboratory-biosafety-)ca/e[n/publ](http://www.canada.ca/en/public-health/services/laboratory-biosafety-)i[c-health/services/laboratory-biosafety-](http://www.canada.ca/en/public-health/services/laboratory-biosafety-) biosecurity/pathogen-safety-data-sheets-risk-assessment/clostridium.html.
5. Wang, L. min, Qiao, X. liang, Ai, L., Zhai, J. jing & Wang, X. xia. Isolation of antimicrobial resistant bacteria in upper respiratory tract infections of patients. *3 Biotech* **6**, 1–7 (2016).
6. Alexander, C. J., Citron, D. M., Brazier, J. S. & Goldstein, E. J. C. Identification and antimicrobial resistance patterns of clinical isolates of Clostridium clostridioforme, Clostridium innocuum, and Clostridium ramosum compared with those of clinical isolates of Clostridium perfringens. *J. Clin. Microbiol.* **33**, 3209–3215 (1995).
7. Hanif, H. *et al.* Isolation and antibiogram of clostridium tetani from clinically diagnosed tetanus patients. *Am. J. Trop. Med. Hyg.* **93**, 752–756 (2015).
8. Hennart, M. *et al.* Population genomics and antimicrobial resistance in Corynebacterium diphtheriae. *Genome Med.* **12**, 1–18 (2020).
9. Paveenkittiporn, W., Sripakdee, S., Koobkratok, O., Sangkitporn, S. & Kerdsin, A. Molecular epidemiology and antimicrobial susceptibility of outbreak-associated Corynebacterium diphtheriae in Thailand, 2012. *Infect. Genet. Evol.* **75**, 104007 (2019).
10. Husada, D. *et al.* First-line antibiotic susceptibility pattern of toxigenic Corynebacterium diphtheriae in Indonesia. *BMC Infect. Dis.* **19**, 1–11 (2019).
11. Davin-Regli, A., Lavigne, J. P. & Pagès, J. M. Enterobacter spp.: update on taxonomy, clinical aspects, and emerging antimicrobial resistance. *Clin. Microbiol. Rev.* **32**, 1–32 (2019).
12. Davin-Regli, A. & Pagès, J. M. Enterobacter aerogenes and Enterobacter cloacae; Versatile bacterial pathogens confronting antibiotic treatment. *Front. Microbiol.* **6**, 1–10 (2015).
13. Ngalani, O. J. T., Mbaveng, A. T., Marbou, W. J. T., Ngai, R. Y. & Kuete, V. Antibiotic Resistance of Enteric Bacteria in HIV-Infected Patients at the Banka Ad-Lucem Hospital, West Region of Cameroon. *Can. J. Infect. Dis. Med. Microbiol.* **2019**, (2019).
14. Lavigne, J. P. *et al.* Membrane permeability, a pivotal function involved in antibiotic resistance and virulence in Enterobacter aerogenes clinical isolates. *Clin. Microbiol. Infect.* **18**, 539–545 (2012).
15. Thiolas, A., Bornet, C., Davin-Régli, A., Pagès, J. M. & Bollet, C. Resistance to imipenem, cefepime, and cefpirome associated with mutation in Omp36 osmoporin of Enterobacter aerogenes.

*Biochem. Biophys. Res. Commun.* **317**, 851–856 (2004).

1. Eugene Sanders, W. E. & Sanders, C. C. Enterobacter spp.: Pathogens poised to flourish at the turn of the century. *Clin. Microbiol. Rev.* **10**, 220–241 (1997).
2. Mishra, M. P., Sarangi, R. & Padhy, R. N. Prevalence of multidrug resistant uropathogenic bacteria in pediatric patients of a tertiary care hospital in eastern India. *J. Infect. Public Health* **9**, 308–314 (2016).
3. G, J.-G. *et al.* Susceptibility evolution to antibiotics of Enterobacter cloacae, Morganella morganii, Klebsiella aerogenes and Citrobacter freundii involved in urinary tract infections: an 11-year epidemiological surveillance study. *Enfermedades Infecc. y Microbiol. Clin. (English ed.)* **38**, 166– 169 (2020).
4. Akhtar, N., Alqurashi, A. & Twibah, M. In vitro ciprofloxacin resistance profiles among gram- negative bacteria isolated from clinical specimens in a teaching hospital. *J. Pakistan Med. Assoc.* (2010).
5. Moussa, A. A., Nordin, A. F. M., Hamat, R. A. & Jasni, A. S. High level aminoglycoside resistance and distribution of the resistance genes in Enterococcus faecalis and Enterococcus faecium from teaching hospital in Malaysia. *Infect. Drug Resist.* **12**, 3269–3274 (2019).
6. Van Tyne, D. & Gilmore, M. S. Friend turned foe: Evolution of enterococcal virulence and antibiotic resistance. *Annu. Rev. Microbiol.* **68**, 337–356 (2014).
7. Ono, S., Muratani, T. & Matsumoto, T. Mechanisms of resistance to imipenem and ampicillin in Enterococcus faecalis. *Antimicrob. Agents Chemother.* **49**, 2954–2958 (2005).
8. Takesue, Y. *et al.* Antimicrobial susceptibility of common pathogens isolated from postoperative intra-abdominal infections in Japan. *J. Infect. Chemother.* **24**, 330–340 (2018).
9. Rice, L. B. *et al.* crossm ␤ -Lactam Susceptibility in Enterococcus faecalis. **9**, 1–12 (2018).
10. Khani, M., Fatollahzade, M., Pajavand, H., Bakhtiari, S. & Abiri, R. Increasing prevalence of aminoglycoside-resistant enterococcus faecalis isolates due to the aac(6’)-aph(2’’”) Gene: A therapeutic problem in Kermanshah, Iran. *Jundishapur J. Microbiol.* **9**, (2016).
11. SMJ, S. *et al.* First detection of efrAB, an ABC multidrug efflux pump in Enterococcus faecalis in Tehran, Iran. *Acta Microbiol. Immunol. Hung.* **66**, 57–68 (2019).
12. MAM, E., HM, A. & RM, K. Prevalence of Multidrug-Resistant Enterococcus faecalis in Hospital- Acquired Surgical Wound Infections and Bacteremia: Concomitant Analysis of Antimicrobial Resistance Genes. *Infect. Dis. (Auckl).* **12**, 117863371988292 (2019).
13. Kot, B. Antibiotic Resistance among Uropathogenic Escherichia coli. *Polish J. Microbiol.* **68**, 403– 415 (2019).
14. Lee, J. Y., Hong, Y. K., Lee, H. & Ko, K. S. High prevalence of non-clonal imipenem-nonsusceptible Enterobacter spp. isolates in Korea and their association with porin down-regulation. *Diagn. Microbiol. Infect. Dis.* **87**, 53–59 (2017).
15. Katongole, P., Nalubega, F., Florence, N. C., Asiimwe, B. & Andia, I. Biofilm formation, antimicrobial susceptibility and virulence genes of Uropathogenic Escherichia coli isolated from clinical isolates in Uganda. *BMC Infect. Dis.* **20**, 1–6 (2020).
16. Kresken, M. *et al.* Comparative in vitro activity of oral antimicrobial agents against Enterobacteriaceae from patients with community-acquired urinary tract infections in three European countries. *Clin. Microbiol. Infect.* **22**, 63.e1-63.e5 (2016).
17. Yangzom, T., Tsering, D. C., Kar, S. & Kapil, J. Antimicrobial Susceptibility Trends among Pathogens Isolated from Blood: A 6-Year Retrospective Study from a Tertiary Care Hospital in East Sikkim, India. *J. Lab. Physicians* **12**, 03–09 (2020).
18. Daoud, Z. *et al.* Resistance and clonality in Escherichia coli and Klebsiella spp. and relationship with antibiotic consumption in major Lebanese hospitals. *J. Glob. Antimicrob. Resist.* **11**, 45–51 (2017).
19. Zhang, S. X. *et al.* Antibiotic resistance and molecular characterization of diarrheagenic Escherichia coli and non-typhoidal Salmonella strains isolated from infections in Southwest China. *Infect. Dis. Poverty* **7**, 1–11 (2018).
20. Bae, I. G. & Stone, G. G. Activity of ceftaroline against pathogens associated with community- acquired pneumonia collected as part of the AWARE surveillance program, 2015–2016. *Diagn. Microbiol. Infect. Dis.* **95**, 114843 (2019).
21. Zhanel, G. G. *et al.* 42936 pathogens from Canadian hospitals: 10 years of results (2007-16) from the CANWARD surveillance study. *J. Antimicrob. Chemother.* **74**, iv5–iv21 (2019).
22. Denisuik, A. J. *et al.* Antimicrobial-resistant pathogens in Canadian ICUs: Results of the CANWARD 2007 to 2016 study. *J. Antimicrob. Chemother.* **74**, 645–653 (2019).
23. Stapleton, P. J. *et al.* Antibiotic resistance patterns of Escherichia coli urinary isolates and comparison with antibiotic consumption data over 10 years, 2005–2014. *Ir. J. Med. Sci.* **186**, 733– 741 (2017).
24. Caspar, Y., Hennebique, A. & Maurin, M. Antibiotic susceptibility of Francisella tularensis subsp. holarctica strains isolated from tularaemia patients in France between 2006 and 2016. *J. Antimicrob. Chemother.* **73**, 687–691 (2018).
25. Georgi, E., Schacht, E., Scholz, H. C. & Splettstoesser, W. D. Standardized broth microdilution antimicrobial susceptibility testing of Francisella tularensis subsp. holarctica strains from Europe and rare Francisella species. *J. Antimicrob. Chemother.* **67**, 2429–2433 (2012).
26. Yeşilyurt, M. *et al.* Antimicrobial susceptibilities of Francisella tularensis subsp. holarctica strains isolated from humans in the Central Anatolia region of Turkey. *J. Antimicrob. Chemother.* **66**, 2588–2592 (2011).
27. Siebert, C. *et al.* Francisella tularensis: FupA mutation contributes to fluoroquinolone resistance by increasing vesicle secretion and biofilm formation. *Emerg. Microbes Infect.* **8**, 808–822 (2019).
28. Sutera, V., Hoarau, G., Renesto, P., Caspar, Y. & Maurin, M. In vitro and in vivo evaluation of fluoroquinolone resistance associated with DNA gyrase mutations in Francisella tularensis, including in tularaemia patients with treatment failure. *Int. J. Antimicrob. Agents* **50**, 377–383 (2017).
29. Origgi, F. C., Frey, J. & Pilo, P. Characterisation of a new group of Francisella tularensis subsp. Holarctica in Switzerland with altered antimicrobial susceptibilities, 1996 to 2013. *Eurosurveillance* **19**, 1–10 (2014).
30. Shilnikova, I. I. & Dmitrieva, N. V. Evaluation of antibiotic susceptibility of Bacteroides, Prevotella and Fusobacterium species isolated from patients of the N. N. Blokhin Cancer Research Center, Moscow, Russia. *Anaerobe* **31**, 15–18 (2015).
31. Fujita, K. *et al.* Antimicrobial susceptibilities of clinical isolates of the anaerobic bacteria which can cause aspiration pneumonia. *Anaerobe* **57**, 86–89 (2019).
32. Maraki, S., Mavromanolaki, V. E., Stafylaki, D. & Kasimati, A. Surveillance of antimicrobial resistance in recent clinical isolates of Gram-negative anaerobic bacteria in a Greek University Hospital. *Anaerobe* **62**, (2020).
33. Shilnikova, I. I. & Dmitrieva, N. V. Evaluation of Antibiotic Susceptibility of Gram-Positive Anaerobic Cocci Isolated from Cancer Patients of the N. N. Blokhin Russian Cancer Research Center. *J. Pathog.* **2015**, 1–5 (2015).
34. Wang, F. D., Liao, C. H., Lin, Y. T., Sheng, W. H. & Hsueh, P. R. Trends in the susceptibility of commonly encountered clinically significant anaerobes and susceptibilities of blood isolates of anaerobes to 16 antimicrobial agents, including fidaxomicin and rifaximin, 2008–2012, northern Taiwan. *Eur. J. Clin. Microbiol. Infect. Dis.* **33**, 2041–2052 (2014).
35. KE, A. *et al.* Multicenter survey of the changing in vitro antimicrobial susceptibilities of clinical isolates of Bacteroides fragilis group, Prevotella, Fusobacterium, Porphyromonas, and Peptostreptococcus species. *Antimicrob. Agents Chemother.* **45**, 1238–1243 (2001).
36. J, P. *et al.* Epidemiological characteristics of infections caused by Bacteroides, Prevotella and Fusobacterium species: a prospective observational study. *Anaerobe* **17**, 113–117 (2011).
37. Goldstein, E. J. C., Citron, D. M., Cherubin, C. E. & Hillier, S. L. Comparative susceptibility of the bacteroides fragilis group species and other anaerobic bacteria to meropenem, imipenem, piperacillin, cefoxitin, ampicillin/sulbactam, clindamycin and metronidazole. *J. Antimicrob. Chemother.* **31**, 363–372 (1993).
38. Nagaraja, P. Antibiotic resistance of Gardnerella vaginalis in recurrent bacterial vaginosis. *Indian*

*J. Med. Microbiol.* **26**, 155–157 (2008).

1. de Souza, D. M. K. *et al.* Antimicrobial susceptibility and vaginolysin in Gardnerella vaginalis from healthy and bacterial vaginosis diagnosed women. *J. Infect. Dev. Ctries.* **10**, 913–919 (2016).
2. Murphy, E. C. & Frick, I. M. Gram-positive anaerobic cocci - commensals and opportunistic pathogens. *FEMS Microbiol. Rev.* **37**, 520–553 (2013).
3. Badri, M., Nilson, B., Ragnarsson, S., Senneby, E. & Rasmussen, M. Clinical and microbiological features of bacteraemia with Gram-positive anaerobic cocci: a population-based retrospective study. *Clin. Microbiol. Infect.* **25**, 760.e1-760.e6 (2019).
4. Byun, J. H., Kim, M., Lee, Y., Lee, K. & Chong, Y. Antimicrobial susceptibility patterns of anaerobic bacterial clinical isolates from 2014 to 2016, including recently named or renamed species. *Ann. Lab. Med.* **39**, 190–199 (2019).
5. Murdoch, D. A. Gram-Positive Anaerobic Cocci. **11**, 81–120 (1998).
6. Kuriyama, T., Karasawa, T., Nakagawa, K., Yamamoto, E. & Nakamura, S. Bacteriology and antimicrobial susceptibility of gram-positive cocci isolated from pus specimens of orofacial

odontogenic infections. *Oral Microbiol. Immunol.* **17**, 132–135 (2002).

1. Alauzet, C., Lozniewski, A. & Marchandin, H. Metronidazole resistance and nim genes in anaerobes: A review. *Anaerobe* **55**, 40–53 (2019).
2. Brazier, J. S., Hall, V., Morris, T. E., Gal, M. & Duerden, B. I. Antibiotic susceptibilities of Gram- positive anaerobic cocci: Results of a sentinel study in England and Wales. *J. Antimicrob. Chemother.* **52**, 224–228 (2003).
3. Ubukata, K. *et al.* Genetic characteristics and antibiotic resistance of Haemophilus influenzae isolates from pediatric patients with acute otitis media after introduction of 13-valent pneumococcal conjugate vaccine in Japan. *J. Infect. Chemother.* **25**, 720–726 (2019).
4. Guitor, A. K. & Wright, G. D. Antimicrobial Resistance and Respiratory Infections. *Chest* **154**, 1202–1212 (2018).
5. Pfaller, M. A., Farrell, D. J., Sader, H. S. & Jones, R. N. AWARE ceftaroline surveillance program (2008-2010): Trends in resistance patterns among streptococcus pneumoniae, Haemophilus influenzae, and Moraxella catarrhalis in the United States. *Clin. Infect. Dis.* **55**, 187–193 (2012).
6. Heinz, E. The return of pfeiffer’s bacillus: Rising incidence of ampicillin resistance in haemophilus influenzae. *Microb. Genomics* **4**, (2018).
7. Li, J. P. *et al.* Epidemiological Features and Antibiotic Resistance Patterns of Haemophilus influenzae Originating from Respiratory Tract and Vaginal Specimens in Pediatric Patients. *J. Pediatr. Adolesc. Gynecol.* **30**, 626–631 (2017).
8. Wen, S., Feng, D., Chen, D., Yang, L. & Xu, Z. Molecular epidemiology and evolution of Haemophilus influenzae. *Infect. Genet. Evol.* **80**, (2020).
9. Mohd-Zain, Z., Kamsani, N. H., Ismail, I. S. & Ahmad, N. Antibiotic susceptibility profile of Haemophilus influenzae and transfer of co-trimoxazole resistance determinants. *Trop. Biomed.* **29**, 372–380 (2012).
10. S, B. *et al.* Antimicrobial resistance in Haemophilus influenzae respiratory tract isolates in Korea: results of a nationwide acute respiratory infections surveillance. *Antimicrob. Agents Chemother.* **54**, 65–71 (2010).
11. Wang, H. J. *et al.* Antibiotic Resistance Profiles of Haemophilus influenzae Isolates from Children in 2016: A Multicenter Study in China. *Can. J. Infect. Dis. Med. Microbiol.* **2019**, (2019).
12. C, T., P, P. & S, S. HAEMOPHILUS INFLUENZAE FROM PATIENTS AT THE LARGEST UNIVERSITY

TERTIARY CARE CENTER, THAILAND 2012 - 2015. *Southeast Asian J. Trop. Med. Public Health* **48**, (2017).

1. M, K. *et al.* β-Lactam resistance among Haemophilus influenzae isolates in Poland. *J. Glob. Antimicrob. Resist.* **11**, 161–166 (2017).
2. Torumkuney, D. *et al.* Results from the Survey of Antibiotic Resistance (SOAR) 2015-17 in Turkey: Data based on CLSI, EUCAST (dose-specific) and pharmacokinetic/pharmacodynamic (PK/PD) breakpoints. *J. Antimicrob. Chemother.* **75**, I88–I99 (2020).
3. Sader, H. S., Flamm, R. K., Streit, J. M., Carvalhaes, C. G. & Mendes, R. E. Antimicrobial activity of ceftaroline and comparator agents tested against organisms isolated from patients with

community-acquired bacterial pneumonia in Europe, Asia, and Latin America. *Int. J. Infect. Dis.*

**77**, 82–86 (2018).

1. Cherkaoui, A. *et al.* Molecular characterization of fluoroquinolones, macrolides, and imipenem resistance in Haemophilus influenzae: analysis of the mutations in QRDRs and assessment of the extent of the AcrAB-TolC-mediated resistance. *Eur. J. Clin. Microbiol. Infect. Dis.* **37**, 2201–2210 (2018).
2. Karlowsky, J. A. *et al.* In vitro activity of Ceftaroline against bacterial pathogens isolated from patients with skin and soft tissue and respiratory tract infections in African and Middle Eastern countries: AWARE global surveillance program 2012–2014. *Diagn. Microbiol. Infect. Dis.* **86**, 194– 199 (2016).
3. Hoban, D., Biedenbach, D., Sahm, D., Reiszner, E. & Iaconis, J. Activity of ceftaroline and comparators against pathogens isolated from skin and soft tissue infections in Latin America - results of AWARE surveillance 2012. *Brazilian J. Infect. Dis.* **19**, 596–603 (2015).
4. Karlowsky, J. A. *et al.* In vitro activity of ceftaroline against bacterial pathogens isolated from skin and soft tissue infections in Europe, Russia and Turkey in 2012: Results from the Assessing Worldwide Antimicrobial Resistance Evaluation (AWARE) surveillance programme. *J. Antimicrob. Chemother.* **71**, 162–169 (2016).
5. Durdu, B. *et al.* Risk factors affecting patterns of antibiotic resistance and treatment efficacy in extreme drug resistance in intensive care unit-acquired klebsiella pneumoniae infections: A 5- year analysis. *Med. Sci. Monit.* **25**, 174–183 (2019).
6. Juan, C. H., Chuang, C., Chen, C. H., Li, L. & Lin, Y. T. Clinical characteristics, antimicrobial resistance and capsular types of community-acquired, healthcare-associated, and nosocomial Klebsiella pneumoniae bacteremia. *Antimicrob. Resist. Infect. Control* **8**, 1–9 (2019).
7. Piperaki, E. T., Syrogiannopoulos, G. A., Tzouvelekis, L. S. & Daikos, G. L. Klebsiella pneumoniae: Virulence, Biofilm and Antimicrobial Resistance. *Pediatr. Infect. Dis. J.* **36**, 1002–1005 (2017).
8. de Paula, A., Oliva, G., Barraquer, R. I. & de la Paz, M. F. Prevalence and antibiotic susceptibility of bacteria isolated in patients affected with blepharitis in a tertiary eye centre in Spain. *Eur. J. Ophthalmol.* **30**, 991–997 (2020).
9. Liu, B. *et al.* Antimicrobial resistance and risk factors for mortality of pneumonia caused by klebsiella pneumoniae among diabetics: A retrospective study conducted in Shanghai, China. *Infect. Drug Resist.* **12**, 1089–1098 (2019).
10. Bui, T. & Preuss, C. V. *Cephalosporins*. *NCBI Bookshelf* (StatPearls Publishing LLC, 2021).
11. HS, L., YX, L., JJ, L., CS, L. & C, C. Antimicrobial consumption and resistance in five Gram-negative bacterial species in a hospital from 2003 to 2011. *J. Microbiol. Immunol. Infect.* **48**, 647–654 (2015).
12. WP, L. *et al.* The Antimicrobial Susceptibility of Klebsiella pneumoniae from Community Settings in Taiwan, a Trend Analysis. *Sci. Rep.* **6**, (2016).
13. SS, G. *et al.* Phenotypic and genotypic detection of ESBL mediated cephalosporin resistance in Klebsiella pneumoniae: emergence of high resistance against cefepime, the fourth generation cephalosporin. *J. Infect.* **53**, 279–288 (2006).
14. Ginn, A. N. *et al.* Limited diversity in the gene pool allows prediction of third-generation cephalosporin and aminoglycoside resistance in Escherichia coli and Klebsiella pneumoniae. *Int. J. Antimicrob. Agents* **42**, 19–26 (2013).
15. Liu, C. *et al.* Antimicrobial resistance in South Korea: A report from the Korean global antimicrobial resistance surveillance system (Kor-GLASS) for 2017. *J. Infect. Chemother.* **25**, 845– 859 (2019).
16. HS, S., RK, F., RE, M., DJ, F. & RN, J. Antimicrobial Activities of Ceftaroline and Comparator Agents against Bacterial Organisms Causing Bacteremia in Patients with Skin and Skin Structure Infections in U.S. Medical Centers, 2008 to 2014. *Antimicrob. Agents Chemother.* **60**, 2558–2563

(2016).

1. Flamm, R. K., Sader, H. S., Farrell, D. J. & Jones, R. N. Summary of ceftaroline activity against pathogens in the United States, 2010: Report from the Assessing Worldwide Antimicrobial Resistance Evaluation (AWARE) surveillance program. *Antimicrob. Agents Chemother.* **56**, 2933– 2940 (2012).
2. Wang, Y. *et al.* Higher prevalence of multi-antimicrobial resistant bacteroides spp. Strains isolated at a tertiary teaching hospital in China. *Infect. Drug Resist.* **13**, 1537–1546 (2020).
3. Navon-Venezia, S., Kondratyeva, K. & Carattoli, A. Klebsiella pneumoniae: A major worldwide source and shuttle for antibiotic resistance. *FEMS Microbiol. Rev.* **41**, 252–275 (2017).
4. Haldorsen, B. C., Simonsen, G. S., Sundsfjord, A. & Samuelsen, Ø. Increased prevalence of aminoglycoside resistance in clinical isolates of Escherichia coli and Klebsiella spp. in Norway is associated with the acquisition of AAC(3)-II and AAC(6’)-Ib. *Diagn. Microbiol. Infect. Dis.* **78**, 66– 69 (2014).
5. Sekar, R., Mythreyee, M., Srivani, S. & Amudhan, M. Prevalence of antimicrobial resistance in Escherichia coli and Klebsiella spp. in rural South India. *J. Glob. Antimicrob. Resist.* **5**, 80–85 (2016).
6. Paul, M., Bhatia, M., Rekha, U. S., Omar, B. J. & Gupta, P. Microbiological Profile of Blood Stream Infections in Febrile Neutropenic Patients at a Tertiary Care Teaching Hospital in Rishikesh, Uttarakhand. *J. Lab. Physicians* **12**, 147–153 (2020).
7. Pappa, O. *et al.* Antibiotic resistance of legionella pneumophila in clinical and water isolates—a systematic review. *Int. J. Environ. Res. Public Health* **17**, 1–18 (2020).
8. Natås, O. B. *et al.* Susceptibility of Legionella pneumophila to antimicrobial agents and the presence of the efflux pump LpeAB. *J. Antimicrob. Chemother.* **74**, 1545–1550 (2019).
9. Miyashita, N. *et al.* In vitro activity of various antibiotics against clinical strains of Legionella species isolated in Japan. *J. Infect. Chemother.* **24**, 325–329 (2018).
10. Miraglia, F. *et al.* Molecular characterization, serotyping, and antibiotic susceptibility profile of Leptospira interrogans serovar Copenhageni isolates from Brazil. *Diagn. Microbiol. Infect. Dis.* **77**, 195–199 (2013).
11. Moreno, L. Z. *et al.* Profiling of Leptospira interrogans, L. santarosai, L. meyeri and L. borgpetersenii by SE-AFLP, PFGE and susceptibility testing-a continuous attempt at species and serovar differentiation. *Emerg. Microbes Infect.* **5**, (2016).
12. Shi, W. *et al.* β-Lactamase production and antibiotic susceptibility pattern of Moraxella catarrhalis isolates collected from two county hospitals in China. *BMC Microbiol.* **18**, 1–6 (2018).
13. Król-Turmińska, K., Olender, A. & Bogut, A. Tetracycline resistance in Moraxella catarrhalis clinical strains isolated in Poland. *New Microbiol.* **43**, 103–106 (2020).
14. Hoban, D. J., Doern, G. V., Fluit, A. C., Roussel-Delvallez, M. & Jones, R. N. Worldwide prevalence of antimicrobial resistance in Streptococcus pneumoniae, Haemophilus influenzae, and Moraxella catarrhalis in the SENTRY Antimicrobial Surveillance Program, 1997-1999. *Clin. Infect. Dis.* **32**, (2001).
15. Baquero, F., F. Lanza, V., Duval, M. & Coque, T. M. Ecogenetics of antibiotic resistance in Listeria monocytogenes. *Mol. Microbiol.* **113**, 570–579 (2020).
16. Moreno, L. Z. *et al.* Characterization of antibiotic resistance in Listeria spp. isolated from slaughterhouse environments, pork and human infections. *J. Infect. Dev. Ctries.* **8**, 416–423 (2014).
17. Safdar, A. & Armstrong, D. Antimicrobial activities against 84 Listeria monocytogenes isolates from patients with systemic listeriosis at a Comprehensive Cancer Center (1955-1997). *J. Clin. Microbiol.* **41**, 483–485 (2003).
18. Hu, F. *et al.* Results from the Survey of Antibiotic Resistance (SOAR) 2009-11 and 2013-14 in China. *J. Antimicrob. Chemother.* **71**, i33–i43 (2016).
19. SB, S., Z, A., SA, A. & S, S. Prevalence and resistance pattern of Moraxella catarrhalis in community-acquired lower respiratory tract infections. *Infect. Drug Resist.* **8**, 263–267 (2015).
20. S, R., G, K., RN, S. & M, D. Moraxella catarrhalis: A Cause of Concern with Emerging Resistance and Presence of BRO Beta-Lactamase Gene-Report from a Tertiary Care Hospital in South India. *Int. J. Microbiol.* **2020**, (2020).
21. Du, Y. *et al.* Multilocus sequence typing-based analysis of Moraxella catarrhalis population structure reveals clonal spreading of drug-resistant strains isolated from childhood pneumonia. *Infect. Genet. Evol.* **56**, 117–124 (2017).
22. Pereyre, S., Goret, J. & Bébéar, C. Mycoplasma pneumoniae: Current knowledge on macrolide resistance and treatment. *Front. Microbiol.* **7**, 1–11 (2016).
23. Guo, D. X. *et al.* Epidemiology and mechanism of drug resistance of mycoplasma pneumoniae in Beijing, China: A multicenter study. *Bosn. J. Basic Med. Sci.* **19**, 288–296 (2019).
24. Yang, F., Yan, J., Zhang, J. & van der Veen, S. Evaluation of alternative antibiotics for susceptibility of gonococcal isolates from China. *Int. J. Antimicrob. Agents* **55**, (2020).
25. Horn, N. N. *et al.* Antimicrobial susceptibility and molecular epidemiology of Neisseria gonorrhoeae in Germany. *Int. J. Med. Microbiol.* **304**, 586–591 (2014).
26. D.A., W. *et al.* Trends and Risk Factors for Antimicrobial-Resistant Neisseria gonorrhoeae, Melbourne, Australia, 2007 to 2018. *Antimicrob. Agents Chemother.* **63**, Arte Number: e01221-

19. ate of Pubaton: 2019 (2019).

1. McAuliffe, G. N. *et al.* Keeping track of antimicrobial resistance for neisseria gonorrhoeae in Auckland, New Zealand: Past, present and future considerations. *N. Z. Med. J.* **131**, 71–77 (2018).
2. T, W. *et al.* Antimicrobial resistance in Neisseria gonorrhoeae: Global surveillance and a call for international collaborative action. *PLoS Med.* **14**, (2017).
3. M, U. & WM, S. Antibiotic resistance in Neisseria gonorrhoeae: origin, evolution, and lessons learned for the future. *Ann. N. Y. Acad. Sci.* **1230**, (2011).
4. Sáez-Nieto, J. A. *et al.* Epidemiology and Molecular Basis of Penicillin-Resistant Neisseria meningitidis in Spain: A 5-Year History (1985-1989)s. *Clin. Infect. Dis.* **14**, 394–402 (1992).
5. Oppenheim, B. A. Antibiotic resistance in Neisseria meningitidis. *Clin. Infect. Dis.* **24**, 98–101 (1997).
6. Vacca, P. *et al.* Neisseria meningitidis antimicrobial resistance in Italy, 2006 to 2016. *Antimicrob. Agents Chemother.* **62**, 1–6 (2018).
7. Oncel, E. K. *et al.* Surveillance of penicillin resistance of Neisseria meningitidis strains from invasive infections between 2013 and 2018 in Turkey. *https://doi.org/10.1080/1120009X.2020.1721176* **32**, 213–216 (2020).
8. Alemayehu, T., Mekasha, A. & Abebe, T. Nasal carriage rate and antibiotic susceptibility pattern of Neisseria meningitidis in healthy Ethiopian children and adolescents: A cross-sectional study. *PLoS One* **12**, 1–11 (2017).
9. L, A., L, de L. F. & JA, V. Antibiotic susceptibility patterns of Neisseria meningitidis isolates from patients and asymptomatic carriers. *Antimicrob. Agents Chemother.* **44**, 1705–1707 (2000).
10. LA, M. *et al.* Detection of Ciprofloxacin-Resistant, β-Lactamase-Producing Neisseria meningitidis Serogroup Y Isolates - United States, 2019-2020. *MMWR. Morb. Mortal. Wkly. Rep.* **69**, 735–739 (2020).
11. Tefera, Z., Mekonnen, F., Tiruneh, M. & Belachew, T. Carriage rate of Neisseria meningitidis, antibiotic susceptibility pattern and associated risk factors among primary school children in Gondar town, Northwest Ethiopia. *BMC Infect. Dis.* **20**, 1–10 (2020).
12. Lee, H., Seo, Y., Kim, K. H., Lee, K. & Choe, K. W. Prevalence and serogroup changes of Neisseria meningitidis in South Korea. *Sci. Rep.* **8**, 2010–2016 (2018).
13. van de Beek, D. *et al.* Meropenem susceptibility of Neisseria meningitidis and Streptococcus. 895–897 (1997).
14. Gorla, M. C., Pinhata, J. M. W., Dias, U. J., de Moraes, C. & Lemos, A. P. Surveillance of antimicrobial resistance in neisseria meningitidis strains isolated from invasive cases in Brazil from 2009 to 2016. *J. Med. Microbiol.* **67**, 750–756 (2018).
15. Jorgensen, J. H., Crawford, S. A. & Fiebelkorn, K. R. Susceptibility of Neisseria meningitidis to 16 antimicrobial agents and characterization of resistance mechanisms affecting some agents. *J. Clin. Microbiol.* **43**, 3162–3171 (2005).
16. Zouheir, Y., Atany, T. & Boudebouch, N. Emergence and spread of resistant N. meningitidis implicated in invasive meningococcal diseases during the past decade (2008–2017). *J. Antibiot. (Tokyo).* **72**, 185–188 (2019).
17. Huang, L. *et al.* Clinical features, identification, antimicrobial resistance patterns of Nocardia species in China: 2009–2017. *Diagn. Microbiol. Infect. Dis.* **94**, 165–172 (2019).
18. Zhao, P. *et al.* Susceptibility profiles of Nocardia spp. to antimicrobial and antituberculotic agents detected by a microplate Alamar Blue assay. *Sci. Rep.* **7**, 1–8 (2017).
19. Wei, M. *et al.* Identification and antimicrobial susceptibility of clinical Nocardia species in a tertiary hospital in China. *J. Glob. Antimicrob. Resist.* **11**, 183–187 (2017).
20. Lai, C. C. *et al.* Multicenter study in Taiwan of the in Vitro activities of nemonoxacin, tigecycline, doripenem, and other antimicrobial agents against clinical isolates of various Nocardia species. *Antimicrob. Agents Chemother.* **55**, 2084–2091 (2011).
21. Tremblay, J., Thibert, L., Alarie, I., Valiquette, L. & Pépin, J. Nocardiosis in Quebec, Canada, 1988- 2008. *Clin. Microbiol. Infect.* **17**, 690–696 (2011).
22. YE, T., SC, C. & CL, H. Antimicrobial susceptibility profiles and species distribution of medically relevant Nocardia species: Results from a large tertiary laboratory in Australia. *J. Glob. Antimicrob. Resist.* **20**, 110–117 (2020).
23. Yi, M. *et al.* Species distribution and antibiotic susceptibility of Nocardia isolates from Yantai, China. *Infect. Drug Resist.* **12**, 3653–3661 (2019).
24. Brown-Elliott, B. A. *et al.* Sulfonamide resistance in isolates of Nocardia spp. from a U.S. multicenter survey. *J. Clin. Microbiol.* **50**, 670–672 (2012).
25. Snydman, D. R. *et al.* Evaluation of the in vitro activity of eravacycline against a broad spectrum of recent clinical anaerobic isolates. *Antimicrob. Agents Chemother.* **62**, 1–8 (2018).
26. Michael, G. B. & Schwarz, S. Antimicrobial resistance in zoonotic nontyphoidal Salmonella: an alarming trend? *Clin. Microbiol. Infect.* **22**, 968–974 (2016).
27. Tack, B. *et al.* Non-typhoidal salmonella bloodstream infections in Kisantu, DR Congo: Emergence of O5-negative salmonella typhimurium and extensive drug resistance. *PLoS Negl. Trop. Dis.* **14**, 1–22 (2020).
28. Gunell, M. *et al.* In vitro activity of azithromycin against nontyphoidal Salmonella enterica.

*Antimicrob. Agents Chemother.* **54**, 3498–3501 (2010).

1. DRUG-RESISTANT NONTYPHOIDAL SALMONELLA.
2. Tyrrell, K. L. *et al.* In vitro activities of daptomycin, vancomycin, and penicillin against Clostridium difficile, C. perfringens, Finegoldia magna, and Propionibacterium acnes. *Antimicrob. Agents Chemother.* **50**, 2728–2731 (2006).
3. Wright, T. E., Boyle, K. K., Duquin, T. R. & Crane, J. K. Propionibacterium acnes Susceptibility and Correlation with Hemolytic Phenotype . *Infect. Dis. Res. Treat.* **9**, IDRT.S40539 (2016).
4. Crane, J. K., Hohman, D. W., Nodzo, S. R. & Duquin, T. R. Antimicrobial susceptibility of Propionibacterium acnes isolates from shoulder surgery. *Antimicrob. Agents Chemother.* **57**, 3424–3426 (2013).
5. Giannopoulos, L. *et al.* MLST typing of antimicrobial-resistant Propionibacterium acnes isolates from patients with moderate to severe acne vulgaris. *Anaerobe* **31**, 50–54 (2015).
6. Smith, M. A. *et al.* 341024.Pdf. **34**, 1024–1026 (1996).
7. Shames, R., Satti, F., Vellozzi, E. M. & Smith, M. A. Susceptibilities of Propionibacterium acnes

ophthalmic isolates to ertapenem, meropenem, cefepime. *J. Clin. Microbiol.* **44**, 4227–4228 (2006).

1. Rylander, M., Nord, C. E. & Norrby, S. R. Comparative in vitro activity of the new oral cephalosporin bay v 3522 against aerobic and anaerobic bacteria. *Eur. J. Clin. Microbiol. Infect. Dis.* **9**, 777–782 (1990).
2. Leyden, J. J. Current issues in antimicrobial therapy for the treatment of acne. *J. Eur. Acad. Dermatology Venereol.* **15**, 51–55 (2001).
3. Friling, E. & Montan, P. Bacteriology and cefuroxime resistance in endophthalmitis following cataract surgery before and after the introduction of prophylactic intracameral cefuroxime: a retrospective single-centre study. *J. Hosp. Infect.* **101**, 88–92 (2019).
4. Zhang, N., Yuan, R., Xin, K. Z., Lu, Z. & Ma, Y. Antimicrobial Susceptibility, Biotypes and Phylotypes of Clinical Cutibacterium (Formerly Propionibacterium) acnes Strains Isolated from Acne Patients: An Observational Study. *Dermatol. Ther. (Heidelb).* **9**, 735–746 (2019).
5. Dessinioti, C. & Katsambas, A. Propionibacterium acnes and antimicrobial resistance in acne. *Clin. Dermatol.* **35**, 163–167 (2017).
6. Walsh, T. R., Efthimiou, J. & Dréno, B. Systematic review of antibiotic resistance in acne: An increasing topical and oral threat. *Lancet Infect. Dis.* **16**, e23–e33 (2016).
7. Khassebaf, J. *et al.* Antibiotic susceptibility of Propionibacterium acnes isolated from orthopaedic implant-associated infections. *Anaerobe* **32**, 57–62 (2015).
8. Yang, S. S. *et al.* A profile of Propionibacterium acnes resistance and sensitivity at a tertiary dermatological centre in Singapore. *Br. J. Dermatol.* **179**, 200–201 (2018).
9. R, G. *et al.* In vitro antimicrobial susceptibility of Propionibacterium acnes isolated from acne patients in northern Mexico. *Int. J. Dermatol.* **49**, 1003–1007 (2010).
10. Mercieca, L. *et al.* The Antibiotic Susceptibility Profile of Cutibacterium Acnes in Maltese Patients with Acne. *J. Clin. Aesthet. Dermatol.* **13**, 11–16 (2020).
11. Schafer, F. *et al.* Antimicrobial susceptibility and genetic characteristics of Propionibacterium acnes isolated from patients with acne. *Int. J. Dermatol.* **52**, 418–425 (2013).
12. Yang, W. & Ji, X. Analysis of the microbial species, antimicrobial sensitivity and drug resistance in 2652 patients of nursing hospital. *Heliyon* **6**, (2020).
13. Lin, M. F. *et al.* Antimicrobial Susceptibility and Molecular Epidemiology of Proteus mirabilis Isolates from Three Hospitals in Northern Taiwan. *Microb. Drug Resist.* **25**, 1338–1346 (2019).
14. Adamus-Bialek, W., Zajac, E., Parniewski, P. & Kaca, W. Comparison of antibiotic resistance patterns in collections of Escherichia coli and Proteus mirabilis uropathogenic strains. *Mol. Biol. Rep.* **40**, 3429–3435 (2013).
15. Boudjemaa, H. *et al.* Molecular drivers of emerging multidrug resistance in Proteus mirabilis clinical isolates from Algeria. *J. Glob. Antimicrob. Resist.* **18**, 249–256 (2019).
16. Chen, C. Y. *et al.* Proteus mirabilis urinary tract infection and bacteremia: Risk factors, clinical presentation, and outcomes. *J. Microbiol. Immunol. Infect.* **45**, 228–236 (2012).
17. Yayan, J., Ghebremedhin, B. & Rasche, K. Cefepime shows good efficacy and no antibiotic resistance in pneumonia caused by Serratia marcescens and Proteus mirabilis - an observational study. *BMC Pharmacol. Toxicol.* **17**, 1–9 (2016).
18. Girlich, D., Bonnin, R. A., Dortet, L. & Naas, T. Genetics of Acquired Antibiotic Resistance Genes in Proteus spp. *Front. Microbiol.* **11**, 1–21 (2020).
19. De Lorenzis, E. *et al.* Bacterial spectrum and antibiotic resistance of urinary tract infections in patients treated for upper urinary tract calculi: a multicenter analysis. *Eur. J. Clin. Microbiol. Infect. Dis.* **39**, 1971–1981 (2020).
20. Fazeli, H., Moghim, S. & Zare, D. Antimicrobial Resistance Pattern and Spectrum of Multiple-drug- resistant Enterobacteriaceae in Iranian Hospitalized Patients with Cancer. *Adv. Biomed. Res.* **7**, 69 (2018).
21. Hrbacek, J., Cermak, P. & Zachoval, R. Current antibiotic resistance trends of uropathogens in central europe: Survey from a tertiary hospital urology department 2011–2019. *Antibiotics* **9**, 1– 11 (2020).
22. Stock, I. & Wiedemann, B. Natural antibiotic susceptibility of Providencia stuartii, P. rettgeri, P. alcalifaciens and P. rustigianii strains. *J. Med. Microbiol.* **47**, 629–642 (1998).
23. Luzzaro, F. *et al.* Trends in production of extended-spectrum β-lactamases among enterobacteria of medical interest: Report of the second Italian nationwide survey. *J. Clin. Microbiol.* **44**, 1659– 1664 (2006).
24. González-Rivera, E. M. *et al.* Antibiotic resistance, virulence factors and genotyping of pseudomonas aeruginosa in public hospitals of northeastern mexico. *J. Infect. Dev. Ctries.* **13**, 374–383 (2019).
25. gholami, S., Tabatabaei, M. & Sohrabi, N. Comparison of biofilm formation and antibiotic resistance pattern of Pseudomonas aeruginosa in human and environmental isolates. *Microb. Pathog.* **109**, 94–98 (2017).
26. Ahmed, N. *et al.* Evaluation of antibiotic resistance and virulence genes among clinical isolates of Pseudomonas aeruginosa from cancer patients. *Asian Pacific J. Cancer Prev.* **21**, 1333–1338 (2020).
27. Pang, Z., Raudonis, R., Glick, B. R., Lin, T. J. & Cheng, Z. Antibiotic resistance in Pseudomonas aeruginosa: mechanisms and alternative therapeutic strategies. *Biotechnol. Adv.* **37**, 177–192 (2019).
28. Dou, Y., Huan, J., Guo, F., Zhou, Z. & Shi, Y. Pseudomonas aeruginosa prevalence, antibiotic resistance and antimicrobial use in Chinese burn wards from 2007 to 2014. *J. Int. Med. Res.* **45**, 1124–1137 (2017).
29. Sambrano, H. *et al.* Prevalence of antibiotic resistance and virulent factors in nosocomial clinical isolates of Pseudomonas aeruginosa from Panamá. *Brazilian J. Infect. Dis.* **25**, 1–8 (2021).
30. Government of Canada. Pathogen Safety Data Sheet: Rickettsia rickettsii.
31. Holman, R. C. *et al.* Analysis of risk factors for fatal Rocky Mountain spotted fever: Evidence for superiority of tetracyclines for therapy. *J. Infect. Dis.* **184**, 1437–1444 (2001).
32. Ali Shah, S. A. *et al.* Antimicrobial Sensitivity Pattern of Salmonella Typhi: Emergence of Resistant Strains. *Cureus* **12**, 10–14 (2020).
33. Marchello, C. S., Carr, S. D. & Crump, J. A. A systematic review on antimicrobial resistance among salmonella typhi worldwide. *Am. J. Trop. Med. Hyg.* **103**, 2518–2527 (2020).
34. Karkey, A., Thwaites, G. E. & Baker, S. The evolution of antimicrobial resistance in Salmonella Typhi. *Curr. Opin. Gastroenterol.* **34**, 25–30 (2018).
35. Dyson, Z. A., Klemm, E. J., Palmer, S. & Dougan, G. Antibiotic resistance and typhoid. *Clin. Infect. Dis.* **68**, S165–S170 (2019).
36. Das, S., Samajpati, S., Ray, U., Roy, I. & Dutta, S. Antimicrobial resistance and molecular subtypes of Salmonella enterica serovar Typhi isolates from Kolkata, India over a 15 years period 1998– 2012. *Int. J. Med. Microbiol.* **307**, 28–36 (2017).
37. Firmo, E. F. *et al.* Association of blaNDM-1 with blaKPC-2 and aminoglycoside-modifying enzyme genes among Klebsiella pneumoniae, Proteus mirabilis and Serratia marcescens clinical isolates in Brazil. *J. Glob. Antimicrob. Resist.* **21**, 255–261 (2020).
38. Xu, J., Du, Q., Shu, Y., Ji, J. & Dai, C. Bacteriological Profile of Chronic Suppurative Otitis Media and Antibiotic Susceptibility in a Tertiary Care Hospital in Shanghai, China. *Ear, Nose Throat J.* 0–5 (2020) doi:10.1177/0145561320923823.
39. Karlowsky, J. A. *et al.* In-vitro activity of imipenem/relebactam and key β-lactam agents against Gram-negative bacilli isolated from lower respiratory tract infection samples of intensive care unit patients – SMART Surveillance United States 2015–2017. *Int. J. Antimicrob. Agents* **55**, 105841 (2020).
40. Ballot, D. E. *et al.* A review of -multidrug-resistant Enterobacteriaceae in a neonatal unit in Johannesburg, South Africa. *BMC Pediatr.* **19**, 1–9 (2019).
41. Seifert, H., Blondeau, J. & Dowzicky, M. J. In vitro activity of tigecycline and comparators (2014– 2016) among key WHO ‘priority pathogens’ and longitudinal assessment (2004–2016) of antimicrobial resistance: a report from the T.E.S.T. study. *Int. J. Antimicrob. Agents* **52**, 474–484 (2018).
42. Zachariah, O. H., Lizzy, M. A., Rose, K. & Angela, M. M. Multiple drug resistance of Campylobacter jejuni and Shigella isolated from diarrhoeic children at Kapsabet County referral hospital, Kenya. *BMC Infect. Dis.* **21**, 4–11 (2021).
43. Hussen, S., Mulatu, G. & Yohannes Kassa, Z. Prevalence of Shigella species and its drug resistance pattern in Ethiopia: A systematic review and meta-analysis. *Ann. Clin. Microbiol. Antimicrob.* **18**, 1–11 (2019).
44. Breurec, S. *et al.* Serotype distribution and antimicrobial resistance of shigella species in Bangui, Central African Republic, from 2002 to 2013. *Am. J. Trop. Med. Hyg.* **99**, 283–286 (2018).
45. Puzari, M., Sharma, M. & Chetia, P. Emergence of antibiotic resistant Shigella species: A matter of concern. *J. Infect. Public Health* **11**, 451–454 (2018).
46. IJ, A. *et al.* Fluoroquinolone resistance mechanisms of Shigella flexneri isolated in Bangladesh.

*PLoS One* **9**, (2014).

1. HC, T., DP, T., KE, H., NR, T. & S, B. The genomic signatures of Shigella evolution, adaptation and geographical spread. *Nat. Rev. Microbiol.* **14**, 235–250 (2016).
2. Bhattacharya, D. *et al.* Changing patterns and widening of antibiotic resistance in Shigella spp. over a decade (2000-2011), Andaman Islands, India. *Epidemiol. Infect.* **143**, 470–477 (2015).
3. Rothe, K. *et al.* Antimicrobial resistance of bacteraemia in the emergency department of a German university hospital (2013-2018): Potential carbapenem-sparing empiric treatment options in light of the new EUCAST recommendations. *BMC Infect. Dis.* **19**, 1–10 (2019).
4. Nathwani, D., Davey, P. G. arne. & Marwick, C. A. n. MRSA: treating people with infection. *BMJ Clin. Evid.* **2010**, 1–18 (2010).
5. Zhanel, G. G., Wiebe, R., Dilay, L., Thomson, K. & Rubinstein, E. Comparative review of the carbapenems - Focus on doripenem. *Chemother. J.* **19**, 131–149 (2010).
6. Farrell, D. J., Castanheira, M., Mendes, R. E., Sader, H. S. & Jones, R. N. In vitro activity of ceftaroline against multidrug-resistant staphylococcus aureus and streptococcus pneumoniae: A review of published studies and the AWARE surveillance program (2008-2010). *Clin. Infect. Dis.* **55**, 206–214 (2012).
7. Oladipo, A. O., Oladipo, O. G. & Bezuidenhout, C. C. Multi-drug resistance traits of methicillin- resistant Staphylococcus aureus and other Staphylococcal species from clinical and environmental sources. *J. Water Health* **17**, 930–943 (2019).
8. RA, A. *et al.* Analysis of Staphylococcus aureus clinical isolates with reduced susceptibility to ceftaroline: an epidemiological and structural perspective. *J. Antimicrob. Chemother.* **69**, 2065– 2075 (2014).
9. SW, L. *et al.* PBP2a mutations causing high-level Ceftaroline resistance in clinical methicillin- resistant Staphylococcus aureus isolates. *Antimicrob. Agents Chemother.* **58**, 6668–6674 (2014).
10. H, L. *et al.* Antimicrobial resistance of major clinical pathogens in South Korea, May 2016 to April 2017: first one-year report from Kor-GLASS. *Euro Surveill.* **23**, (2018).
11. Biedenbach, D. J. *et al.* In Vitro Activity of Oral Antimicrobial Agents against Pathogens Associated with Community-Acquired Upper Respiratory Tract and Urinary Tract Infections: A Five Country Surveillance Study. *Infect. Dis. Ther.* **5**, 139–153 (2016).
12. HS, S., DJ, F., RK, F. & RN, J. Activity of ceftaroline and comparator agents tested against Staphylococcus aureus from patients with bloodstream infections in US medical centres (2009- 13). *J. Antimicrob. Chemother.* **70**, 2053–2056 (2015).
13. A, F. *et al.* Staphylococcal resistance against five groups of life saving antibiotics in the year 2003- 2005. *Pak. J. Pharm. Sci.* **26**, 1137–1140 (2013).
14. Boada, A. *et al.* Previous antibiotic exposure and antibiotic resistance of commensal Staphylococcus aureus in Spanish primary care. *Eur. J. Gen. Pract.* **24**, 125–130 (2018).
15. Laub, K., Tóthpál, A., Kardos, S. & Dobay, O. Epidemiology and antibiotic sensitivity of staphylococcus aureus nasal carriage in children in Hungary. *Acta Microbiol. Immunol. Hung.* **64**, 51–62 (2017).
16. Kim, D. *et al.* Increasing resistance to extended-spectrum cephalosporins, fluoroquinolone, and

carbapenem in gram-negative bacilli and the emergence of carbapenem non-susceptibility in klebsiella pneumoniae: Analysis of Korean Antimicrobial Resistance Monitoring System . *Ann. Lab. Med.* **37**, 231–239 (2017).

1. Matsumoto, T. & Muratani, T. Newer carbapenems for urinary tract infections. *Int. J. Antimicrob. Agents* **24**, 35–38 (2004).
2. Ullah, O. *et al.* Antibiotic sensitivity pattern of bacterial isolates of neonatal septicemia in Peshawar, Pakistan. *Arch. Iran. Med.* **19**, 866–869 (2016).
3. Pfaller, M. A. & Jones, R. N. A review of the in vitro activity of meropenem and comparative antimicrobial agents tested against 30,254 aerobic and anaerobic pathogens isolated world wide. *Diagn. Microbiol. Infect. Dis.* **28**, 157–163 (1997).
4. Paradisi, F., Corti, G. & Messeri, D. Antistaphylococcal (mssa, mrsa, msse , mrse) antibiotics. **85**, 1–17 (2001).
5. Sendi, P. *et al.* Gentamicin Resistance in Streptococcus agalactiae. *Antimicrob. Agents Chemother.* **60**, 1702–1707 (2016).
6. Bolukaoto, J. Y. *et al.* Antibiotic resistance of Streptococcus agalactiae isolated from pregnant women in Garankuwa, South Africa. *BMC Res. Notes* **8**, 6–12 (2015).
7. Doumith, M. *et al.* Genomic sequences of Streptococcus agalactiae with high-level gentamicin resistance, collected in the BSAC bacteraemia surveillance. *J. Antimicrob. Chemother.* **72**, 2704– 2707 (2017).
8. Yanik, K. *et al.* Ceftaroline activity on certain respiratory tract and wound infection agents at the minimum inhibitory concentration level. *J. Infect. Dev. Ctries.* **9**, 1086–1090 (2015).
9. Karlowsky, J. A. *et al.* In Vitro activity of ceftaroline-avibactam against gram-negative and gram- positive pathogens isolated from patients in canadian hospitals from 2010 to 2012: Results from the CANWARD surveillance study. *Antimicrob. Agents Chemother.* **57**, 5600–5611 (2013).
10. Toda, H. *et al.* Laboratory surveillance of antimicrobial resistance and multidrug resistance among Streptococcus pneumoniae isolated in the Kinki region of Japan, 2001–2015. *J. Infect. Chemother.* **24**, 171–176 (2018).
11. Tsuzuki, S. *et al.* Improved penicillin susceptibility of Streptococcus pneumoniae and increased penicillin consumption in Japan, 2013-18. *PLoS One* **15**, 1–16 (2020).
12. Tadesse, B. T. *et al.* Antimicrobial resistance in Africa: A systematic review. *BMC Infect. Dis.* **17**, 1– 17 (2017).
13. Micek, S. T., Simmons, J., Hampton, N. & Kollef, M. H. Characteristics and outcomes among a hospitalized patient cohort with Streptococcus pneumoniae infection. *Medicine (Baltimore).* **99**, e20145 (2020).
14. Qiu, Y. *et al.* Microbiological profiles and antimicrobial resistance patterns of pediatric bloodstream pathogens in China, 2016–2018. *Eur. J. Clin. Microbiol. Infect. Dis.* **40**, 739–749 (2021).
15. Zafar, A. *et al.* Antibiotic susceptibility in Streptococcus pneumoniae, Haemophilus influenzae and Streptococcus pyogenes in Pakistan: A review of results from the survey of antibiotic

resistance (SOAR) 2002-15. *J. Antimicrob. Chemother.* **71**, i103–i109 (2016).

1. Poulakou, G. *et al.* Nationwide surveillance of Streptococcus pneumoniae in Greece: patterns of resistance and serotype epidemiology. *Int. J. Antimicrob. Agents* **30**, 87–92 (2007).
2. Ricciardi, W., Giubbini, G. & Laurenti, P. Surveillance and control of antibiotic resistance in the mediterranean region. *Mediterr. J. Hematol. Infect. Dis.* **8**, 1–11 (2016).
3. Pottumarthy, S., Sader, H. S. & Jones, R. N. Bactericidal activity of cefepime and ceftriaxone tested against Streptococcus pneumoniae. *Diagn. Microbiol. Infect. Dis.* **57**, 345–349 (2007).
4. Jean, S. S. *et al.* Nationwide surveillance of antimicrobial resistance among haemophilus influenzae and streptococcus pneumoniae in intensive care units in Taiwan. *Eur. J. Clin. Microbiol. Infect. Dis.* **28**, 1013–1017 (2009).
5. Low, D. E. *et al.* In vitro activity of cefepime against multidrug-resistant Gram-negative bacilli, viridans group Streptococci and Streptococcus pneumoniae from a cross-Canada surveillance study. *Can. J. Infect. Dis.* **10**, 122–127 (1999).
6. Schroeder, M. R. & Stephens, D. S. Macrolide resistance in Streptococcus pneumoniae. *Front. Cell. Infect. Microbiol.* **6**, 1–9 (2016).
7. Stacevičiene, I. *et al.* Antibiotic resistance of Streptococcus pneumoniae, isolated from nasopharynx of preschool children with acute respiratory tract infection in Lithuania. *BMC Infect. Dis.* **16**, 1–8 (2016).
8. SH, K. *et al.* Changes in serotype distribution and antimicrobial resistance of Streptococcus pneumoniae isolates from adult patients in Asia: Emergence of drug-resistant non-vaccine serotypes. *Vaccine* **38**, 6065–6073 (2020).
9. S, E. A. *et al.* Molecular detection of genes responsible for macrolide resistance among Streptococcus pneumoniae isolated in North Lebanon. *J. Infect. Public Health* **10**, 745–748 (2017).
10. Shokouhi, S., Darazam, I. A. & Yazdanpanah, A. Resistance of Streptococcus Pneumoniae to Macrolides in Iran. *Tanaffos* **18**, 104 (2019).
11. Ray, D., Saha, S., Sinha, S., Pal, N. K. & Bhattacharya, B. Molecular characterization and evaluation of the emerging antibiotic-resistant Streptococcus pyogenes from eastern India. *BMC Infect. Dis.* **16**, 1–11 (2016).
12. Montagnani, F. *et al.* Erythromycin resistance in Streptococcus pyogenes and macrolide consumption in a central Italian region. *Infection* **37**, 353–357 (2009).
13. Chang, H. *et al.* Antibiotic resistance and molecular analysis of streptococcus pyogenes isolated from healthy school children in China. *Scand. J. Infect. Dis.* **42**, 84–89 (2010).
14. Simon, C., Simon, M. & Plieth, C. In vitro activity of flomoxef in comparison to other cephalosporins. *Infection* **16**, 131–134 (1988).
15. Olzowy, B., Kresken, M., Havel, M., Hafner, D. & Körber-Irrgang, B. Antimicrobial susceptibility of bacterial isolates from patients presenting with ear, nose and throat (ENT) infections in the German community healthcare setting. *Eur. J. Clin. Microbiol. Infect. Dis.* **36**, 1685–1690 (2017).
16. Soyletir, G. *et al.* Results from the Survey of Antibiotic Resistance (SOAR) 2011-13 in Turkey. *J.*

*Antimicrob. Chemother.* **71**, i71–i83 (2016).

1. Li, H. *et al.* Molecular epidemiology and antimicrobial resistance of group a streptococcus recovered from patients in Beijing, China. *BMC Infect. Dis.* **20**, 1–9 (2020).
2. Morrissey, I., Ge, Y. & Janes, R. Activity of the new cephalosporin ceftaroline against bacteraemia isolates from patients with community-acquired pneumonia. *Int. J. Antimicrob. Agents* **33**, 515– 519 (2009).
3. Hsueh, P. R. *et al.* Telithromycin and quinupristin-dalfopristin resistance in clinical isolates of Streptococcus pyogenes: SMART program 2001 data. *Antimicrob. Agents Chemother.* **47**, 2152– 2157 (2003).
4. Süzük, S., Kaşkatepe, B. & Çetin, M. Antimicrobial susceptibility against penicillin, ampicillin and vancomycin of viridans group streptococcus in oral microbiota of patients at risk of infective endocarditis. *Infez. Med.* **24**, 190–193 (2016).
5. Guerrero-Del-Cueto, F., Ibanes-Gutiérrez, C., Velázquez-Acosta, C., Cornejo-Juárez, P. & Vilar- Compte, D. Microbiology and clinical characteristics of viridans group streptococci in patients with cancer. *Brazilian J. Infect. Dis.* **22**, 323–327 (2018).
6. Radocha, J. *et al.* Viridans group streptococci bloodstream infections in neutropenic adult patients with hematologic malignancy: Single center experience. *Folia Microbiol. (Praha).* **63**, 141–146 (2018).
7. Jones, R. N. *et al.* Update of contemporary antimicrobial resistance rates across China: Reference testing results for 12 medical centers (2011). *Diagn. Microbiol. Infect. Dis.* **77**, 258–266 (2013).
8. A, S., MS, J. & H, K. Antimicrobial susceptibility of viridans group streptococcal blood isolates to eight antimicrobial agents. *Scand. J. Infect. Dis.* **36**, 259–263 (2004).
9. S, C., HJ, H. & NY, L. Species-specific difference in antimicrobial susceptibility among viridans group streptococci. *Ann. Lab. Med.* **35**, 205–211 (2015).
10. Han, S. B. *et al.* Clinical characteristics and antibiotic susceptibility of viridans streptococcal bacteremia in children with febrile neutropenia. *Infection* **41**, 917–924 (2013).
11. Fritsche, T. R., Sader, H. S., Stillwell, M. G. & Jones, R. N. Antimicrobial activity of doripenem tested against prevalent Gram-positive pathogens: results from a global surveillance study (2003- 2007). *Diagn. Microbiol. Infect. Dis.* **63**, 440–446 (2009).
12. Fritsche, T. R., Sader, H. S. & Jones, R. N. Antimicrobial activity of ceftobiprole, a novel anti- methicillin-resistant Staphylococcus aureus cephalosporin, tested against contemporary pathogens: results from the SENTRY Antimicrobial Surveillance Program (2005-2006). *Diagn. Microbiol. Infect. Dis.* **61**, 86–95 (2008).
13. Stamm, L. V. Syphilis: Antibiotic treatment and resistance. *Epidemiol. Infect.* **143**, 1567–1574 (2015).
14. Douglas, J. M. *et al.* Penicillin treatment of syphilis: Clearing away the shadow on the land. *JAMA*

*- J. Am. Med. Assoc.* **301**, 769–771 (2009).

1. Stamm, L. V. Syphilis: Re-emergence of an old foe. *Microb. Cell* **3**, 363–370 (2016).
2. Kanai, M. *et al.* Molecular Typing and Macrolide Resistance Analyses of Treponema pallidum in Heterosexuals and Men Who Have Sex with Men in Japan, 2017. *J. Clin. Microbiol.* **57**, 1–7 (2019).
3. S, N. *et al.* Epidemiology, molecular strain types, and macrolide resistance of Treponema pallidum in Japan, 2017-2018. *J. Infect. Chemother.* **26**, 1042–1047 (2020).
4. Marks, M. Advances in the treatment of yaws. *Trop. Med. Infect. Dis.* **3**, (2018).
5. Mitjà, O., Hays, R., Rinaldi, A. C., McDermot, R. & Bassat, Q. New treatment schemes for yaws: The path toward eradication. *Clin. Infect. Dis.* **55**, 406–412 (2012).
6. Zheng, W. W. *et al.* Examination of ureaplasma urealyticum and Mycoplasma hominis in 4082 Chinese patients. *Brazilian J. Med. Biol. Res.* **54**, 1–4 (2021).
7. Zeng, X. Y., Xin, N., Tong, X. N., Wang, J. Y. & Liu, Z. W. Prevalence and antibiotic susceptibility of Ureaplasma urealyticum and Mycoplasma hominis in Xi’an, China. *Eur. J. Clin. Microbiol. Infect. Dis.* **35**, 1941–1947 (2016).
8. Lee, M., Kim, M., Lee, W., Kang, S. & Jeon, Y. Prevalence and antibiotic susceptibility of Mycoplasma hominis and Ureaplasma urealyticum in pregnant women. *Yonsei Med. J.* **57**, 1271– 1275 (2016).
9. Zhang, W. *et al.* Infection Prevalence and Antibiotic Resistance Levels in Ureaplasma urealyticum and Mycoplasma hominis in Gynecological Outpatients of a Tertiary Hospital in China from 2015 to 2018. *Can. J. Infect. Dis. Med. Microbiol.* **2021**, (2021).
10. Ahmadi, M. H. Resistance to tetracyclines among clinical isolates of Mycoplasma hominis and Ureaplasma species: a systematic review and meta-analysis. *J. Antimicrob. Chemother.* **76**, 865– 875 (2021).
11. Valentine-king, M. A. & Brown, M. B. crossm. **61**, 1–11 (2017).
12. Rijal, N. *et al.* Changing epidemiology and antimicrobial resistance in Vibrio cholerae: AMR surveillance findings (2006-2016) from Nepal. *BMC Infect. Dis.* **19**, 1–8 (2019).
13. Yousefi, A., Vaez, H., Sahebkar, A. & Khademi, F. A systematic review and meta-analysis on the epidemiology of antibiotic resistance of Vibrio cholerae in Iran. *Ann. di Ig. Med. Prev. e di Comunita* **31**, 279–290 (2019).
14. Fang, L., Ginn, A. M., Harper, J., Kane, A. S. & Wright, A. C. Survey and genetic characterization of Vibrio cholerae in Apalachicola Bay, Florida (2012–2014). *J. Appl. Microbiol.* **126**, 1265–1277 (2019).
15. Urich, S. K., Chalcraft, L., Schriefer, M. E., Yockey, B. M. & Petersen, J. M. Lack of antimicrobial resistance in Yersinia pestis isolates from 17 countries in the Americas, Africa, and Asia. *Antimicrob. Agents Chemother.* **56**, 555–558 (2012).
16. Galimand, M., Carniel, E. & Courvalin, P. Resistance of Yersinia pestis to antimicrobial agents.

*Antimicrob. Agents Chemother.* **50**, 3233–3236 (2006).

## Supplemental Table S4: Algorithmic review method raw data and citations

| **Pathogen Name** | **Drug Class** | **Antibiotic** | **Citation** |
| --- | --- | --- | --- |
| Acinetobacterspp | Carbapenems | Doripenem | 1 |
| Acinetobacterspp | Carbapenems | Imipenem | 2 |
| Acinetobacterspp | Carbapenems | Imipenem | 3 |
| Acinetobacterspp | Carbapenems | Imipenem | 4 |
| Acinetobacterspp | Carbapenems | Meropenem | 5 |
| Acinetobacterspp | Carbapenems | Meropenem | 1 |
| Acinetobacterspp | Carbapenems | Meropenem | 6 |
| Actinomycesspp | Penicillins | Penicillin | 7 |
| Actinomycesspp | Penicillins | Unasyn(ampicillin/sulbactam) | 7 |
| Bacillusanthracis | Penicillins | Ampicillin | 8 |
| Bacteroidesspp | Penicillins | Penicillin | 9 |
| Bacteroidesspp | Penicillins | Penicillin | 10 |
| Bacteroidesspp | Penicillins | Penicillin | 11 |
| Bacteroidesspp | Penicillins | Amoxicillin | 12 |
| Bacteroidesspp | Penicillins | Ampicillin | 10 |
| Bacteroidesspp | Penicillins | Ampicillin | 13 |
| Bacteroidesspp | Penicillins | Ampicillin | 14 |
| Bacteroidesspp | Penicillins | Augmentin(amoxicillin/clavulanate) | 9 |
| Bacteroidesspp | Penicillins | Augmentin(amoxicillin/clavulanate) | 10 |
| Bacteroidesspp | Penicillins | Augmentin(amoxicillin/clavulanate) | 11 |
| Bacteroidesspp | Penicillins | Unasyn(ampicillin/sulbactam) | 13 |
| Bacteroidesspp | Penicillins | Unasyn(ampicillin/sulbactam) | 14 |
| Bacteroidesspp | Penicillins | Zosyn(pipercillin/tazobactam) | 9 |
| Bacteroidesspp | Penicillins | Zosyn(pipercillin/tazobactam) | 10 |
| Bacteroidesspp | Penicillins | Zosyn(pipercillin/tazobactam) | 13 |
| Bacteroidesspp | Carbapenems | Doripenem | 13 |
| Bacteroidesspp | Carbapenems | Imipenem | 9 |

| Bacteroidesspp | Carbapenems | Imipenem | 15 |
| --- | --- | --- | --- |
| Bacteroidesspp | Carbapenems | Imipenem | 10 |
| Bacteroidesspp | Carbapenems | Meropenem | 15 |
| Bacteroidesspp | Carbapenems | Meropenem | 10 |
| Bacteroidesspp | Carbapenems | Meropenem | 11 |
| Bacteroidesspp | Carbapenems | Ertapenem | 16 |
| Bacteroidesspp | Cephalosporins(2ndgen) | Cefmetazole | 13 |
| Bacteroidesspp | Cephalosporins(2ndgen) | Cefmetazole | 14 |
| Bacteroidesspp | Cephalosporins(2ndgen) | Cefoxitin | 15 |
| Bacteroidesspp | Cephalosporins(2ndgen) | Cefoxitin | 10 |
| Bacteroidesspp | Cephalosporins(3rdgen) | Ceftriaxone | 14 |
| Bacteroidesspp | Macrolides | Clindamycin | 9 |
| Bacteroidesspp | Macrolides | Clindamycin | 15 |
| Bacteroidesspp | Macrolides | Clindamycin | 10 |
| Bacteroidesspp | Nitroimidazoles | Metronidazole | 9 |
| Bacteroidesspp | Nitroimidazoles | Metronidazole | 15 |
| Bacteroidesspp | Nitroimidazoles | Metronidazole | 10 |
| Bordetellapertussis | Macrolides | Erythromycin | 17 |
| Bordetellapertussis | Macrolides | Erythromycin | 18 |
| Bordetellapertussis | Macrolides | Erythromycin | 19 |
| Bordetellapertussis | Macrolides | Clarithromycin | 18 |
| Bordetellapertussis | Macrolides | Clarithromycin | 20 |
| Bordetellapertussis | Macrolides | Azithromycin | 18 |
| Bordetellapertussis | Macrolides | Azithromycin | 19 |
| Bordetellapertussis | Macrolides | Azithromycin | 21 |
| Bordetellapertussis | Macrolides | Clindamycin | 18 |
| Bordetellapertussis | Macrolides | Clindamycin | 21 |
| Brucellaspp | Aminoglycosides | Gentamicin | 22 |

| Brucellaspp | Aminoglycosides | Gentamicin | 23 |
| --- | --- | --- | --- |
| Brucellaspp | Fluoroquinolones | Ciprofloxacin | 22 |
| Brucellaspp | Fluoroquinolones | Ciprofloxacin | 23 |
| Brucellaspp | Fluoroquinolones | Levofloxacin | 22 |
| Brucellaspp | Fluoroquinolones | Moxifloxacin | 23 |
| Brucellaspp | Rifamycins | Rifampin | 24 |
| Brucellaspp | Rifamycins | Rifampin | 22 |
| Brucellaspp | Rifamycins | Rifampin | 23 |
| Brucellaspp | Tetracyclines | Doxycyline | 22 |
| Brucellaspp | Tetracyclines | Doxycyline | 23 |
| Brucellaspp | Tetracyclines | Minocycline | 22 |
| Brucellaspp | Tetracyclines | Tetracycline | 22 |
| Brucellaspp | Trimethoprim-  sulfamethoxazole | Trimethoprim-sulfamethoxazole | 22 |
| Brucellaspp | Trimethoprim-  sulfamethoxazole | Trimethoprim-sulfamethoxazole | 23 |
| Campylobacterjejuni | Fluoroquinolones | Ciprofloxacin | 25 |
| Campylobacterjejuni | Fluoroquinolones | Ciprofloxacin | 26 |
| Campylobacterjejuni | Fluoroquinolones | Ciprofloxacin | 27 |
| Campylobacterjejuni | Fluoroquinolones | Norfloxacin | 28 |
| Campylobacterjejuni | Macrolides | Erythromycin | 26 |
| Campylobacterjejuni | Macrolides | Erythromycin | 29 |
| Campylobacterjejuni | Macrolides | Erythromycin | 30 |
| Campylobacterjejuni | Macrolides | Azithromycin | 25 |
| Campylobacterjejuni | Macrolides | Azithromycin | 31 |
| Campylobacterjejuni | Macrolides | Azithromycin | 32 |
| Chlamydiapneumoniae | Macrolides | Erythromycin | 33 |
| Chlamydiatrachomatis | Macrolides | Erythromycin | 34 |
| Chlamydiatrachomatis | Macrolides | Clarithromycin | 34 |
| Chlamydiatrachomatis | Tetracyclines | Doxycyline | 34 |

| Chlamydiatrachomatis | Tetracyclines | Minocycline | 34 |
| --- | --- | --- | --- |
| Citrobacterspp | Aminoglycosides | Gentamicin | 35 |
| Citrobacterspp | Aminoglycosides | Amikacin | 36 |
| Citrobacterspp | Aminoglycosides | Amikacin | 35 |
| Citrobacterspp | Aminoglycosides | Tobramycin | 35 |
| Citrobacterspp | Penicillins | Ampicillin | 35 |
| Citrobacterspp | Penicillins | Ampicillin | 37 |
| Citrobacterspp | Penicillins | Augmentin(amoxicillin/clavulanate) | 36 |
| Citrobacterspp | Penicillins | Unasyn(ampicillin/sulbactam) | 35 |
| Citrobacterspp | Penicillins | Unasyn(ampicillin/sulbactam) | 37 |
| Citrobacterspp | Penicillins | Zosyn(pipercillin/tazobactam) | 38 |
| Citrobacterspp | Penicillins | Zosyn(pipercillin/tazobactam) | 35 |
| Citrobacterspp | Penicillins | Zosyn(pipercillin/tazobactam) | 39 |
| Citrobacterspp | Carbapenems | Doripenem | 36 |
| Citrobacterspp | Carbapenems | Imipenem | 40 |
| Citrobacterspp | Carbapenems | Imipenem | 36 |
| Citrobacterspp | Carbapenems | Meropenem | 40 |
| Citrobacterspp | Carbapenems | Meropenem | 36 |
| Citrobacterspp | Cephalosporins(2ndgen) | Cefoxitin | 38 |
| Citrobacterspp | Cephalosporins(2ndgen) | Cefoxitin | 35 |
| Citrobacterspp | Cephalosporins(3rdgen) | Ceftazidime | 36 |
| Citrobacterspp | Cephalosporins(3rdgen) | Ceftazidime | 37 |
| Citrobacterspp | Cephalosporins(3rdgen) | Ceftazidime | 35 |
| Citrobacterspp | Cephalosporins(3rdgen) | Ceftriaxone | 37 |
| Citrobacterspp | Cephalosporins(3rdgen) | Ceftriaxone | 35 |
| Citrobacterspp | Tetracyclines | Doxycyline | 39 |
| Citrobacterspp | Tetracyclines | Minocycline | 39 |
| Citrobacterspp | Tetracyclines | Tetracycline | 39 |

| Citrobacterspp | Tetracyclines | Tetracycline | 35 |
| --- | --- | --- | --- |
| Citrobacterspp | Trimethoprim-  sulfamethoxazole | Trimethoprim-sulfamethoxazole | 35 |
| Clostridiumdifficile | Glycopeptideantibiotics | Vancomycin | 10 |
| Clostridiumdifficile | Glycopeptideantibiotics | Vancomycin | 41 |
| Clostridiumdifficile | Glycopeptideantibiotics | Vancomycin | 42 |
| Clostridiumdifficile | Glycopeptideantibiotics | Teicoplanin | 43 |
| Clostridiumperfringens | Penicillins | Penicillin | 44 |
| Clostridiumperfringens | Penicillins | Penicillin | 45 |
| Clostridiumperfringens | Penicillins | Unasyn(ampicillin/sulbactam) | 46 |
| Clostridiumperfringens | Penicillins | Zosyn(pipercillin/tazobactam) | 46 |
| Clostridiumspp | Penicillins | Penicillin | 9 |
| Clostridiumspp | Penicillins | Penicillin | 44 |
| Clostridiumspp | Penicillins | Augmentin(amoxicillin/clavulanate) | 9 |
| Clostridiumspp | Penicillins | Unasyn(ampicillin/sulbactam) | 46 |
| Clostridiumspp | Penicillins | Zosyn(pipercillin/tazobactam) | 46 |
| Clostridiumspp | Carbapenems | Imipenem | 9 |
| Clostridiumspp | Carbapenems | Imipenem | 46 |
| Clostridiumspp | Carbapenems | Meropenem | 46 |
| Corynebacteriumdiptheriae | Penicillins | Penicillin | 47 |
| Corynebacteriumdiptheriae | Penicillins | Penicillin | 48 |
| Corynebacteriumdiptheriae | Penicillins | Penicillin | 49 |
| Corynebacteriumdiptheriae | Macrolides | Erythromycin | 48 |
| Corynebacteriumdiptheriae | Macrolides | Erythromycin | 49 |
| Corynebacteriumdiptheriae | Macrolides | Erythromycin | 50 |
| Enterobacteraerogenes | Carbapenems | Doripenem | 51 |
| Enterobacteraerogenes | Carbapenems | Imipenem | 52 |
| Enterobacteraerogenes | Carbapenems | Imipenem | 53 |
| Enterobacteraerogenes | Carbapenems | Imipenem | 51 |

| Enterobacteraerogenes | Carbapenems | Meropenem | 51 |
| --- | --- | --- | --- |
| Enterobacteraerogenes | Carbapenems | Ertapenem | 51 |
| Enterobacteraerogenes | Cephalosporins(3rdgen) | Cefotaxime | 51 |
| Enterobacteraerogenes | Cephalosporins(3rdgen) | Ceftazidime | 51 |
| Enterobacteraerogenes | Cephalosporins(4thgen) | Cefepime | 54 |
| Enterobacteraerogenes | Cephalosporins(4thgen) | Cefepime | 51 |
| Enterobacteraerogenes | Fluoroquinolones | Ciprofloxacin | 51 |
| Enterobacteraerogenes | Fluoroquinolones | Levofloxacin | 51 |
| Enterococcusfaecalis | Aminoglycosides | Gentamicin | 55 |
| Enterococcusfaecalis | Aminoglycosides | Gentamicin | 56 |
| Enterococcusfaecalis | Aminoglycosides | Gentamicin | 57 |
| Enterococcusfaecalis | Aminoglycosides | Amikacin | 58 |
| Enterococcusfaecalis | Aminoglycosides | Amikacin | 59 |
| Enterococcusfaecalis | Aminoglycosides | Amikacin | 60 |
| Enterococcusfaecalis | Carbapenems | Imipenem | 61 |
| Enterococcusfaecalis | Carbapenems | Imipenem | 62 |
| Escherichiacoli *(ETEC)* | Carbapenems | Doripenem | 63 |
| Escherichiacoli *(ETEC)* | Carbapenems | Doripenem | 64 |
| Escherichiacoli *(ETEC)* | Carbapenems | Imipenem | 65 |
| Escherichiacoli *(ETEC)* | Carbapenems | Imipenem | 61 |
| Escherichiacoli *(ETEC)* | Carbapenems | Imipenem | 66 |
| Escherichiacoli *(ETEC)* | Carbapenems | Meropenem | 61 |
| Escherichiacoli *(ETEC)* | Carbapenems | Meropenem | 67 |
| Escherichiacoli *(ETEC)* | Carbapenems | Meropenem | 66 |
| Escherichiacoli *(ETEC)* | Carbapenems | Ertapenem | 61 |
| Escherichiacoli *(ETEC)* | Carbapenems | Ertapenem | 68 |
| Escherichiacoli *(ETEC)* | Carbapenems | Ertapenem | 63 |
| Escherichiacoli *(ETEC)* | Cephalosporins(1stgen) | Cefazolin | 69 |

| Escherichiacoli *(ETEC)* | Cephalosporins(1stgen) | Cefazolin | 63 |
| --- | --- | --- | --- |
| Escherichiacoli *(ETEC)* | Cephalosporins(1stgen) | Cefazolin | 70 |
| Escherichiacoli *(ETEC)* | Cephalosporins(1stgen) | Cephalothin | 71 |
| Escherichiacoli *(ETEC)* | Cephalosporins(1stgen) | Cephalothin | 72 |
| Escherichiacoli *(ETEC)* | Cephalosporins(1stgen) | Cephalothin | 73 |
| Escherichiacoli *(ETEC)* | Cephalosporins(1stgen) | Cephradine | 74 |
| Escherichiacoli *(ETEC)* | Cephalosporins(1stgen) | Cephalexin | 75 |
| Escherichiacoli *(ETEC)* | Cephalosporins(1stgen) | Cephalexin | 76 |
| Escherichiacoli *(ETEC)* | Cephalosporins(1stgen) | Cephalexin | 77 |
| Escherichiacoli *(ETEC)* | Cephalosporins(2ndgen) | Cefmetazole | 78 |
| Escherichiacoli *(ETEC)* | Cephalosporins(2ndgen) | Cefmetazole | 79 |
| Escherichiacoli *(ETEC)* | Cephalosporins(2ndgen) | Cefoxitin | 68 |
| Escherichiacoli *(ETEC)* | Cephalosporins(2ndgen) | Cefoxitin | 80 |
| Escherichiacoli *(ETEC)* | Cephalosporins(2ndgen) | Cefoxitin | 81 |
| Escherichiacoli *(ETEC)* | Cephalosporins(2ndgen) | Cefuroxime | 82 |
| Escherichiacoli *(ETEC)* | Cephalosporins(2ndgen) | Cefuroxime | 83 |
| Escherichiacoli *(ETEC)* | Cephalosporins(2ndgen) | Cefuroxime | 84 |
| Escherichiacoli *(ETEC)* | Cephalosporins(2ndgen) | Cefaclor | 75 |
| Escherichiacoli *(ETEC)* | Cephalosporins(2ndgen) | Cefaclor | 85 |
| Escherichiacoli *(ETEC)* | Cephalosporins(2ndgen) | Cefaclor | 86 |
| Escherichiacoli *(ETEC)* | Cephalosporins(3rdgen) | Cefotaxime | 87 |
| Escherichiacoli *(ETEC)* | Cephalosporins(3rdgen) | Cefotaxime | 68 |
| Escherichiacoli *(ETEC)* | Cephalosporins(3rdgen) | Cefotaxime | 88 |
| Escherichiacoli *(ETEC)* | Cephalosporins(3rdgen) | Ceftazidime | 68 |
| Escherichiacoli *(ETEC)* | Cephalosporins(3rdgen) | Ceftazidime | 89 |
| Escherichiacoli *(ETEC)* | Cephalosporins(3rdgen) | Ceftazidime | 88 |
| Escherichiacoli *(ETEC)* | Cephalosporins(3rdgen) | Ceftriaxone | 68 |
| Escherichiacoli *(ETEC)* | Cephalosporins(3rdgen) | Ceftriaxone | 90 |

| Escherichiacoli *(ETEC)* | Cephalosporins(3rdgen) | Ceftriaxone | 91 |
| --- | --- | --- | --- |
| Escherichiacoli *(ETEC)* | Cephalosporins(3rdgen) | Cefpodoxime | 92 |
| Escherichiacoli *(ETEC)* | Cephalosporins(3rdgen) | Cefpodoxime | 70 |
| Escherichiacoli *(ETEC)* | Cephalosporins(3rdgen) | Cefpodoxime | 93 |
| Escherichiacoli *(ETEC)* | Cephalosporins(3rdgen) | Cefixime | 92 |
| Escherichiacoli *(ETEC)* | Cephalosporins(3rdgen) | Cefixime | 70 |
| Escherichiacoli *(ETEC)* | Cephalosporins(3rdgen) | Cefixime | 94 |
| Escherichiacoli *(ETEC)* | Cephalosporins(4thgen) | Cefepime | 95 |
| Escherichiacoli *(ETEC)* | Cephalosporins(4thgen) | Cefepime | 87 |
| Escherichiacoli *(ETEC)* | Cephalosporins(4thgen) | Cefepime | 68 |
| Escherichiacoli *(ETEC)* | Cephalosporins(5thgen) | Ceftaroline | 96 |
| Escherichiacoli *(ETEC)* | Cephalosporins(5thgen) | Ceftaroline | 97 |
| Escherichiacoli *(ETEC)* | Cephalosporins(5thgen) | Ceftaroline | 98 |
| Escherichiacoli *(ETEC)* | Fluoroquinolones | Ciprofloxacin | 67 |
| Escherichiacoli *(ETEC)* | Fluoroquinolones | Ciprofloxacin | 68 |
| Escherichiacoli *(ETEC)* | Fluoroquinolones | Ciprofloxacin | 99 |
| Escherichiacoli *(ETEC)* | Fluoroquinolones | Levofloxacin | 100 |
| Escherichiacoli *(ETEC)* | Fluoroquinolones | Levofloxacin | 68 |
| Escherichiacoli *(ETEC)* | Fluoroquinolones | Levofloxacin | 69 |
| Escherichiacoli *(ETEC)* | Fluoroquinolones | Moxifloxacin | 101 |
| Escherichiacoli *(ETEC)* | Fluoroquinolones | Norfloxacin | 102 |
| Escherichiacoli *(ETEC)* | Fluoroquinolones | Norfloxacin | 103 |
| Escherichiacoli *(ETEC)* | Fluoroquinolones | Norfloxacin | 104 |
| Escherichiacoli *(ETEC)* | Fluoroquinolones | Ofloxacin | 105 |
| Escherichiacoli *(ETEC)* | Fluoroquinolones | Ofloxacin | 106 |
| Escherichiacoli *(ETEC)* | Fluoroquinolones | Ofloxacin | 107 |
| Francisellatularensis | Aminoglycosides | Gentamicin | 108 |
| Francisellatularensis | Aminoglycosides | Gentamicin | 109 |

| Francisellatularensis | Aminoglycosides | Tobramycin | 108 |
| --- | --- | --- | --- |
| Francisellatularensis | Fluoroquinolones | Ciprofloxacin | 108 |
| Francisellatularensis | Fluoroquinolones | Ciprofloxacin | 109 |
| Francisellatularensis | Fluoroquinolones | Levofloxacin | 108 |
| Francisellatularensis | Fluoroquinolones | Moxifloxacin | 108 |
| Francisellatularensis | Fluoroquinolones | Ofloxacin | 108 |
| Francisellatularensis | Tetracyclines | Doxycyline | 108 |
| Francisellatularensis | Tetracyclines | Doxycyline | 109 |
| Fusobacteriumspp | Penicillins | Penicillin | 10 |
| Fusobacteriumspp | Penicillins | Ampicillin | 10 |
| Fusobacteriumspp | Penicillins | Augmentin(amoxicillin/clavulanate) | 10 |
| Fusobacteriumspp | Penicillins | Unasyn(ampicillin/sulbactam) | 13 |
| Fusobacteriumspp | Penicillins | Unasyn(ampicillin/sulbactam) | 14 |
| Fusobacteriumspp | Penicillins | Zosyn(pipercillin/tazobactam) | 10 |
| Fusobacteriumspp | Penicillins | Zosyn(pipercillin/tazobactam) | 13 |
| Fusobacteriumspp | Carbapenems | Doripenem | 13 |
| Fusobacteriumspp | Carbapenems | Imipenem | 10 |
| Fusobacteriumspp | Carbapenems | Imipenem | 13 |
| Fusobacteriumspp | Carbapenems | Meropenem | 10 |
| Fusobacteriumspp | Cephalosporins(2ndgen) | Cefmetazole | 13 |
| Fusobacteriumspp | Cephalosporins(2ndgen) | Cefmetazole | 14 |
| Fusobacteriumspp | Cephalosporins(2ndgen) | Cefoxitin | 10 |
| Gardnerellavaginalis | Macrolides | Clindamycin | 110 |
| Gardnerellavaginalis | Macrolides | Clindamycin | 111 |
| Gardnerellavaginalis | Macrolides | Clindamycin | 112 |
| Gardnerellavaginalis | Nitroimidazoles | Metronidazole | 110 |
| Gardnerellavaginalis | Nitroimidazoles | Metronidazole | 111 |
| Gardnerellavaginalis | Nitroimidazoles | Metronidazole | 112 |

| Gardnerellavaginalis | Nitroimidazoles | Tinidazole | 112 |
| --- | --- | --- | --- |
| GPAC | Penicillins | Penicillin | 9 |
| GPAC | Penicillins | Penicillin | 113 |
| GPAC | Penicillins | Penicillin | 114 |
| GPAC | Penicillins | Augmentin(amoxicillin/clavulanate) | 9 |
| GPAC | Penicillins | Augmentin(amoxicillin/clavulanate) | 115 |
| GPAC | Penicillins | Unasyn(ampicillin/sulbactam) | 9 |
| GPAC | Penicillins | Unasyn(ampicillin/sulbactam) | 11 |
| GPAC | Penicillins | Unasyn(ampicillin/sulbactam) | 115 |
| GPAC | Penicillins | Zosyn(pipercillin/tazobactam) | 116 |
| GPAC | Penicillins | Zosyn(pipercillin/tazobactam) | 11 |
| GPAC | Penicillins | Zosyn(pipercillin/tazobactam) | 11 |
| GPAC | Carbapenems | Imipenem | 9 |
| GPAC | Carbapenems | Imipenem | 11 |
| GPAC | Carbapenems | Imipenem | 113 |
| GPAC | Cephalosporins(2ndgen) | Cefotetan | 113 |
| GPAC | Cephalosporins(2ndgen) | Cefoxitin | 113 |
| GPAC | Cephalosporins(2ndgen) | Cefoxitin | 115 |
| GPAC | Macrolides | Clindamycin | 9 |
| GPAC | Macrolides | Clindamycin | 11 |
| GPAC | Macrolides | Clindamycin | 113 |
| GPAC | Nitroimidazoles | Metronidazole | 9 |
| GPAC | Nitroimidazoles | Metronidazole | 11 |
| GPAC | Nitroimidazoles | Metronidazole | 113 |
| GPAC | Tetracyclines | Tetracycline | 113 |
| Haemophilusinfluenzae | Penicillins | Penicillin | 117 |
| Haemophilusinfluenzae | Penicillins | Amoxicillin | 118 |
| Haemophilusinfluenzae | Penicillins | Amoxicillin | 119 |

| Haemophilusinfluenzae | Penicillins | Amoxicillin | 120 |
| --- | --- | --- | --- |
| Haemophilusinfluenzae | Penicillins | Ampicillin | 118 |
| Haemophilusinfluenzae | Penicillins | Ampicillin | 119 |
| Haemophilusinfluenzae | Penicillins | Ampicillin | 120 |
| Haemophilusinfluenzae | Penicillins | Augmentin(amoxicillin/clavulanate) | 118 |
| Haemophilusinfluenzae | Penicillins | Augmentin(amoxicillin/clavulanate) | 119 |
| Haemophilusinfluenzae | Penicillins | Augmentin(amoxicillin/clavulanate) | 120 |
| Haemophilusinfluenzae | Penicillins | Unasyn(ampicillin/sulbactam) | 121 |
| Haemophilusinfluenzae | Penicillins | Unasyn(ampicillin/sulbactam) | 122 |
| Haemophilusinfluenzae | Penicillins | Unasyn(ampicillin/sulbactam) | 123 |
| Haemophilusinfluenzae | Penicillins | Zosyn(pipercillin/tazobactam) | 123 |
| Haemophilusinfluenzae | Carbapenems | Imipenem | 124 |
| Haemophilusinfluenzae | Carbapenems | Imipenem | 125 |
| Haemophilusinfluenzae | Carbapenems | Meropenem | 122 |
| Haemophilusinfluenzae | Carbapenems | Meropenem | 126 |
| Haemophilusinfluenzae | Cephalosporins(2ndgen) | Cefuroxime | 118 |
| Haemophilusinfluenzae | Cephalosporins(2ndgen) | Cefuroxime | 119 |
| Haemophilusinfluenzae | Cephalosporins(2ndgen) | Cefuroxime | 120 |
| Haemophilusinfluenzae | Cephalosporins(2ndgen) | Cefaclor | 118 |
| Haemophilusinfluenzae | Cephalosporins(2ndgen) | Cefaclor | 119 |
| Haemophilusinfluenzae | Cephalosporins(2ndgen) | Cefaclor | 120 |
| Haemophilusinfluenzae | Cephalosporins(3rdgen) | Cefotaxime | 122 |
| Haemophilusinfluenzae | Cephalosporins(3rdgen) | Cefotaxime | 127 |
| Haemophilusinfluenzae | Cephalosporins(3rdgen) | Cefotaxime | 128 |
| Haemophilusinfluenzae | Cephalosporins(3rdgen) | Cefdinir | 129 |
| Haemophilusinfluenzae | Cephalosporins(3rdgen) | Cefdinir | 120 |
| Haemophilusinfluenzae | Cephalosporins(3rdgen) | Ceftriaxone | 118 |
| Haemophilusinfluenzae | Cephalosporins(3rdgen) | Ceftriaxone | 119 |

| Haemophilusinfluenzae | Cephalosporins(3rdgen) | Ceftriaxone | 120 |
| --- | --- | --- | --- |
| Haemophilusinfluenzae | Cephalosporins(3rdgen) | Cefpodoxime | 129 |
| Haemophilusinfluenzae | Cephalosporins(3rdgen) | Cefpodoxime | 130 |
| Haemophilusinfluenzae | Cephalosporins(3rdgen) | Cefpodoxime | 131 |
| Haemophilusinfluenzae | Cephalosporins(3rdgen) | Cefixime | 119 |
| Haemophilusinfluenzae | Cephalosporins(3rdgen) | Cefixime | 130 |
| Haemophilusinfluenzae | Cephalosporins(3rdgen) | Cefixime | 132 |
| Haemophilusinfluenzae | Cephalosporins(5thgen) | Ceftaroline | 126 |
| Haemophilusinfluenzae | Cephalosporins(5thgen) | Ceftaroline | 133 |
| Haemophilusinfluenzae | Cephalosporins(5thgen) | Ceftaroline | 97 |
| Haemophilusinfluenzae | Fluoroquinolones | Ciprofloxacin | 134 |
| Haemophilusinfluenzae | Fluoroquinolones | Ciprofloxacin | 124 |
| Haemophilusinfluenzae | Fluoroquinolones | Ciprofloxacin | 135 |
| Haemophilusinfluenzae | Fluoroquinolones | Levofloxacin | 118 |
| Haemophilusinfluenzae | Fluoroquinolones | Levofloxacin | 119 |
| Haemophilusinfluenzae | Fluoroquinolones | Levofloxacin | 120 |
| Haemophilusinfluenzae | Fluoroquinolones | Moxifloxacin | 136 |
| Haemophilusinfluenzae | Fluoroquinolones | Moxifloxacin | 137 |
| Haemophilusinfluenzae | Fluoroquinolones | Moxifloxacin | 134 |
| Haemophilusinfluenzae | Fluoroquinolones | Ofloxacin | 134 |
| Haemophilusinfluenzae | Macrolides | Erythromycin | 138 |
| Haemophilusinfluenzae | Macrolides | Clarithromycin | 118 |
| Haemophilusinfluenzae | Macrolides | Clarithromycin | 119 |
| Haemophilusinfluenzae | Macrolides | Clarithromycin | 120 |
| Haemophilusinfluenzae | Macrolides | Azithromycin | 134 |
| Haemophilusinfluenzae | Macrolides | Azithromycin | 122 |
| Haemophilusinfluenzae | Macrolides | Azithromycin | 135 |
| Haemophilusinfluenzae | Trimethoprim-  sulfamethoxazole | Trimethoprim-sulfamethoxazole | 118 |

| Haemophilusinfluenzae | Trimethoprim-  sulfamethoxazole | Trimethoprim-sulfamethoxazole | 119 |
| --- | --- | --- | --- |
| Haemophilusinfluenzae | Trimethoprim-  sulfamethoxazole | Trimethoprim-sulfamethoxazole | 120 |
| Klebsiellaoxytoca | Cephalosporins(5thgen) | Ceftaroline | 98 |
| Klebsiellaoxytoca | Cephalosporins(5thgen) | Ceftaroline | 139 |
| Klebsiellapneumoniae | Carbapenems | Imipenem | 140 |
| Klebsiellapneumoniae | Carbapenems | Imipenem | 141 |
| Klebsiellapneumoniae | Carbapenems | Imipenem | 142 |
| Klebsiellapneumoniae | Carbapenems | Meropenem | 140 |
| Klebsiellapneumoniae | Carbapenems | Meropenem | 141 |
| Klebsiellapneumoniae | Carbapenems | Meropenem | 143 |
| Klebsiellapneumoniae | Carbapenems | Ertapenem | 68 |
| Klebsiellapneumoniae | Carbapenems | Ertapenem | 144 |
| Klebsiellapneumoniae | Carbapenems | Ertapenem | 145 |
| Klebsiellapneumoniae | Carbapenems | Imipenem/Cilastatin | 146 |
| Klebsiellapneumoniae | Cephalosporins(1stgen) | Cefazolin | 141 |
| Klebsiellapneumoniae | Cephalosporins(1stgen) | Cefazolin | 147 |
| Klebsiellapneumoniae | Cephalosporins(1stgen) | Cefazolin | 148 |
| Klebsiellapneumoniae | Cephalosporins(1stgen) | Cephalothin | 149 |
| Klebsiellapneumoniae | Cephalosporins(1stgen) | Cephalothin | 150 |
| Klebsiellapneumoniae | Cephalosporins(1stgen) | Cephalothin | 151 |
| Klebsiellapneumoniae | Cephalosporins(1stgen) | Cephalothin | 152 |
| Klebsiellapneumoniae | Cephalosporins(1stgen) | Cephalexin | 153 |
| Klebsiellapneumoniae | Cephalosporins(2ndgen) | Cefotetan | 154 |
| Klebsiellapneumoniae | Cephalosporins(2ndgen) | Cefotetan | 70 |
| Klebsiellapneumoniae | Cephalosporins(2ndgen) | Cefoxitin | 141 |
| Klebsiellapneumoniae | Cephalosporins(2ndgen) | Cefoxitin | 68 |
| Klebsiellapneumoniae | Cephalosporins(2ndgen) | Cefoxitin | 142 |
| Klebsiellapneumoniae | Cephalosporins(2ndgen) | Cefuroxime | 141 |

| Klebsiellapneumoniae | Cephalosporins(2ndgen) | Cefuroxime | 149 |
| --- | --- | --- | --- |
| Klebsiellapneumoniae | Cephalosporins(2ndgen) | Cefuroxime | 147 |
| Klebsiellapneumoniae | Cephalosporins(2ndgen) | Cefaclor | 70 |
| Klebsiellapneumoniae | Cephalosporins(3rdgen) | Cefotaxime | 68 |
| Klebsiellapneumoniae | Cephalosporins(3rdgen) | Cefotaxime | 88 |
| Klebsiellapneumoniae | Cephalosporins(3rdgen) | Cefotaxime | 37 |
| Klebsiellapneumoniae | Cephalosporins(3rdgen) | Ceftazidime | 155 |
| Klebsiellapneumoniae | Cephalosporins(3rdgen) | Ceftazidime | 141 |
| Klebsiellapneumoniae | Cephalosporins(3rdgen) | Ceftazidime | 68 |
| Klebsiellapneumoniae | Cephalosporins(3rdgen) | Ceftriaxone | 155 |
| Klebsiellapneumoniae | Cephalosporins(3rdgen) | Ceftriaxone | 141 |
| Klebsiellapneumoniae | Cephalosporins(3rdgen) | Ceftriaxone | 68 |
| Klebsiellapneumoniae | Cephalosporins(3rdgen) | Cefpodoxime | 70 |
| Klebsiellapneumoniae | Cephalosporins(3rdgen) | Cefpodoxime | 156 |
| Klebsiellapneumoniae | Cephalosporins(3rdgen) | Cefixime | 70 |
| Klebsiellapneumoniae | Cephalosporins(4thgen) | Cefepime | 141 |
| Klebsiellapneumoniae | Cephalosporins(4thgen) | Cefepime | 68 |
| Klebsiellapneumoniae | Cephalosporins(4thgen) | Cefepime | 157 |
| Klebsiellapneumoniae | Cephalosporins(5thgen) | Ceftaroline | 96 |
| Klebsiellapneumoniae | Cephalosporins(5thgen) | Ceftaroline | 98 |
| Klebsiellapneumoniae | Cephalosporins(5thgen) | Ceftaroline | 158 |
| Klebsiellapneumoniae | Fluoroquinolones | Ciprofloxacin | 141 |
| Klebsiellapneumoniae | Fluoroquinolones | Ciprofloxacin | 68 |
| Klebsiellapneumoniae | Fluoroquinolones | Ciprofloxacin | 159 |
| Klebsiellapneumoniae | Fluoroquinolones | Levofloxacin | 141 |
| Klebsiellapneumoniae | Fluoroquinolones | Levofloxacin | 68 |
| Klebsiellapneumoniae | Fluoroquinolones | Levofloxacin | 160 |
| Klebsiellapneumoniae | Fluoroquinolones | Ofloxacin | 58 |

| Klebsiellapneumoniae | Fluoroquinolones | Ofloxacin | 161 |
| --- | --- | --- | --- |
| Klebsiellaspp | Aminoglycosides | Gentamicin | 162 |
| Klebsiellaspp | Aminoglycosides | Gentamicin | 163 |
| Klebsiellaspp | Aminoglycosides | Gentamicin | 164 |
| Klebsiellaspp | Aminoglycosides | Amikacin | 100 |
| Klebsiellaspp | Aminoglycosides | Amikacin | 103 |
| Klebsiellaspp | Aminoglycosides | Amikacin | 165 |
| Legionellapneumophila | Fluoroquinolones | Levofloxacin | 166 |
| Legionellapneumophila | Fluoroquinolones | Moxifloxacin | 166 |
| Legionellapneumophila | Macrolides | Erythromycin | 166 |
| Legionellapneumophila | Macrolides | Azithromycin | 166 |
| Listeriamonocytogenes | Carbapenems | Meropenem | 167 |
| Moraxellacatarrhalis | Penicillins | Penicillin | 168 |
| Moraxellacatarrhalis | Penicillins | Amoxicillin | 169 |
| Moraxellacatarrhalis | Penicillins | Ampicillin | 170 |
| Moraxellacatarrhalis | Penicillins | Ampicillin | 136 |
| Moraxellacatarrhalis | Penicillins | Ampicillin | 171 |
| Moraxellacatarrhalis | Penicillins | Augmentin(amoxicillin/clavulanate) | 171 |
| Moraxellacatarrhalis | Penicillins | Augmentin(amoxicillin/clavulanate) | 123 |
| Moraxellacatarrhalis | Penicillins | Augmentin(amoxicillin/clavulanate) | 172 |
| Moraxellacatarrhalis | Cephalosporins(2ndgen) | Cefuroxime | 169 |
| Moraxellacatarrhalis | Cephalosporins(2ndgen) | Cefuroxime | 173 |
| Moraxellacatarrhalis | Cephalosporins(2ndgen) | Cefuroxime | 136 |
| Moraxellacatarrhalis | Cephalosporins(2ndgen) | Cefaclor | 169 |
| Moraxellacatarrhalis | Cephalosporins(2ndgen) | Cefaclor | 136 |
| Moraxellacatarrhalis | Cephalosporins(2ndgen) | Cefaclor | 174 |
| Moraxellacatarrhalis | Fluoroquinolones | Ciprofloxacin | 123 |
| Moraxellacatarrhalis | Fluoroquinolones | Ciprofloxacin | 172 |

| Moraxellacatarrhalis | Fluoroquinolones | Ciprofloxacin | 169 |
| --- | --- | --- | --- |
| Moraxellacatarrhalis | Fluoroquinolones | Levofloxacin | 126 |
| Moraxellacatarrhalis | Fluoroquinolones | Levofloxacin | 175 |
| Moraxellacatarrhalis | Fluoroquinolones | Levofloxacin | 136 |
| Moraxellacatarrhalis | Fluoroquinolones | Moxifloxacin | 136 |
| Moraxellacatarrhalis | Fluoroquinolones | Moxifloxacin | 176 |
| Moraxellacatarrhalis | Macrolides | Erythromycin | 171 |
| Moraxellacatarrhalis | Macrolides | Erythromycin | 168 |
| Moraxellacatarrhalis | Macrolides | Clarithromycin | 136 |
| Moraxellacatarrhalis | Macrolides | Clarithromycin | 176 |
| Moraxellacatarrhalis | Macrolides | Clarithromycin | 177 |
| Moraxellacatarrhalis | Macrolides | Azithromycin | 169 |
| Moraxellacatarrhalis | Macrolides | Azithromycin | 136 |
| Moraxellacatarrhalis | Macrolides | Azithromycin | 178 |
| Moraxellacatarrhalis | Macrolides | Clindamycin | 169 |
| Moraxellacatarrhalis | Tetracyclines | Doxycyline | 169 |
| Moraxellacatarrhalis | Tetracyclines | Tetracycline | 39 |
| Moraxellacatarrhalis | Tetracyclines | Tetracycline | 126 |
| Moraxellacatarrhalis | Tetracyclines | Tetracycline | 169 |
| Mycoplasmapneumoniae | Macrolides | Erythromycin | 179 |
| Mycoplasmapneumoniae | Macrolides | Erythromycin | 180 |
| Mycoplasmapneumoniae | Macrolides | Erythromycin | 181 |
| Mycoplasmapneumoniae | Macrolides | Azithromycin | 179 |
| Mycoplasmapneumoniae | Macrolides | Azithromycin | 180 |
| Mycoplasmapneumoniae | Macrolides | Azithromycin | 181 |
| Mycoplasmapneumoniae | Tetracyclines | Minocycline | 182 |
| Mycoplasmapneumoniae | Tetracyclines | Tetracycline | 180 |
| Mycoplasmapneumoniae | Tetracyclines | Tetracycline | 182 |

| Neisseriagonorrhoeae | Carbapenems | Ertapenem | 183 |
| --- | --- | --- | --- |
| Neisseriagonorrhoeae | Cephalosporins(2ndgen) | Cefoxitin | 184 |
| Neisseriagonorrhoeae | Cephalosporins(2ndgen) | Cefuroxime | 185 |
| Neisseriagonorrhoeae | Cephalosporins(3rdgen) | Cefotaxime | 186 |
| Neisseriagonorrhoeae | Cephalosporins(3rdgen) | Cefotaxime | 187 |
| Neisseriagonorrhoeae | Cephalosporins(3rdgen) | Cefotaxime | 183 |
| Neisseriagonorrhoeae | Cephalosporins(3rdgen) | Ceftazidime | 188 |
| Neisseriagonorrhoeae | Cephalosporins(3rdgen) | Ceftriaxone | 189 |
| Neisseriagonorrhoeae | Cephalosporins(3rdgen) | Ceftriaxone | 190 |
| Neisseriagonorrhoeae | Cephalosporins(3rdgen) | Ceftriaxone | 191 |
| Neisseriagonorrhoeae | Cephalosporins(3rdgen) | Cefpodoxime | 192 |
| Neisseriagonorrhoeae | Cephalosporins(3rdgen) | Cefpodoxime | 193 |
| Neisseriagonorrhoeae | Cephalosporins(3rdgen) | Cefpodoxime | 194 |
| Neisseriagonorrhoeae | Cephalosporins(3rdgen) | Cefixime | 190 |
| Neisseriagonorrhoeae | Cephalosporins(3rdgen) | Cefixime | 195 |
| Neisseriagonorrhoeae | Cephalosporins(3rdgen) | Cefixime | 196 |
| Neisseriameningitidis | Penicillins | Penicillin | 197 |
| Neisseriameningitidis | Penicillins | Penicillin | 197 |
| Neisseriameningitidis | Penicillins | Penicillin | 198 |
| Neisseriameningitidis | Penicillins | Amoxicillin | 199 |
| Neisseriameningitidis | Penicillins | Ampicillin | 198 |
| Neisseriameningitidis | Penicillins | Ampicillin | 200 |
| Neisseriameningitidis | Carbapenems | Meropenem | 200 |
| Neisseriameningitidis | Cephalosporins(3rdgen) | Cefotaxime | 201 |
| Neisseriameningitidis | Cephalosporins(3rdgen) | Ceftriaxone | 197 |
| Neisseriameningitidis | Cephalosporins(3rdgen) | Ceftriaxone | 197 |
| Neisseriameningitidis | Cephalosporins(3rdgen) | Ceftriaxone | 198 |
| Nocardiaspp | Carbapenems | Imipenem | 202 |

| Nocardiaspp | Carbapenems | Imipenem | 203 |
| --- | --- | --- | --- |
| Nocardiaspp | Carbapenems | Imipenem | 204 |
| Nocardiaspp | Trimethoprim-  sulfamethoxazole | Trimethoprim-sulfamethoxazole | 205 |
| Nocardiaspp | Trimethoprim-  sulfamethoxazole | Trimethoprim-sulfamethoxazole | 202 |
| Nocardiaspp | Trimethoprim-  sulfamethoxazole | Trimethoprim-sulfamethoxazole | 203 |
| Non-typhoidalSalmonella | Cephalosporins(3rdgen) | Cefotaxime | 206 |
| Non-typhoidalSalmonella | Cephalosporins(3rdgen) | Cefotaxime | 207 |
| Non-typhoidalSalmonella | Cephalosporins(3rdgen) | Cefotaxime | 208 |
| Non-typhoidalSalmonella | Cephalosporins(3rdgen) | Ceftazidime | 207 |
| Non-typhoidalSalmonella | Cephalosporins(3rdgen) | Ceftriaxone | 209 |
| Non-typhoidalSalmonella | Cephalosporins(3rdgen) | Ceftriaxone | 210 |
| Non-typhoidalSalmonella | Cephalosporins(3rdgen) | Ceftriaxone | 211 |
| Non-typhoidalSalmonella | Macrolides | Azithromycin | 209 |
| Non-typhoidalSalmonella | Macrolides | Azithromycin | 208 |
| Non-typhoidalSalmonella | Macrolides | Azithromycin | 212 |
| Propiniobacteriumacnes | Penicillins | Penicillin | 213 |
| Propiniobacteriumacnes | Penicillins | Penicillin | 214 |
| Propiniobacteriumacnes | Penicillins | Penicillin | 215 |
| Propiniobacteriumacnes | Penicillins | Ampicillin | 216 |
| Propiniobacteriumacnes | Penicillins | Augmentin(amoxicillin/clavulanate) | 115 |
| Propiniobacteriumacnes | Penicillins | Unasyn(ampicillin/sulbactam) | 216 |
| Propiniobacteriumacnes | Penicillins | Unasyn(ampicillin/sulbactam) | 115 |
| Propiniobacteriumacnes | Carbapenems | Imipenem | 216 |
| Propiniobacteriumacnes | Carbapenems | Imipenem | 115 |
| Propiniobacteriumacnes | Cephalosporins(2ndgen) | Cefoxitin | 216 |
| Propiniobacteriumacnes | Cephalosporins(2ndgen) | Cefoxitin | 115 |
| Propiniobacteriumacnes | Cephalosporins(3rdgen) | Ceftriaxone | 213 |
| Propiniobacteriumacnes | Cephalosporins(3rdgen) | Ceftriaxone | 216 |

| Propiniobacteriumacnes | Cephalosporins(3rdgen) | Ceftriaxone | 217 |
| --- | --- | --- | --- |
| Propiniobacteriumacnes | Macrolides | Erythromycin | 217 |
| Propiniobacteriumacnes | Macrolides | Erythromycin | 216 |
| Propiniobacteriumacnes | Macrolides | Erythromycin | 218 |
| Propiniobacteriumacnes | Macrolides | Clindamycin | 217 |
| Propiniobacteriumacnes | Macrolides | Clindamycin | 213 |
| Propiniobacteriumacnes | Macrolides | Clindamycin | 216 |
| Propiniobacteriumacnes | Nitroimidazoles | Metronidazole | 216 |
| Propiniobacteriumacnes | Tetracyclines | Minocycline | 216 |
| Propiniobacteriumacnes | Tetracyclines | Minocycline | 214 |
| Propiniobacteriumacnes | Tetracyclines | Tetracycline | 217 |
| Propiniobacteriumacnes | Tetracyclines | Tetracycline | 214 |
| Proteusmirabilis | Cephalosporins(1stgen) | Cefazolin | 141 |
| Proteusmirabilis | Cephalosporins(1stgen) | Cefazolin | 219 |
| Proteusmirabilis | Cephalosporins(1stgen) | Cefazolin | 220 |
| Proteusmirabilis | Cephalosporins(1stgen) | Cephalothin | 151 |
| Proteusmirabilis | Cephalosporins(1stgen) | Cephalothin | 152 |
| Proteusmirabilis | Cephalosporins(1stgen) | Cephalothin | 153 |
| Proteusmirabilis | Cephalosporins(1stgen) | Cephalexin | 221 |
| Proteusmirabilis | Cephalosporins(1stgen) | Cephalexin | 153 |
| Proteusmirabilis | Cephalosporins(2ndgen) | Cefoxitin | 141 |
| Proteusmirabilis | Cephalosporins(2ndgen) | Cefoxitin | 151 |
| Proteusmirabilis | Cephalosporins(2ndgen) | Cefoxitin | 152 |
| Proteusmirabilis | Cephalosporins(2ndgen) | Cefuroxime | 141 |
| Proteusmirabilis | Cephalosporins(2ndgen) | Cefuroxime | 222 |
| Proteusmirabilis | Cephalosporins(2ndgen) | Cefuroxime | 223 |
| Proteusmirabilis | Cephalosporins(3rdgen) | Cefotaxime | 224 |
| Proteusmirabilis | Cephalosporins(3rdgen) | Cefotaxime | 151 |

| Proteusmirabilis | Cephalosporins(3rdgen) | Ceftazidime | 225 |
| --- | --- | --- | --- |
| Proteusmirabilis | Cephalosporins(3rdgen) | Ceftazidime | 141 |
| Proteusmirabilis | Cephalosporins(3rdgen) | Ceftazidime | 220 |
| Proteusmirabilis | Cephalosporins(3rdgen) | Ceftriaxone | 141 |
| Proteusmirabilis | Cephalosporins(3rdgen) | Ceftriaxone | 221 |
| Proteusmirabilis | Cephalosporins(3rdgen) | Ceftriaxone | 226 |
| Proteusmirabilis | Cephalosporins(3rdgen) | Cefixime | 227 |
| Proteusmirabilis | Cephalosporins(3rdgen) | Cefixime | 222 |
| Proteusmirabilis | Cephalosporins(4thgen) | Cefepime | 141 |
| Proteusmirabilis | Cephalosporins(4thgen) | Cefepime | 157 |
| Proteusmirabilis | Cephalosporins(4thgen) | Cefepime | 220 |
| Proteusmirabilis | Fluoroquinolones | Ciprofloxacin | 141 |
| Proteusmirabilis | Fluoroquinolones | Ciprofloxacin | 221 |
| Proteusmirabilis | Fluoroquinolones | Ciprofloxacin | 228 |
| Proteusmirabilis | Fluoroquinolones | Levofloxacin | 141 |
| Proteusmirabilis | Fluoroquinolones | Levofloxacin | 160 |
| Proteusmirabilis | Fluoroquinolones | Levofloxacin | 222 |
| Proteusmirabilis | Fluoroquinolones | Moxifloxacin | 152 |
| Proteusmirabilis | Fluoroquinolones | Norfloxacin | 221 |
| Proteusmirabilis | Fluoroquinolones | Norfloxacin | 227 |
| Proteusmirabilis | Fluoroquinolones | Ofloxacin | 226 |
| Proteusspp | Carbapenems | Imipenem | 222 |
| Proteusspp | Carbapenems | Imipenem | 229 |
| Proteusspp | Carbapenems | Imipenem | 220 |
| Proteusspp | Carbapenems | Meropenem | 230 |
| Proteusspp | Carbapenems | Meropenem | 231 |
| Proteusspp | Carbapenems | Meropenem | 232 |
| Pseudomonasaeruginosa | Aminoglycosides | Gentamicin | 233 |

| Pseudomonasaeruginosa | Aminoglycosides | Gentamicin | 141 |
| --- | --- | --- | --- |
| Pseudomonasaeruginosa | Aminoglycosides | Gentamicin | 234 |
| Pseudomonasaeruginosa | Aminoglycosides | Amikacin | 235 |
| Pseudomonasaeruginosa | Aminoglycosides | Amikacin | 225 |
| Pseudomonasaeruginosa | Aminoglycosides | Amikacin | 236 |
| Pseudomonasaeruginosa | Aminoglycosides | Tobramycin | 234 |
| Pseudomonasaeruginosa | Aminoglycosides | Tobramycin | 134 |
| Pseudomonasaeruginosa | Aminoglycosides | Tobramycin | 237 |
| Pseudomonasaeruginosa | Aminoglycosides | Plazomicin | 238 |
| Pseudomonasaeruginosa | Aminoglycosides | Plazomicin | 239 |
| Pseudomonasaeruginosa | Carbapenems | Doripenem | 240 |
| Pseudomonasaeruginosa | Carbapenems | Doripenem | 241 |
| Pseudomonasaeruginosa | Carbapenems | Doripenem | 242 |
| Pseudomonasaeruginosa | Carbapenems | Imipenem | 140 |
| Pseudomonasaeruginosa | Carbapenems | Imipenem | 235 |
| Pseudomonasaeruginosa | Carbapenems | Imipenem | 225 |
| Pseudomonasaeruginosa | Carbapenems | Meropenem | 243 |
| Pseudomonasaeruginosa | Carbapenems | Meropenem | 140 |
| Pseudomonasaeruginosa | Carbapenems | Meropenem | 244 |
| Pseudomonasaeruginosa | Carbapenems | Ertapenem | 245 |
| Pseudomonasaeruginosa | Carbapenems | Ertapenem | 151 |
| Pseudomonasaeruginosa | Carbapenems | Imipenem/Cilastatin | 246 |
| Pseudomonasaeruginosa | Carbapenems | Imipenem/Cilastatin | 146 |
| Pseudomonasaeruginosa | Cephalosporins(3rdgen) | Cefotaxime | 247 |
| Pseudomonasaeruginosa | Cephalosporins(3rdgen) | Cefotaxime | 248 |
| Pseudomonasaeruginosa | Cephalosporins(3rdgen) | Cefotaxime | 249 |
| Pseudomonasaeruginosa | Cephalosporins(3rdgen) | Ceftazidime | 225 |
| Pseudomonasaeruginosa | Cephalosporins(3rdgen) | Ceftazidime | 141 |

| Pseudomonasaeruginosa | Cephalosporins(3rdgen) | Ceftazidime | 234 |
| --- | --- | --- | --- |
| Pseudomonasaeruginosa | Cephalosporins(3rdgen) | Ceftriaxone | 250 |
| Pseudomonasaeruginosa | Cephalosporins(3rdgen) | Ceftriaxone | 251 |
| Pseudomonasaeruginosa | Cephalosporins(3rdgen) | Ceftriaxone | 37 |
| Pseudomonasaeruginosa | Cephalosporins(4thgen) | Cefepime | 234 |
| Pseudomonasaeruginosa | Cephalosporins(4thgen) | Cefepime | 141 |
| Pseudomonasaeruginosa | Cephalosporins(4thgen) | Cefepime | 68 |
| Salmonellatyphi | Carbapenems | Doripenem | 252 |
| Salmonellatyphi | Carbapenems | Imipenem | 253 |
| Salmonellatyphi | Carbapenems | Imipenem | 252 |
| Salmonellatyphi | Carbapenems | Meropenem | 254 |
| Salmonellatyphi | Carbapenems | Meropenem | 221 |
| Salmonellatyphi | Carbapenems | Ertapenem | 252 |
| Salmonellatyphi | Cephalosporins(3rdgen) | Cefotaxime | 255 |
| Salmonellatyphi | Cephalosporins(3rdgen) | Cefotaxime | 256 |
| Salmonellatyphi | Cephalosporins(3rdgen) | Cefotaxime | 257 |
| Salmonellatyphi | Cephalosporins(3rdgen) | Ceftazidime | 254 |
| Salmonellatyphi | Cephalosporins(3rdgen) | Ceftriaxone | 258 |
| Salmonellatyphi | Cephalosporins(3rdgen) | Ceftriaxone | 259 |
| Salmonellatyphi | Cephalosporins(3rdgen) | Ceftriaxone | 254 |
| Salmonellatyphi | Cephalosporins(3rdgen) | Cefpodoxime | 252 |
| Salmonellatyphi | Cephalosporins(3rdgen) | Cefixime | 258 |
| Salmonellatyphi | Cephalosporins(3rdgen) | Cefixime | 260 |
| Salmonellatyphi | Cephalosporins(3rdgen) | Cefixime | 261 |
| Salmonellatyphi | Fluoroquinolones | Ciprofloxacin | 258 |
| Salmonellatyphi | Fluoroquinolones | Ciprofloxacin | 254 |
| Salmonellatyphi | Fluoroquinolones | Ciprofloxacin | 262 |
| Salmonellatyphi | Fluoroquinolones | Levofloxacin | 254 |

| Salmonellatyphi | Fluoroquinolones | Levofloxacin | 263 |
| --- | --- | --- | --- |
| Salmonellatyphi | Fluoroquinolones | Levofloxacin | 264 |
| Salmonellatyphi | Fluoroquinolones | Moxifloxacin | 252 |
| Salmonellatyphi | Fluoroquinolones | Ofloxacin | 254 |
| Salmonellatyphi | Fluoroquinolones | Ofloxacin | 265 |
| Salmonellatyphi | Fluoroquinolones | Ofloxacin | 266 |
| Serratiamarcescens | Carbapenems | Imipenem | 267 |
| Serratiamarcescens | Carbapenems | Imipenem | 268 |
| Serratiamarcescens | Carbapenems | Imipenem | 269 |
| Serratiamarcescens | Carbapenems | Meropenem | 270 |
| Serratiamarcescens | Carbapenems | Meropenem | 271 |
| Serratiamarcescens | Carbapenems | Meropenem | 269 |
| Serratiamarcescens | Cephalosporins(3rdgen) | Cefotaxime | 272 |
| Serratiamarcescens | Cephalosporins(3rdgen) | Cefotaxime | 273 |
| Serratiamarcescens | Cephalosporins(3rdgen) | Cefotaxime | 267 |
| Serratiamarcescens | Cephalosporins(3rdgen) | Ceftazidime | 273 |
| Serratiamarcescens | Cephalosporins(3rdgen) | Ceftazidime | 270 |
| Serratiamarcescens | Cephalosporins(3rdgen) | Ceftriaxone | 273 |
| Serratiamarcescens | Cephalosporins(3rdgen) | Ceftriaxone | 271 |
| Serratiamarcescens | Cephalosporins(3rdgen) | Ceftriaxone | 270 |
| Serratiamarcescens | Fluoroquinolones | Ciprofloxacin | 267 |
| Serratiamarcescens | Fluoroquinolones | Ciprofloxacin | 268 |
| Serratiamarcescens | Fluoroquinolones | Ciprofloxacin | 270 |
| Serratiamarcescens | Fluoroquinolones | Levofloxacin | 268 |
| Serratiamarcescens | Fluoroquinolones | Levofloxacin | 270 |
| Serratiamarcescens | Fluoroquinolones | Levofloxacin | 269 |
| Shigellaspecies | Fluoroquinolones | Ciprofloxacin | 274 |
| Shigellaspecies | Fluoroquinolones | Ciprofloxacin | 275 |

| Shigellaspecies | Fluoroquinolones | Ciprofloxacin | 276 |
| --- | --- | --- | --- |
| Shigellaspecies | Fluoroquinolones | Levofloxacin | 277 |
| Shigellaspecies | Fluoroquinolones | Norfloxacin | 278 |
| Shigellaspecies | Fluoroquinolones | Norfloxacin | 277 |
| Shigellaspecies | Fluoroquinolones | Norfloxacin | 279 |
| Shigellaspecies | Fluoroquinolones | Ofloxacin | 279 |
| Shigellaspecies | Macrolides | Azithromycin | 280 |
| Shigellaspecies | Macrolides | Azithromycin | 281 |
| Shigellaspecies | Macrolides | Azithromycin | 282 |
| Staphylococcusaureus | Carbapenems | Imipenem | 283 |
| Staphylococcusaureus | Carbapenems | Imipenem | 284 |
| Staphylococcusaureus | Carbapenems | Imipenem | 285 |
| Staphylococcusaureus | Cephalosporins(1stgen) | Cefazolin | 286 |
| Staphylococcusaureus | Cephalosporins(1stgen) | Cefazolin | 287 |
| Staphylococcusaureus | Cephalosporins(1stgen) | Cefazolin | 288 |
| Staphylococcusaureus | Cephalosporins(1stgen) | Cephalothin | 289 |
| Staphylococcusaureus | Cephalosporins(1stgen) | Cephalexin | 290 |
| Staphylococcusaureus | Cephalosporins(1stgen) | Cephalexin | 291 |
| Staphylococcusaureus | Cephalosporins(2ndgen) | Cefoxitin | 292 |
| Staphylococcusaureus | Cephalosporins(2ndgen) | Cefoxitin | 293 |
| Staphylococcusaureus | Cephalosporins(2ndgen) | Cefoxitin | 5 |
| Staphylococcusaureus | Cephalosporins(2ndgen) | Cefuroxime | 294 |
| Staphylococcusaureus | Cephalosporins(2ndgen) | Cefuroxime | 295 |
| Staphylococcusaureus | Cephalosporins(2ndgen) | Cefuroxime | 33 |
| Staphylococcusaureus | Cephalosporins(4thgen) | Cefepime | 157 |
| Staphylococcusaureus | Cephalosporins(5thgen) | Ceftaroline | 296 |
| Staphylococcusaureus | Cephalosporins(5thgen) | Ceftaroline | 297 |
| Staphylococcusaureus | Cephalosporins(5thgen) | Ceftaroline | 298 |

| Staphylococcusaureus | Macrolides | Erythromycin | 299 |
| --- | --- | --- | --- |
| Staphylococcusaureus | Macrolides | Erythromycin | 300 |
| Staphylococcusaureus | Macrolides | Erythromycin | 292 |
| Staphylococcusaureus | Macrolides | Clarithromycin | 227 |
| Staphylococcusaureus | Macrolides | Azithromycin | 134 |
| Staphylococcusaureus | Macrolides | Azithromycin | 301 |
| Staphylococcusaureus | Macrolides | Azithromycin | 135 |
| Staphylococcusaureus | Macrolides | Clindamycin | 81 |
| Staphylococcusaureus | Macrolides | Clindamycin | 141 |
| Staphylococcusaureus | Macrolides | Clindamycin | 300 |
| Staphylococcusepidermidis | Carbapenems | Meropenem | 302 |
| Staphylococcusepidermidis | Cephalosporins(1stgen) | Cefazolin | 303 |
| Streptococcusagalactiae | Aminoglycosides | Gentamicin | 304 |
| Streptococcusagalactiae | Aminoglycosides | Amikacin | 304 |
| Streptococcusagalactiae | Cephalosporins(5thgen) | Ceftaroline | 98 |
| Streptococcuspneumoniae | Penicillins | Penicillin | 305 |
| Streptococcuspneumoniae | Penicillins | Penicillin | 306 |
| Streptococcuspneumoniae | Penicillins | Penicillin | 118 |
| Streptococcuspneumoniae | Penicillins | Oxacillin | 307 |
| Streptococcuspneumoniae | Penicillins | Oxacillin | 308 |
| Streptococcuspneumoniae | Penicillins | Oxacillin | 135 |
| Streptococcuspneumoniae | Penicillins | Amoxicillin | 118 |
| Streptococcuspneumoniae | Penicillins | Amoxicillin | 119 |
| Streptococcuspneumoniae | Penicillins | Amoxicillin | 120 |
| Streptococcuspneumoniae | Penicillins | Ampicillin | 118 |
| Streptococcuspneumoniae | Penicillins | Ampicillin | 119 |
| Streptococcuspneumoniae | Penicillins | Ampicillin | 120 |
| Streptococcuspneumoniae | Penicillins | Augmentin(amoxicillin/clavulanate) | 118 |

| Streptococcuspneumoniae | Penicillins | Augmentin(amoxicillin/clavulanate) | 119 |
| --- | --- | --- | --- |
| Streptococcuspneumoniae | Penicillins | Augmentin(amoxicillin/clavulanate) | 120 |
| Streptococcuspneumoniae | Penicillins | Unasyn(ampicillin/sulbactam) | 309 |
| Streptococcuspneumoniae | Penicillins | Zosyn(pipercillin/tazobactam) | 309 |
| Streptococcuspneumoniae | Carbapenems | Doripenem | 1 |
| Streptococcuspneumoniae | Carbapenems | Imipenem | 310 |
| Streptococcuspneumoniae | Carbapenems | Meropenem | 311 |
| Streptococcuspneumoniae | Carbapenems | Meropenem | 310 |
| Streptococcuspneumoniae | Carbapenems | Meropenem | 312 |
| Streptococcuspneumoniae | Cephalosporins(2ndgen) | Cefoxitin | 170 |
| Streptococcuspneumoniae | Cephalosporins(2ndgen) | Cefuroxime | 311 |
| Streptococcuspneumoniae | Cephalosporins(2ndgen) | Cefuroxime | 118 |
| Streptococcuspneumoniae | Cephalosporins(2ndgen) | Cefuroxime | 120 |
| Streptococcuspneumoniae | Cephalosporins(2ndgen) | Cefaclor | 118 |
| Streptococcuspneumoniae | Cephalosporins(2ndgen) | Cefaclor | 119 |
| Streptococcuspneumoniae | Cephalosporins(2ndgen) | Cefaclor | 120 |
| Streptococcuspneumoniae | Cephalosporins(3rdgen) | Cefotaxime | 312 |
| Streptococcuspneumoniae | Cephalosporins(3rdgen) | Cefotaxime | 313 |
| Streptococcuspneumoniae | Cephalosporins(3rdgen) | Cefotaxime | 314 |
| Streptococcuspneumoniae | Cephalosporins(3rdgen) | Cefdinir | 315 |
| Streptococcuspneumoniae | Cephalosporins(3rdgen) | Cefdinir | 129 |
| Streptococcuspneumoniae | Cephalosporins(3rdgen) | Cefdinir | 120 |
| Streptococcuspneumoniae | Cephalosporins(3rdgen) | Ceftriaxone | 118 |
| Streptococcuspneumoniae | Cephalosporins(3rdgen) | Ceftriaxone | 119 |
| Streptococcuspneumoniae | Cephalosporins(3rdgen) | Ceftriaxone | 120 |
| Streptococcuspneumoniae | Cephalosporins(3rdgen) | Cefpodoxime | 129 |
| Streptococcuspneumoniae | Cephalosporins(3rdgen) | Cefpodoxime | 130 |
| Streptococcuspneumoniae | Cephalosporins(3rdgen) | Cefpodoxime | 131 |

| Streptococcuspneumoniae | Cephalosporins(4thgen) | Cefepime | 310 |
| --- | --- | --- | --- |
| Streptococcuspneumoniae | Cephalosporins(4thgen) | Cefepime | 316 |
| Streptococcuspneumoniae | Macrolides | Erythromycin | 317 |
| Streptococcuspneumoniae | Macrolides | Erythromycin | 305 |
| Streptococcuspneumoniae | Macrolides | Erythromycin | 118 |
| Streptococcuspneumoniae | Macrolides | Clarithromycin | 118 |
| Streptococcuspneumoniae | Macrolides | Clarithromycin | 119 |
| Streptococcuspneumoniae | Macrolides | Clarithromycin | 120 |
| Streptococcuspneumoniae | Macrolides | Azithromycin | 311 |
| Streptococcuspneumoniae | Macrolides | Azithromycin | 135 |
| Streptococcuspneumoniae | Macrolides | Azithromycin | 318 |
| Streptococcuspneumoniae | Macrolides | Clindamycin | 305 |
| Streptococcuspneumoniae | Macrolides | Clindamycin | 318 |
| Streptococcuspneumoniae | Macrolides | Clindamycin | 308 |
| Streptococcuspneumoniae | Macrolides | Pristinamycin(Streptogramin) | 319 |
| Streptococcuspyogenes | Penicillins | Penicillin | 123 |
| Streptococcuspyogenes | Penicillins | Penicillin | 320 |
| Streptococcuspyogenes | Penicillins | Penicillin | 321 |
| Streptococcuspyogenes | Penicillins | Amoxicillin | 322 |
| Streptococcuspyogenes | Penicillins | Ampicillin | 309 |
| Streptococcuspyogenes | Penicillins | Ampicillin | 123 |
| Streptococcuspyogenes | Penicillins | Ampicillin | 172 |
| Streptococcuspyogenes | Penicillins | Augmentin(amoxicillin/clavulanate) | 174 |
| Streptococcuspyogenes | Penicillins | Augmentin(amoxicillin/clavulanate) | 323 |
| Streptococcuspyogenes | Penicillins | Unasyn(ampicillin/sulbactam) | 309 |
| Streptococcuspyogenes | Penicillins | Zosyn(pipercillin/tazobactam) | 309 |
| Streptococcuspyogenes | Cephalosporins(2ndgen) | Cefuroxime | 174 |
| Streptococcuspyogenes | Cephalosporins(2ndgen) | Cefuroxime | 323 |

| Streptococcuspyogenes | Cephalosporins(2ndgen) | Cefuroxime | 324 |
| --- | --- | --- | --- |
| Streptococcuspyogenes | Cephalosporins(2ndgen) | Cefaclor | 174 |
| Streptococcuspyogenes | Cephalosporins(2ndgen) | Cefaclor | 324 |
| Streptococcuspyogenes | Cephalosporins(3rdgen) | Cefotaxime | 320 |
| Streptococcuspyogenes | Cephalosporins(3rdgen) | Cefotaxime | 321 |
| Streptococcuspyogenes | Cephalosporins(3rdgen) | Cefotaxime | 325 |
| Streptococcuspyogenes | Cephalosporins(3rdgen) | Ceftriaxone | 322 |
| Streptococcuspyogenes | Cephalosporins(3rdgen) | Ceftriaxone | 320 |
| Streptococcuspyogenes | Cephalosporins(3rdgen) | Ceftriaxone | 321 |
| Streptococcuspyogenes | Cephalosporins(5thgen) | Ceftaroline | 326 |
| Streptococcuspyogenes | Cephalosporins(5thgen) | Ceftaroline | 98 |
| Streptococcuspyogenes | Cephalosporins(5thgen) | Ceftaroline | 98 |
| Streptococcuspyogenes | Macrolides | Erythromycin | 327 |
| Streptococcuspyogenes | Macrolides | Erythromycin | 322 |
| Streptococcuspyogenes | Macrolides | Erythromycin | 328 |
| Streptococcuspyogenes | Macrolides | Azithromycin | 329 |
| Streptococcuspyogenes | Macrolides | Clindamycin | 327 |
| Streptococcuspyogenes | Macrolides | Clindamycin | 330 |
| Streptococcuspyogenes | Macrolides | Clindamycin | 328 |
| Streptococcusviridans | Penicillins | Penicillin | 133 |
| Streptococcusviridans | Penicillins | Penicillin | 331 |
| Streptococcusviridans | Penicillins | Penicillin | 332 |
| Streptococcusviridans | Penicillins | Oxacillin | 331 |
| Streptococcusviridans | Penicillins | Amoxicillin | 333 |
| Streptococcusviridans | Penicillins | Ampicillin | 333 |
| Streptococcusviridans | Penicillins | Ampicillin | 334 |
| Streptococcusviridans | Penicillins | Augmentin(amoxicillin/clavulanate) | 335 |
| Treponemapallidum | Macrolides | Clarithromycin | 336 |

| Treponemapallidum | Macrolides | Azithromycin | 337 |
| --- | --- | --- | --- |
| Treponemapallidum | Macrolides | Azithromycin | 338 |
| Ureaplasmaurealyticum | Fluoroquinolones | Ciprofloxacin | 339 |
| Ureaplasmaurealyticum | Fluoroquinolones | Ciprofloxacin | 5 |
| Ureaplasmaurealyticum | Fluoroquinolones | Ciprofloxacin | 340 |
| Ureaplasmaurealyticum | Fluoroquinolones | Levofloxacin | 236 |
| Ureaplasmaurealyticum | Fluoroquinolones | Levofloxacin | 339 |
| Ureaplasmaurealyticum | Fluoroquinolones | Levofloxacin | 341 |
| Ureaplasmaurealyticum | Fluoroquinolones | Moxifloxacin | 236 |
| Ureaplasmaurealyticum | Fluoroquinolones | Norfloxacin | 342 |
| Ureaplasmaurealyticum | Fluoroquinolones | Ofloxacin | 339 |
| Ureaplasmaurealyticum | Fluoroquinolones | Ofloxacin | 340 |
| Ureaplasmaurealyticum | Fluoroquinolones | Ofloxacin | 5 |
| Ureaplasmaurealyticum | Macrolides | Erythromycin | 236 |
| Ureaplasmaurealyticum | Macrolides | Erythromycin | 339 |
| Ureaplasmaurealyticum | Macrolides | Erythromycin | 343 |
| Ureaplasmaurealyticum | Macrolides | Clarithromycin | 339 |
| Ureaplasmaurealyticum | Macrolides | Clarithromycin | 344 |
| Ureaplasmaurealyticum | Macrolides | Clarithromycin | 343 |
| Ureaplasmaurealyticum | Macrolides | Clarithromycin | 340 |
| Ureaplasmaurealyticum | Macrolides | Azithromycin | 339 |
| Ureaplasmaurealyticum | Macrolides | Azithromycin | 344 |
| Ureaplasmaurealyticum | Macrolides | Azithromycin | 345 |
| Ureaplasmaurealyticum | Macrolides | Clindamycin | 346 |
| Ureaplasmaurealyticum | Macrolides | Pristinamycin(Streptogramin) | 347 |
| Ureaplasmaurealyticum | Macrolides | Pristinamycin(Streptogramin) | 340 |
| Ureaplasmaurealyticum | Tetracyclines | Doxycyline | 347 |
| Ureaplasmaurealyticum | Tetracyclines | Doxycyline | 345 |

| Ureaplasmaurealyticum | Tetracyclines | Doxycyline | 342 |
| --- | --- | --- | --- |
| Ureaplasmaurealyticum | Tetracyclines | Minocycline | 339 |
| Ureaplasmaurealyticum | Tetracyclines | Minocycline | 345 |
| Ureaplasmaurealyticum | Tetracyclines | Minocycline | 342 |
| Ureaplasmaurealyticum | Tetracyclines | Tetracycline | 347 |
| Ureaplasmaurealyticum | Tetracyclines | Tetracycline | 341 |
| Ureaplasmaurealyticum | Tetracyclines | Tetracycline | 348 |
| Vibriocholerae | Tetracyclines | Doxycyline | 349 |
| Vibriocholerae | Tetracyclines | Doxycyline | 350 |
| Vibriocholerae | Tetracyclines | Minocycline | 351 |
| Vibriocholerae | Tetracyclines | Oxytetracycline | 352 |
| Vibriocholerae | Tetracyclines | Tetracycline | 279 |
| Vibriocholerae | Tetracyclines | Tetracycline | 352 |
| Vibriocholerae | Tetracyclines | Tetracycline | 349 |

1. Zhou, M. *et al.* In Vitro Activities of Ceftaroline/Avibactam, Ceftazidime/Avibactam, and Other Comparators Against Pathogens from Various Complicated Infections in China. *Clin. Infect. Dis.* **67**, S206–S216 (2018).
2. Nusrat, T. *et al.* Antibiotic resistance and sensitivity pattern of Metallo-β-Lactamase Producing Gram-Negative Bacilli in ventilator-associated pneumonia in the intensive care unit of a public medical school hospital in Bangladesh. *Hosp. Pract. (1995)* **48**, 128–136 (2020).
3. Amiri, G. *et al.* Determination of imipenem efflux-mediated resistance in Acinetobacte spp., using an efflux pump inhibitor. *Iran. J. Microbiol.* **11**, 368–372 (2019).
4. Dung, V. T. V. *et al.* Antimicrobial susceptibility testing and antibiotic consumption results from 16 hospitals in Viet Nam: The VINARES project 2012-2013. *J. Glob. Antimicrob. Resist.* **18**, 269– 278 (2019).
5. Liu, C. *et al.* Antimicrobial resistance in South Korea: A report from the Korean global antimicrobial resistance surveillance system (Kor-GLASS) for 2017. *J. Infect. Chemother.* **25**, 845– 859 (2019).
6. Saeed, M. *et al.* Acinetobacter Spp: Resistance and therapeutic decisions at the turn of the novel millennium. *Pak. J. Pharm. Sci.* **31**, 2749–2754 (2018).
7. Steininger, C. & Willinger, B. Resistance patterns in clinical isolates of pathogenic Actinomyces species. *J. Antimicrob. Chemother.* **71**, 422–427 (2016).
8. Kutmanova, A., Doganay, M. & Zholdoshev, S. Human anthrax in Kyrgyz Republic: Epidemiology and clinical features. *J. Infect. Public Health* **13**, 1161–1165 (2020).
9. Cobo, F. *et al.* Clinical findings and antimicrobial susceptibility of anaerobic bacteria isolated in bloodstream infections. *Antibiotics* **9**, 1–9 (2020).
10. Maraki, S., Mavromanolaki, V. E., Stafylaki, D. & Kasimati, A. Surveillance of antimicrobial resistance in recent clinical isolates of Gram-negative anaerobic bacteria in a Greek University Hospital. *Anaerobe* **62**, (2020).
11. Cobo, F., Guillot, V. & Navarro-Marí, J. M. Breast abscesses caused by anaerobic microorganisms: Clinical and microbiological characteristics. *Antibiotics* **9**, 1–7 (2020).
12. Veloo, A. C. M., Baas, W. H., Haan, F. J., Coco, J. & Rossen, J. W. Prevalence of antimicrobial resistance genes in Bacteroides spp. and Prevotella spp. Dutch clinical isolates. *Clin. Microbiol. Infect.* **25**, 1156.e9-1156.e13 (2019).
13. Shimura, S. *et al.* Antimicrobial susceptibility surveillance of obligate anaerobic bacteria in the Kinki area. *J. Infect. Chemother.* **25**, 837–844 (2019).
14. Fujita, K. *et al.* Antimicrobial susceptibilities of clinical isolates of the anaerobic bacteria which can cause aspiration pneumonia. *Anaerobe* **57**, 86–89 (2019).
15. Wang, Y. *et al.* Higher prevalence of multi-antimicrobial resistant bacteroides spp. Strains isolated at a tertiary teaching hospital in China. *Infect. Drug Resist.* **13**, 1537–1546 (2020).
16. Wang, J., Deng, Y. & Booth, J. R. （Wang et al., 2019）. *Neuropsychologia* vol. 133 107188 (2019).
17. Li, L. *et al.* High prevalence of macrolide-resistant bordetella pertussis and ptxP1 Genotype, Mainland China, 2014-2016. *Emerging Infectious Diseases* vol. 25 2205–2214 (2019).
18. Zhang, J. S. *et al.* Clinical characteristics, molecular epidemiology and antimicrobial susceptibility of pertussis among children in southern China. *World J. Pediatr.* **16**, 185–192 (2020).
19. Fu, P., Wang, C., Tian, H., Kang, Z. & Zeng, M. Bordetella pertussis Infection in Infants and Young Children in Shanghai, China, 2016-2017: Clinical Features, Genotype Variations of Antigenic Genes and Macrolides Resistance. *Pediatr. Infect. Dis. J.* **38**, 370–376 (2019).
20. Jakubů, V., Zavadilová, J., Fabiánová, K. & Urbášková, P. Trends in the minimum inhibitory concentrations of erythromycin, clarithromycin, azithromycin, ciprofloxacin, and trimethoprim/ Sulfamethoxazole for strains of Bordetella Pertussis isolated in the Czech Republic, 1967–2015. *Cent. Eur. J. Public Health* **25**, 282–286 (2017).
21. Hua, C. Z. *et al.* In vitro activity and clinical efficacy of macrolides, cefoperazone-sulbactam and piperacillin/piperacillin-tazobactam against Bordetella pertussis and the clinical manifestations in pertussis patients due to these isolates: A single-centre study in Zheji. *J. Glob. Antimicrob. Resist.* **18**, 47–51 (2019).
22. Liu, Z. guo *et al.* In vitro antimicrobial susceptibility testing of human Brucella melitensis isolates from Ulanqab of Inner Mongolia, China. *BMC Infect. Dis.* **18**, 1–6 (2018).
23. Torkaman Asadi, F., Hashemi, S. H., Yousef Alikhani, M., Moghimbeigi, A. & Naseri, Z. Clinical and diagnostic aspects of brucellosis and antimicrobial susceptibility of brucella isolates in Hamedan,

Iran. *Jpn. J. Infect. Dis.* **70**, 235–238 (2017).

1. Alamian, S., Dadar, M., Etemadi, A., Afshar, D. & Alamian, M. M. Antimicrobial susceptibility of Brucella spp. isolated from Iranian patients during 2016 to 2018. *Iran. J. Microbiol.* **11**, 363–367 (2019).
2. Lurchachaiwong, W. *et al.* Enteric etiological surveillance in acute diarrhea stool of United States Military Personnel on deployment in Thailand, 2013-2017. *Gut Pathog.* **12**, 1–7 (2020).
3. Elhadidy, M. *et al.* Antimicrobial resistance patterns and molecular resistance markers of Campylobacter jejuni isolates from human diarrheal cases. *PLoS One* **15**, 1–16 (2020).
4. Ilktac, M., Ongen, B., Humphrey, T. J. & Williams, L. K. Molecular and phenotypical investigation of ciprofloxacin resistance among Campylobacter jejuni strains of human origin: high prevalence of resistance in Turkey. *Apmis* **128**, 41–47 (2020).
5. YM, S.-A. *et al.* Fluoroquinolone and macrolide resistance in Campylobacter jejuni isolated from broiler slaughterhouses in southern Brazil. *Avian Pathol.* **45**, 66–72 (2016).
6. Otto, S. J. G. *et al.* Antimicrobial Resistance of Human Campylobacter Species Infections in Saskatchewan, Canada (1999-2006): A Historical Provincial Collection of All Reported Cases. *Foodborne Pathog. Dis.* **17**, 178–186 (2020).
7. Trajkovska-Dokic, E. *et al.* Antimicrobial Susceptibility of Campylobacter isolates in the Capital of North Macedonia . *Prilozi* **40**, 73–80 (2019).
8. Lurchachaiwong, W. *et al.* Determination of azithromycin heteroresistant Campylobacter jejuni in

traveler’s diarrhea. *Gut Pathog.* **11**, 1–5 (2019).

1. Schiaffino, F. *et al.* Antibiotic resistance of Campylobacter species in a pediatric cohort study.

*Antimicrob. Agents Chemother.* **63**, 1–10 (2019).

1. Wang, L. min, Qiao, X. liang, Ai, L., Zhai, J. jing & Wang, X. xia. Isolation of antimicrobial resistant bacteria in upper respiratory tract infections of patients. *3 Biotech* **6**, 1–7 (2016).
2. Takahashi, S. *et al.* Nationwide surveillance of the antimicrobial susceptibility of Chlamydia trachomatis from male urethritis in Japan. *J. Infect. Chemother.* **22**, 581–586 (2016).
3. Maraki, S. *et al.* In vitro susceptibility and resistance phenotypes in contemporary Enterobacter isolates in a university hospital in Crete, Greece. *Future Microbiol.* **12**, 683–693 (2017).
4. Ramalheira, E. & Stone, G. G. Longitudinal analysis of the in vitro activity of ceftazidime/avibactam versus Enterobacteriaceae, 2012–2016. *J. Glob. Antimicrob. Resist.* **19**, 106–115 (2019).
5. Azimi, T., Maham, S., Fallah, F., Azimi, L. & Gholinejad, Z. Evaluating the antimicrobial resistance patterns among major bacterial pathogens isolated from clinical specimens taken from patients in mofid children’s hospital, Tehran, Iran: 2013–2018. *Infect. Drug Resist.* **12**, 2089–2102 (2019).
6. Cheng, L. *et al.* Piperacillin-Tazobactam versus other antibacterial agents for treatment of

bloodstream infections due to AmpC β-Lactamase-producing enterobacteriaceae. *Antimicrob. Agents Chemother.* **61**, 5–7 (2017).

1. Pfaller, M. A., Huband, M. D., Shortridge, D. & Flamm, R. K. Surveillance of omadacycline activity

tested against clinical isolates from the United States and Europe: Report from the SENTRY antimicrobial surveillance program, 2016 to 2018. *Antimicrob. Agents Chemother.* **64**, 1–21

(2020).

1. Praharaj, A. K., Khajuria, A., Kumar, M. & Grover, N. Phenotypic detection and molecular characterization of beta-lactamase genes among Citrobacter species in a tertiary care hospital. *Avicenna J. Med.* **06**, 17–27 (2016).
2. Lew, T. *et al.* Antimicrobial susceptibilities of clostridium difficile isolates from 12 Asia-pacific countries in 2014 and 2015. *Antimicrob. Agents Chemother.* **64**, 1–32 (2020).
3. Cheng, J. W. *et al.* The tcdA-negative and tcdB-positive Clostridium difficile ST81 clone exhibits a high level of resistance to fluoroquinolones: a multi-centre study in Beijing, China. *Int. J. Antimicrob. Agents* **56**, (2020).
4. Jamal, W. Y. & Rotimi, V. O. Surveillance of Antibiotic Resistance among Hospital- And Community-Acquired Toxigenic Clostridium difficile Isolates over 5-Year Period in Kuwait. *PLoS One* **11**, 1–11 (2016).
5. Sárvári, K. P. & Schoblocher, D. The antibiotic susceptibility pattern of gas gangrene-forming Clostridium spp. clinical isolates from South-Eastern Hungary. *Infect. Dis. (Auckl).* **52**, 196–201 (2020).
6. Khademi, F. & Sahebkar, A. The prevalence of antibiotic-resistant Clostridium species in Iran: a meta-analysis. *Pathog. Glob. Health* **113**, 58–66 (2019).
7. Snydman, D. R. *et al.* Evaluation of the in vitro activity of eravacycline against a broad spectrum of recent clinical anaerobic isolates. *Antimicrob. Agents Chemother.* **62**, 1–8 (2018).
8. Zou, J. *et al.* Phenotypic and genotypic correlates of penicillin susceptibility in nontoxigenic corynebacterium diphtheriae, British Columbia, Canada, 2015-2018. *Emerg. Infect. Dis.* **26**, 97– 103 (2020).
9. Husada, D. *et al.* First-line antibiotic susceptibility pattern of toxigenic Corynebacterium diphtheriae in Indonesia. *BMC Infect. Dis.* **19**, 1–11 (2019).
10. Paveenkittiporn, W., Sripakdee, S., Koobkratok, O., Sangkitporn, S. & Kerdsin, A. Molecular epidemiology and antimicrobial susceptibility of outbreak-associated Corynebacterium diphtheriae in Thailand, 2012. *Infect. Genet. Evol.* **75**, 104007 (2019).
11. N, B. *et al.* Microbiological and molecular characterization of Corynebacterium diphtheriae isolated in Algeria between 1992 and 2015. *Clin. Microbiol. Infect.* **22**, 1005.e1-1005.e7 (2016).
12. McCusker, M. P. *et al.* Modulation of antimicrobial resistance in clinical isolates of Enterobacter aerogenes: A strategy combining antibiotics and chemosensitisers. *J. Glob. Antimicrob. Resist.* **16**, 187–198 (2019).
13. Liu, X. J., Lyu, Y., Li, Y., Xue, F. & Liu, J. Trends in antimicrobial resistance against enterobacteriaceae strains isolated from blood: A 10-year epidemiological study in mainland China (2004–2014). *Chin. Med. J. (Engl).* **130**, 2050–2055 (2017).
14. Tansuphasiri, U., Matra, W. & Sangsuk, L. Antimicrobial resistance among Clostridium perfringens isolated from various sources in Thailand. *Southeast Asian J. Trop. Med. Public Health* **36**, 954–

961 (2005).

1. I, M., S, M., S, H., S, S. & P, K. In Vitro Activity of Cefepime-Enmetazobactam against Gram- Negative Isolates Collected from U.S. and European Hospitals during 2014-2015. *Antimicrob. Agents Chemother.* **63**, (2019).
2. Harada, S., Shibue, Y., Aoki, K., Ishii, Y. & Tateda, K. Prevalence of high-level aminoglycoside resistance and genes encoding aminoglycoside-modifying enzymes in enterococcus faecalis and enterococcus faecium isolated in a university hospital in tokyo. *Jpn. J. Infect. Dis.* **73**, 476–480 (2020).
3. NR, B., SBK, S. & B, D. Antibiogram of Urinary Enterococcus Isolates from a Tertiary Care Hospital.

*Infect. Disord. Drug Targets* **21**, 146–150 (2021).

1. Haghi, F., Lohrasbi, V. & Zeighami, H. High incidence of virulence determinants, aminoglycoside and vancomycin resistance in enterococci isolated from hospitalized patients in Northwest Iran. *BMC Infect. Dis.* **19**, 1–10 (2019).
2. Shrestha, L. B., Baral, R., Poudel, P. & Khanal, B. Clinical, etiological and antimicrobial susceptibility profile of pediatric urinary tract infections in a tertiary care hospital of Nepal. *BMC Pediatr.* **19**, 1–8 (2019).
3. Zhang, F., Li, Y., Lv, Y., Zheng, B. & Xue, F. Bacterial susceptibility in bloodstream infections: Results from China Antimicrobial Resistance Surveillance Trial (CARST) Program, 2015–2016. *J. Glob. Antimicrob. Resist.* **17**, 276–282 (2019).
4. Softić, I., Tahirović, H., Di Ciommo, V. & Auriti, C. Bacterial sepsis in neonates: Single centre study in a Neonatal intensive care unit in Bosnia and Herzegovina. *Acta Med. Acad.* **46**, 7–15 (2017).
5. Tang, Y., Yu, F., Hu, Z., Peng, L. & Jiang, Y. Characterization of aerobic vaginitis in late pregnancy in a Chinese population A STROBE-compliant study. *Med. (United States)* **99**, 1–6 (2020).
6. Takesue, Y. *et al.* Antimicrobial susceptibility of common pathogens isolated from postoperative intra-abdominal infections in Japan. *J. Infect. Chemother.* **24**, 330–340 (2018).
7. Hung, P. N. *et al.* Antibiotic resistance profile and diversity of subtypes genes in Escherichia coli causing bloodstream infection in northern Vietnam. *Open Access Maced. J. Med. Sci.* **7**, 4393– 4398 (2019).
8. Sekar, R., Mythreyee, M., Srivani, S. & Amudhan, M. Prevalence of antimicrobial resistance in Escherichia coli and Klebsiella spp. in rural South India. *J. Glob. Antimicrob. Resist.* **5**, 80–85 (2016).
9. Katongole, P., Nalubega, F., Florence, N. C., Asiimwe, B. & Andia, I. Biofilm formation, antimicrobial susceptibility and virulence genes of Uropathogenic Escherichia coli isolated from clinical isolates in Uganda. *BMC Infect. Dis.* **20**, 1–6 (2020).
10. Hossain, A. *et al.* Age and gender-specific antibiotic resistance patterns among Bangladeshi patients with urinary tract infection caused by Escherichia coli. *Heliyon* **6**, e04161 (2020).
11. Ahmadishooli, A. *et al.* Frequency and Antimicrobial Susceptibility Patterns of Diabetic Foot Infection of Patients from Bandar Abbas District, Southern Iran. *J. Pathog.* **2020**, 1–10 (2020).
12. Tuon, F. F., Cieslinski, J., Rodrigues, S. da S., Serra, F. B. & Paula, M. D. N. de. Evaluation of in vitro

activity of ceftolozane–tazobactam against recent clinical bacterial isolates from Brazil – the EM200 study. *Brazilian J. Infect. Dis.* **24**, 96–103 (2020).

1. Koguchi, D., Murakami, Y., Ikeda, M., Dobashi, M. & Ishii, J. Cefaclor as a first-line treatment for acute uncomplicated cystitis: A retrospective single-center study. *BMC Urol.* **20**, 1–7 (2020).
2. Keshi, L. *et al.* Analysis of drug resistance of extended-spectrum betalactamases-producing Escherichia coli and Klebsiella pneumoniae in children with urinary tract infection. *Saudi Med. J.* **40**, 1111–1115 (2019).
3. Aguilar-Santelises, M. *et al.* Clinical isolates of Escherichia coli are resistant both to antibiotics and organotin compounds. *Folia Microbiol. (Praha).* **65**, 87–94 (2020).
4. M, R. & R, R. Antibiotic resistance, virulence factors and genotyping of Uropathogenic Escherichia coli strains. *Antimicrob. Resist. Infect. Control* **7**, (2018).
5. Zhang, S. X. *et al.* Antibiotic resistance and molecular characterization of diarrheagenic Escherichia coli and non-typhoidal Salmonella strains isolated from infections in Southwest China. *Infect. Dis. Poverty* **7**, 1–11 (2018).
6. Tohamy, S. T., Aboshanab, K. M., El-Mahallawy, H. A., El-Ansary, M. R. & Afifi, S. S. Prevalence of multidrug-resistant Gram-negative pathogens isolated from febrile neutropenic cancer patients with bloodstream infections in Egypt and new synergistic antibiotic combinations. *Infect. Drug Resist.* **11**, 791–803 (2018).
7. Djordjević, Z., Folić, M., Ninković, V., Vasiljević, D. & Janković, S. Antimicrobial susceptibility among urinary Escherichia coli isolates from female outpatients: Age-related differences. *Cent. Eur. J. Public Health* **27**, 245–250 (2019).
8. Bryce, A., Costelloe, C., Wootton, M., Butler, C. C. & Hay, A. D. Comparison of risk factors for, and prevalence of, antibiotic resistance in contaminating and pathogenic urinary Escherichia coli in children in primary care: Prospective cohort study. *J. Antimicrob. Chemother.* **73**, 1359–1367 (2018).
9. Neupane, S., Pant, N. D., Khatiwada, S., Chaudhary, R. & Banjara, M. R. Correlation between biofilm formation and resistance toward different commonly used antibiotics along with extended spectrum beta lactamase production in uropathogenic Escherichia coli isolated from the patients suspected of urinary tract infections visit. *Antimicrob. Resist. Infect. Control* **5**, 1–5 (2016).
10. Miyazaki, M. *et al.* Change in the Antimicrobial Resistance Profile of Extended-Spectrum β- Lactamase-Producing Escherichia coli . *J. Clin. Med. Res.* **11**, 635–641 (2019).
11. Horie, A. *et al.* Increased community-acquired upper urinary tract infections caused by extended- spectrum beta-lactamase-producing Escherichia coli in children and the efficacy of flomoxef and cefmetazole. *Clin. Exp. Nephrol.* **23**, 1306–1314 (2019).
12. Mao, T., Zhai, H., Duan, G. & Yang, H. Patterns of drug-resistant bacteria in a general hospital, China, 2011-2016. *Polish J. Microbiol.* **68**, 225–232 (2019).
13. Singh, A. K. *et al.* Prevalence of antibiotic resistance in commensal Escherichia coli among the children in rural hill communities of northeast India. *PLoS One* **13**, 1–15 (2018).
14. Odongo, I., Ssemambo, R. & Kungu, J. M. Prevalence of Escherichia Coli and Its Antimicrobial Susceptibility Profiles among Patients with UTI at Mulago Hospital, Kampala, Uganda. *Interdiscip. Perspect. Infect. Dis.* **2020**, (2020).
15. Rodrigues, R. S. *et al.* Antibiotic resistance and biofilm formation in children with Enteropathogenic Escherichia coli (EPEC) in Brazilian Amazon. *J. Infect. Dev. Ctries.* **13**, 698–705 (2019).
16. A, S. *et al.* Characteristics of febrile urinary tract infections in older male adults. *BMC Geriatr.* **19**, (2019).
17. Younas, M. *et al.* Characterization of enteropathogenic Escherichia coli of clinical origin from the pediatric population in Pakistan. *Trans. R. Soc. Trop. Med. Hyg.* **110**, 414–420 (2016).
18. Khalil, U. *et al.* Phenotypic and genotypic characterization of enteroaggregative Escherichia coli isolates from pediatric population in Pakistan. *Apmis* **124**, 872–880 (2016).
19. Kim, B. *et al.* Molecular Epidemiology of Ciprofloxacin-Resistant Escherichia coli Isolated from Community-Acquired Urinary Tract Infections in Korea. *Infect. Chemother.* **52**, 194–203 (2020).
20. Kayastha, K. *et al.* Extended-Spectrum β-Lactamase-Producing Escherichia coli and Klebsiella Species in Pediatric Patients Visiting International Friendship Children’s Hospital, Kathmandu, Nepal . *Infect. Dis. Res. Treat.* **13**, 117863372090979 (2020).
21. Ko, W. C. & Stone, G. G. In vitro activity of ceftazidime-avibactam and comparators against Gram- negative bacterial isolates collected in the Asia-Pacific region as part of the INFORM program (2015-2017). *Ann. Clin. Microbiol. Antimicrob.* **19**, 1–12 (2020).
22. Zavala-Cerna, M. G. *et al.* The Clinical Significance of High Antimicrobial Resistance in Community-Acquired Urinary Tract Infections. *Can. J. Infect. Dis. Med. Microbiol.* **2020**, 1–7 (2020).
23. Sun, J. *et al.* Eight-year surveillance of uropathogenic escherichia coli in southwest china. *Infect. Drug Resist.* **13**, 1197–1202 (2020).
24. K, S., E, S. & KS, B. Prevalence and antibiotic resistance pattern of extended-spectrum beta- lactamase-producing Escherichia coli in clinical specimens. *J. Res. Med. Sci.* **24**, (2019).
25. A, G. *et al.* Prevalence and characterization of beta-lactamase-producing Escherichia coli isolates from a tertiary care hospital in India. *J. Lab. Physicians* **11**, 123–127 (2019).
26. H, M., S, G., O, Z., H, H. & MY, A. Identification of Quinolone and Colistin Resistance Genes in Escherichia Coli Strains Isolated from Mucosal Samples of Patients with Colorectal Cancer and Healthy Subjects. *Recent Pat. Antiinfect. Drug Discov.* **15**, 30–40 (2020).
27. Paskeh, M. D. A., Moghaddam, M. J. M. & Salehi, Z. Prevalence of plasmid-encoded carbapenemases in multi-drug resistant Escherichia coli from patients with urinary tract infection in northern Iran. *Iran. J. Basic Med. Sci.* **23**, 586–593 (2020).
28. Denisuik, A. J. *et al.* Antimicrobial-resistant pathogens in Canadian ICUs: Results of the CANWARD 2007 to 2016 study. *J. Antimicrob. Chemother.* **74**, 645–653 (2019).
29. Pfaller, M. A. *et al.* Ceftaroline activity tested against bacterial isolates causing community- acquired respiratory tract infections and skin and skin structure infections in pediatric patients

from United States hospitals: 2012-2014. *Pediatr. Infect. Dis. J.* **36**, 486–491 (2016).

1. Karlowsky, J. A. *et al.* In vitro activity of Ceftaroline against bacterial pathogens isolated from patients with skin and soft tissue and respiratory tract infections in African and Middle Eastern countries: AWARE global surveillance program 2012–2014. *Diagn. Microbiol. Infect. Dis.* **86**, 194– 199 (2016).
2. Ghaddar, N. *et al.* Phenotypic and Genotypic Characterization of Extended-Spectrum Beta- Lactamases Produced by Escherichia coli Colonizing Pregnant Women. *Infect. Dis. Obstet. Gynecol.* **2020**, (2020).
3. Chibelean, C. B. *et al.* A clinical perspective on the antimicrobial resistance spectrum of uropathogens in a Romanian male population. *Microorganisms* **8**, 1–15 (2020).
4. Sierra-Díaz, E., Hernández-Ríos, C. J. & Bravo-Cuellar, A. Antibiotic resistance: Microbiological profile of urinary tract infections in Mexico. *Cir. y Cir. (English Ed.* **87**, 176–182 (2019).
5. Norouzian, H. *et al.* The relationship between phylogenetic groups and antibiotic susceptibility patterns of Escherichia coli strains isolated from feces and urine of patients with acute or recurrent urinary tract infection. *Iran. J. Microbiol.* **11**, 478–487 (2019).
6. Sorsa, A., Früh, J., Stötter, L. & Abdissa, S. Blood culture result profile and antimicrobial resistance pattern: A report from neonatal intensive care unit (NICU), Asella teaching and referral hospital, Asella, south East Ethiopia. *Antimicrob. Resist. Infect. Control* **8**, 6–11 (2019).
7. Shah, C., Baral, R., Bartaula, B. & Shrestha, L. B. Virulence factors of uropathogenic Escherichia coli (UPEC) and correlation with antimicrobial resistance. *BMC Microbiol.* **19**, 1–6 (2019).
8. Plantamura, J. *et al.* Molecular epidemiological of extended-spectrum β-lactamase producing Escherichia coli isolated in Djibouti. *J. Infect. Dev. Ctries.* **13**, 753–758 (2019).
9. Moharana, S. S. *et al.* Etiology of childhood diarrhoea among under five children and molecular analysis of antibiotic resistance in isolated enteric bacterial pathogens from a tertiary care hospital, eastern odisha, india. *BMC Infect. Dis.* **19**, 1–9 (2019).
10. Nand, D. *et al.* Global mortality from firearms, 1990-2016. *JAMA - J. Am. Med. Assoc.* **320**, 792– 814 (2018).
11. Caspar, Y., Hennebique, A. & Maurin, M. Antibiotic susceptibility of Francisella tularensis subsp. holarctica strains isolated from tularaemia patients in France between 2006 and 2016. *J. Antimicrob. Chemother.* **73**, 687–691 (2018).
12. Tomaso, H., Hotzel, H., Otto, P., Myrtennäs, K. & Forsman, M. Antibiotic susceptibility in vitro of Francisella tularensis subsp. holarctica isolates from Germany. *J. Antimicrob. Chemother.* **72**, 2539–2543 (2017).
13. Li, H. *et al.* Molecular epidemiology and antimicrobial resistance of group a streptococcus recovered from patients in Beijing, China. *BMC Infect. Dis.* **20**, 1–9 (2020).
14. MAB, P., LA, C., LK, R. & SL, H. Susceptibility of bacterial vaginosis (BV)-associated bacteria to secnidazole compared to metronidazole, tinidazole and clindamycin. *Anaerobe* **47**, 115–119 (2017).
15. de Souza, D. M. K. *et al.* Antimicrobial susceptibility and vaginolysin in Gardnerella vaginalis from

healthy and bacterial vaginosis diagnosed women. *J. Infect. Dev. Ctries.* **10**, 913–919 (2016).

1. Byun, J. H., Kim, M., Lee, Y., Lee, K. & Chong, Y. Antimicrobial susceptibility patterns of anaerobic bacterial clinical isolates from 2014 to 2016, including recently named or renamed species. *Ann. Lab. Med.* **39**, 190–199 (2019).
2. Badri, M., Nilson, B., Ragnarsson, S., Senneby, E. & Rasmussen, M. Clinical and microbiological features of bacteraemia with Gram-positive anaerobic cocci: a population-based retrospective study. *Clin. Microbiol. Infect.* **25**, 760.e1-760.e6 (2019).
3. Demirci, M. *et al.* A Retrospective Analysis of Anaerobic Bacteria Isolated in 236 Cases of Pleural Empyema and their Prevalence of Antimicrobial Resistance in Turkey. *Clin. Lab.* **64**, 1269–1277 (2018).
4. Tan, T. Y., Ng, L. S. Y., Kwang, L. L., Rao, S. & Eng, L. C. Clinical characteristics and antimicrobial susceptibilities of anaerobic bacteremia in an acute care hospital. *Anaerobe* **43**, 69–74 (2017).
5. Hama, M. K. *et al.* Pediatric Bacterial Meningitis Surveillance in Niger: Increased Importance of Neisseria meningitidis Serogroup C, and a Decrease in Streptococcus pneumoniae Following 13- Valent Pneumococcal Conjugate Vaccine Introduction. *Clin. Infect. Dis. An Off. Publ. Infect. Dis. Soc. Am.* **69**, S133 (2019).
6. Torumkuney, D. *et al.* Results from the Survey of Antibiotic Resistance (SOAR) 2015-17 in Turkey: Data based on CLSI, EUCAST (dose-specific) and pharmacokinetic/pharmacodynamic (PK/PD) breakpoints. *J. Antimicrob. Chemother.* **75**, I88–I99 (2020).
7. Torumkuney, D. *et al.* Results from the Survey of Antibiotic Resistance (SOAR) 2016-18 in Vietnam, Cambodia, Singapore and the Philippines: Data based on CLSI, EUCAST (dose-specific) and pharmacokinetic/pharmacodynamic (PK/PD) breakpoints. *J. Antimicrob. Chemother.* **75**, I19– I42 (2020).
8. Torumkuney, D., Bratus, E., Yuvko, O., Pertseva, T. & Morrissey, I. Results from the Survey of Antibiotic Resistance (SOAR) 2016-17 in Ukraine: Data based on CLSI, EUCAST (dose-specific) and pharmacokinetic/pharmacodynamic (PK/PD) breakpoints. *J. Antimicrob. Chemother.* **75**, I100– I111 (2020).
9. Li, X. X. *et al.* Molecular Epidemiology and Antimicrobial Resistance of Haemophilus influenzae in Adult Patients in Shanghai, China. *Front. Public Heal.* **8**, 1–8 (2020).
10. HJ, W. *et al.* Antibiotic Resistance Profiles of Haemophilus influenzae Isolates from Children in 2016: A Multicenter Study in China. *Can. J. Infect. Dis. Med. Microbiol. = J. Can. des Mal. Infect. la Microbiol. medicale* **2019**, (2019).
11. Yanagihara, K. *et al.* Nationwide surveillance of bacterial respiratory pathogens conducted by the surveillance committee of Japanese Society of Chemotherapy, the Japanese Association for Infectious Diseases, and the Japanese Society for clinical microbiology in 2014: General v. *J. Infect. Chemother.* **25**, 657–668 (2019).
12. Omoding, D. & Bazira, J. Isolation and Antibiotic Susceptibility Testing of Haemophilus influenzae from Nasopharynx of Children under Five Years Attending Maternal and Child Health Clinic in Mbarara Regional Referral Hospital. *Can. J. Infect. Dis. Med. Microbiol.* **2019**, (2019).
13. Jiang, H. *et al.* Prevalence and antibiotic resistance profiles of cerebrospinal fluid pathogens in

children with acute bacterial meningitis in Yunnan province, China, 2012-2015. *PLoS One* **12**, 2012–2015 (2017).

1. Sader, H. S., Flamm, R. K., Streit, J. M., Carvalhaes, C. G. & Mendes, R. E. Antimicrobial activity of ceftaroline and comparator agents tested against organisms isolated from patients with community-acquired bacterial pneumonia in Europe, Asia, and Latin America. *Int. J. Infect. Dis.* **77**, 82–86 (2018).
2. Li, J. P. *et al.* Epidemiological Features and Antibiotic Resistance Patterns of Haemophilus influenzae Originating from Respiratory Tract and Vaginal Specimens in Pediatric Patients. *J. Pediatr. Adolesc. Gynecol.* **30**, 626–631 (2017).
3. Kılıç, H., Akyol, S., Parkan, Ö. M., Dinç, G. & Sav, H. Molecular characterization and antibiotic

susceptibility of Haemophilus influenzae clinical isolates. *Le Infez. Med.* **1**, 27–32 (2017).

1. Torumkuney, D., Anwar, S., Nizamuddin, S., Malik, N. & Morrissey, I. Results from the Survey of Antibiotic Resistance (SOAR) 2015-17 in Pakistan: Data based on CLSI, EUCAST (dose-specific) and pharmacokinetic/pharmacodynamic (PK/PD) breakpoints. *J. Antimicrob. Chemother.* **75**, I76–I87 (2020).
2. Torumkuney, D. *et al.* Results from the Survey of Antibiotic Resistance (SOAR) 2015-17 in Latin America (Argentina, Chile and Costa Rica): Data based on CLSI, EUCAST (dose-specific) and pharmacokinetic/pharmacodynamic (PK/PD) breakpoints. *J. Antimicrob. Chemother.* **75**, I43–I59 (2020).
3. Torumkuney, D. *et al.* Results from the Survey of Antibiotic Resistance (SOAR) 2015-17 in the Middle East (Kuwait, Lebanon and Saudi Arabia): Data based on CLSI, EUCAST (dose-specific) and pharmacokinetic/pharmacodynamic (PK/PD) breakpoints. *J. Antimicrob. Chemother.* **75**, I60–I75 (2020).
4. Biedenbach, D. J. *et al.* In Vitro Activity of Oral Antimicrobial Agents against Pathogens Associated with Community-Acquired Upper Respiratory Tract and Urinary Tract Infections: A Five Country Surveillance Study. *Infect. Dis. Ther.* **5**, 139–153 (2016).
5. Pfaller, M. A. *et al.* Antimicrobial activity of ceftobiprole and comparator agents when tested against contemporary Gram-positive and -negative organisms collected from Europe (2015). *Diagn. Microbiol. Infect. Dis.* **91**, 77–84 (2018).
6. Asbell, P. A., Sanfilippo, C. M., Sahm, D. F. & Decory, H. H. Trends in Antibiotic Resistance among Ocular Microorganisms in the United States from 2009 to 2018. *JAMA Ophthalmol.* **138**, 439–450 (2020).
7. PA, A. & HH, D. Antibiotic resistance among bacterial conjunctival pathogens collected in the Antibiotic Resistance Monitoring in Ocular Microorganisms (ARMOR) surveillance study. *PLoS One* **13**, (2018).
8. Zhang, Y. *et al.* Antimicrobial susceptibility of Streptococcus pneumoniae, Haemophilus influenzae and Moraxella catarrhalis isolated from community-acquired respiratory tract infections in China: Results from the CARTIPS Antimicrobial Surveillance Program. *J. Glob. Antimicrob. Resist.* **5**, 36–41 (2016).
9. Pettigrew, M. M. *et al.* Effect of fluoroquinolones and macrolides on eradication and resistance of Haemophilus influenzae in chronic obstructive pulmonary disease. *Antimicrob. Agents*

*Chemother.* **60**, 4151–4158 (2016).

1. Maddi, S. *et al.* Ampicillin resistance in Haemophilus influenzae from COPD patients in the UK.

*Int. J. COPD* 1507–1518 (2017).

1. Karlowsky, J. A. *et al.* In vitro activity of ceftaroline against bacterial pathogens isolated from skin and soft tissue infections in Europe, Russia and Turkey in 2012: Results from the Assessing Worldwide Antimicrobial Resistance Evaluation (AWARE) surveillance programme. *J. Antimicrob. Chemother.* **71**, 162–169 (2016).
2. Yi, H. *et al.* Increased antimicrobial resistance among sputum pathogens from patients with hyperglycemia. *Infect. Drug Resist.* **13**, 1723–1733 (2020).
3. Yang, W. & Ji, X. Analysis of the microbial species, antimicrobial sensitivity and drug resistance in 2652 patients of nursing hospital. *Heliyon* **6**, (2020).
4. Lu, B. *et al.* Molecular Characteristics of Klebsiella pneumoniae Isolates From Outpatients in Sentinel Hospitals, Beijing, China, 2010–2019. *Front. Cell. Infect. Microbiol.* **10**, (2020).
5. Tan, K. *et al.* Prevalence of the carbapenem-heteroresistant phenotype among ESBL-producing Escherichia coli and Klebsiella pneumoniae clinical isolates. *J. Antimicrob. Chemother.* **75**, 1506– 1512 (2020).
6. Li, G. *et al.* A 7-year surveillance of the drug resistance in Klebsiella pneumoniae from a primary health care center. *Ann. Clin. Microbiol. Antimicrob.* **18**, 1–7 (2019).
7. Liao, C. H. *et al.* Antimicrobial activities of ceftazidime-avibactam, ceftolozane-tazobactam, and other agents against escherichia coli, klebsiella pneumoniae, and pseudomonas aeruginosa isolated from intensive care units in Taiwan: Results from the surveillance of multicen. *Infect. Drug Resist.* **12**, 545–552 (2019).
8. Lob, S. H. *et al.* Vitro activity of imipenem-relebactam against gram-negative eskape pathogens isolated by clinical laboratories in the United States in 2015 (results from the smart global surveillance program). *Antimicrob. Agents Chemother.* **61**, 1–9 (2017).
9. Chen, Y. *et al.* Pathogenic characteristics of nosocomial infections in patients with cerebrovascular diseases and characteristics and treatment of pathogenic bacteria in different seasons. *J. Infect. Public Health* **13**, 800–805 (2020).
10. Uc-Cachón, A. H., Gracida-Osorno, C., Luna-Chi, I. G., Jiménez-Guillermo, J. G. & Molina-Salinas,

G. M. High prevalence of antimicrobial resistance among gram-negative isolated bacilli in intensive care units at a tertiary-care hospital in Yucatán Mexico. *Med.* **55**, (2019).

1. Alsanie, W. F. Molecular diversity and profile analysis of virulence-associated genes in some Klebsiella pneumoniae isolates. *Pract. Lab. Med.* **19**, e00152 (2020).
2. Gunduz, S. & Uludağ Altun, H. Antibiotic resistance patterns of urinary tract pathogens in Turkish

children. *Glob. Heal. Res. Policy* **3**, 1–5 (2018).

1. Alamri, A. *et al.* Trend analysis of bacterial uropathogens and their susceptibility pattern: A 4-year (2013-2016) study from Aseer region, Saudi Arabia. *Urol. Ann.* **10**, 41–46 (2018).
2. Mohammed, M. A., Alnour, T. M. S., Shakurfo, O. M. & Aburass, M. M. Prevalence and antimicrobial resistance pattern of bacterial strains isolated from patients with urinary tract

infection in Messalata Central Hospital, Libya. *Asian Pac. J. Trop. Med.* **9**, 771–776 (2016).

1. Jalali, H. K. *et al.* Antagonistic Activity of Nocardia brasiliensis PTCC 1422 Against Isolated Enterobacteriaceae from Urinary Tract Infections. *Probiotics Antimicrob. Proteins* **8**, 41–45 (2016).
2. Liu, B. *et al.* Antimicrobial resistance and risk factors for mortality of pneumonia caused by klebsiella pneumoniae among diabetics: A retrospective study conducted in Shanghai, China. *Infect. Drug Resist.* **12**, 1089–1098 (2019).
3. Weldu, Y. *et al.* Neonatal septicemia at intensive care unit, Ayder Comprehensive Specialized Hospital, Tigray, North Ethiopia: Bacteriological profile, drug susceptibility pattern, and associated factors. *PLoS One* **15**, 1–14 (2020).
4. Shahraki-Zahedani, S., Rigi, S., Bokaeian, M., Ansari-Moghaddam, A. & Moghadampour, M. First report of TEM-104-, SHV-99-, SHV-108-, and SHV-110-producing Klebsiella pneumoniae from Iran. *Rev. Soc. Bras. Med. Trop.* **49**, 441–445 (2016).
5. Cheng, J. W. *et al.* In vitro Activity of a New Fourth-Generation Cephalosporin, Cefoselis, Against Clinically Important Bacterial Pathogens in China. *Front. Microbiol.* **11**, 1–7 (2020).
6. Sader, H. S., Flamm, R. K., Mendes, R. E., Farrell, D. J. & Jones, R. N. Antimicrobial activities of ceftaroline and comparator agents against bacterial organisms causing bacteremia in patients with skin and skin structure infections in U.S. medical centers, 2008 to 2014. *Antimicrob. Agents Chemother.* **60**, 2558–2563 (2016).
7. Delarampour, A., Ghalehnoo, Z. R., Khademi, F. & Vaez, H. Antibiotic resistance patterns and prevalence of class I, II and III integrons among clinical isolates of klebsiella pneumoniae. *Infez. Med.* **28**, 64–69 (2020).
8. Kobayashi, K. *et al.* The third national Japanese antimicrobial susceptibility pattern surveillance program: Bacterial isolates from complicated urinary tract infection patients. *J. Infect. Chemother.* **26**, 418–428 (2020).
9. Onanuga, A. & Selekere, T. L. Virulence and antimicrobial resistance of common urinary bacteria from asymptomatic students of Niger Delta University, Amassoma, Bayelsa State, Nigeria. *J. Pharm. Bioallied Sci.* **8**, 29–33 (2016).
10. Akinpelu, S., Ajayi, A., Smith, S. I. & Adeleye, A. I. Efflux pump activity, biofilm formation and antibiotic resistance profile of Klebsiella spp. isolated from clinical samples at Lagos University Teaching Hospital. *BMC Res. Notes* **13**, 1–5 (2020).
11. Hope, D. *et al.* Antimicrobial resistance in pathogenic aerobic bacteria causing surgical site infections in Mbarara regional referral hospital, Southwestern Uganda. *Sci. Rep.* **9**, 1–10 (2019).
12. Kiponza, R., Balandya, B., Majigo, M. V. & Matee, M. Laboratory confirmed puerperal sepsis in a national referral hospital in Tanzania: Etiological agents and their susceptibility to commonly prescribed antibiotics. *BMC Infect. Dis.* **19**, 1–7 (2019).
13. Maham, S., Fallah, F., Gholinejad, Z., Seifi, A. & Hoseini-Alfatemi, S. M. Bacterial etiology and antibiotic resistance pattern of pediatric bloodstream infections: A multicenter based study in Tehran, Iran. *Ann. di Ig. Med. Prev. e di Comunita* **30**, 337–345 (2018).
14. Jia, X. *et al.* Antibiotic Resistance and Azithromycin Resistance Mechanism of Legionella pneumophila Serogroup 1 in China. *Antimicrob. Agents Chemother.* **63**, 1–11 (2019).
15. Noll, M., Kleta, S. & Al Dahouk, S. Antibiotic susceptibility of 259 Listeria monocytogenes strains isolated from food, food-processing plants and human samples in Germany. *J. Infect. Public Health* **11**, 572–577 (2018).
16. W, M. *et al.* Pharyngeal colonization and drug resistance profiles of Morraxella catarrrhalis, Streptococcus pneumoniae, Staphylococcus aureus, and Haemophilus influenzae among HIV infected children attending ART Clinic of Felegehiwot Referral Hospital, Ethiopia. *PLoS One* **13**, (2018).
17. Du, Y. *et al.* Multilocus sequence typing-based analysis of Moraxella catarrhalis population structure reveals clonal spreading of drug-resistant strains isolated from childhood pneumonia. *Infect. Genet. Evol.* **56**, 117–124 (2017).
18. Sampane-Donkor, E., Badoe, E. V., Annan, J. A. & Nii-Trebi, N. I. Colonisation of antibiotic- resistant bacteria in a cohort of HIV infected children in Ghana. *Pan Afr. Med. J.* **26**, 1–7 (2017).
19. Shi, W. *et al.* β-Lactamase production and antibiotic susceptibility pattern of Moraxella catarrhalis isolates collected from two county hospitals in China. *BMC Microbiol.* **18**, 1–6 (2018).
20. Yanagihara, K. *et al.* Nationwide surveillance of bacterial respiratory pathogens conducted by the surveillance committee of Japanese Society of Chemotherapy, the Japanese Association for Infectious Diseases, and the Japanese Society for Clinical Microbiology in 2012: General v. *J. Infect. Chemother.* **23**, 587–597 (2017).
21. Olzowy, B., Kresken, M., Havel, M., Hafner, D. & Körber-Irrgang, B. Antimicrobial susceptibility of bacterial isolates from patients presenting with ear, nose and throat (ENT) infections in the German community healthcare setting. *Eur. J. Clin. Microbiol. Infect. Dis.* **36**, 1685–1690 (2017).
22. Soyletir, G. *et al.* Results from the Survey of Antibiotic Resistance (SOAR) 2011-13 in Turkey. *J. Antimicrob. Chemother.* **71**, i71–i83 (2016).
23. Flamm, R. K., Rhomberg, P. R., Huband, M. D. & Farrell, D. J. In vitro activity of delafloxacin tested against isolates of Streptococcus pneumoniae, Haemophilus influenzae, and Moraxella catarrhalis. *Antimicrob. Agents Chemother.* **60**, 6381–6385 (2016).
24. Farrell, D. J., Flamm, R. K., Sader, H. S. & Jones, R. N. Results from the Solithromycin International Surveillance Program (2014). *Antimicrob. Agents Chemother.* **60**, 3662–3668 (2016).
25. Flamm, R. K., Rhomberg, P. R. & Sader, H. S. In vitro activity of the novel lactone ketolide nafithromycin (WCK 4873) against contemporary clinical bacteria from a global surveillance program. *Antimicrob. Agents Chemother.* **61**, 1–8 (2017).
26. Hu, F. *et al.* Results from the Survey of Antibiotic Resistance (SOAR) 2009-11 and 2013-14 in China. *J. Antimicrob. Chemother.* **71**, i33–i43 (2016).
27. Wang, N., Zhou, Y., Zhang, H. & Liu, Y. In vitro activities of acetylmidecamycin and other antimicrobials against human macrolide-resistant Mycoplasma pneumoniae isolates. *J. Antimicrob. Chemother.* **75**, 1513–1517 (2021).
28. Zhao, F. *et al.* Antimicrobial susceptibility and molecular characteristics of Mycoplasma

pneumoniae isolates across different regions of China. *Antimicrob. Resist. Infect. Control* **8**, 1–8 (2019).

1. Zhao, F. *et al.* Antimicrobial susceptibility and genotyping of Mycoplasma pneumoniae isolates in Beijing, China, from 2014 to 2016. *Antimicrob. Resist. Infect. Control* **8**, 1–8 (2019).
2. Yin, Y. D. *et al.* Macrolide-resistant Mycoplasma pneumoniae prevalence and clinical aspects in adult patients with community-acquired pneumonia in China: A prospective multicenter surveillance study. *J. Thorac. Dis.* **9**, 3774–3781 (2017).
3. Ryan, L. *et al.* Antimicrobial resistance and molecular epidemiology using whole-genome sequencing of Neisseria gonorrhoeae in Ireland, 2014–2016: focus on extended-spectrum cephalosporins and azithromycin. *Eur. J. Clin. Microbiol. Infect. Dis.* **37**, 1661–1672 (2018).
4. Ali, S., Sewunet, T., Sahlemariam, Z. & Kibru, G. Neisseria gonorrhoeae among suspects of sexually transmitted infection in Gambella hospital, Ethiopia: Risk factors and drug resistance. *BMC Res. Notes* **9**, 1–8 (2016).
5. Bailey, A. L. *et al.* Genotypic and Phenotypic Characterization of Antimicrobial Resistance in Neisseria gonorrhoeae: a Cross-Sectional Study of Isolates Recovered from Routine Urine Cultures in a High-Incidence Setting. *mSphere* **4**, (2019).
6. Visser, M., Van Westreenen, M., Van Bergen, J. & Van Benthem, B. H. B. Low gonorrhoea antimicrobial resistance and culture positivity rates in general practice: A pilot study. *Sex. Transm. Infect.* **96**, 220–222 (2020).
7. Hofstraat, S. H. I., Götz, H. M., van Dam, A. P., van der Sande, M. A. B. & van Benthem, B. H. B. Trends and determinants of antimicrobial susceptibility of Neisseria gonorrhoeae in the Netherlands, 2007 to 2015. *Eurosurveillance* **23**, 1–13 (2018).
8. Tribuddharat, C. *et al.* Gonococcal antimicrobial susceptibility and the prevalence of blaTEM-1 and blaTEM-135 genes in Neisseria gonorrhoeae isolates from Thailand. *Jpn. J. Infect. Dis.* **70**, 213–215 (2017).
9. Workneh, M. *et al.* Antimicrobial Resistance of Neisseria Gonorrhoeae in a Newly Implemented Surveillance Program in Uganda: Surveillance Report. *JMIR Public Heal. Surveill.* **6**, 1–19 (2020).
10. Pinto, M. *et al.* Fifteen years of a nationwide culture collection of Neisseria gonorrhoeae antimicrobial resistance in Portugal. *Eur. J. Clin. Microbiol. Infect. Dis.* **39**, 1761–1770 (2020).
11. Fentaw, S. *et al.* Antimicrobial susceptibility profile of Gonococcal isolates obtained from men presenting with urethral discharge in Addis Ababa, Ethiopia: Implications for national syndromic treatment guideline. *PLoS One* **15**, 1–10 (2020).
12. Tshokey, T. *et al.* Antibiotic resistance in neisseria gonorrhoea and treatment outcomes of gonococcal urethritis suspected patients in two large hospitals in Bhutan, 2015. *PLoS One* **13**, 1– 11 (2018).
13. Liu, Y. H., Huang, Y. T., Liao, C. H. & Hsueh, P. R. Antimicrobial susceptibilities and molecular typing of neisseria gonorrhoeae isolates at a medical centre in Taiwan, 2001–2013 with an emphasis on high rate of azithromycin resistance among the isolates. *Int. J. Antimicrob. Agents* **51**, 768–774 (2018).
14. Cheng, C. W., Li, L. H., Su, C. Y., Li, S. Y. & Yen, M. Y. Changes in the six most common sequence types of Neisseria gonorrhoeae, including ST4378, identified by surveillance of antimicrobial resistance in northern Taiwan from 2006 to 2013. *J. Microbiol. Immunol. Infect.* **49**, 708–716 (2016).
15. Boiko, I. *et al.* Genomic epidemiology and antimicrobial resistance determinants of Neisseria gonorrhoeae isolates from Ukraine, 2013–2018. *Apmis* **128**, 465–475 (2020).
16. Lan, P. T. *et al.* Genomic analysis and antimicrobial resistance of Neisseria gonorrhoeae isolates from Vietnam in 2011 and 2015-16. *J. Antimicrob. Chemother.* **75**, 1432–1438 (2021).
17. Lahra, M. M. & Hogan, T. R. Australian Meningococcal Surveillance Programme annual report, 2019. *Commun. Dis. Intell.* **44**, (2020).
18. Gorla, M. C., Pinhata, J. M. W., Dias, U. J., de Moraes, C. & Lemos, A. P. Surveillance of antimicrobial resistance in neisseria meningitidis strains isolated from invasive cases in Brazil from 2009 to 2016. *J. Med. Microbiol.* **67**, 750–756 (2018).
19. Saguer, A., Smaoui, H., Taha, M.-K. & Kechrid, A. Characterization of invasive Neisseria meningit idis strains isolated at the Children’s Hospital of Tunis, Tunisia. *East. Mediterr. Heal. J.* **22**, 343– 349 (2016).
20. Lee, H., Seo, Y., Kim, K. H., Lee, K. & Choe, K. W. Prevalence and serogroup changes of Neisseria meningitidis in South Korea. *Sci. Rep.* **8**, 2010–2016 (2018).
21. Kharkhal, H. N. & Titov, L. P. Serogroup Diversity and Antibiotic Susceptibility of Neisseria meningitidis: Meningococcus Infection Monitoring in Belarus. *Acta Microbiol. Immunol. Hung.* **66**, 443–457 (2019).
22. Tan, Y. E., Chen, S. C. A. & Halliday, C. L. Antimicrobial susceptibility profiles and species distribution of medically relevant Nocardia species: Results from a large tertiary laboratory in Australia. *J. Glob. Antimicrob. Resist.* **20**, 110–117 (2020).
23. Huang, L. *et al.* Clinical features, identification, antimicrobial resistance patterns of Nocardia species in China: 2009–2017. *Diagn. Microbiol. Infect. Dis.* **94**, 165–172 (2019).
24. Farooqui, F., Irfan, S., Shakoor, S. & Zafar, A. Antimicrobial susceptibility and clinical characteristics of Nocardia isolates from a tertiary care centre diagnostic laboratory in Pakistan. *J. Glob. Antimicrob. Resist.* **15**, 219–221 (2018).
25. Yi, M. *et al.* Species distribution and antibiotic susceptibility of Nocardia isolates from Yantai, China. *Infect. Drug Resist.* **12**, 3653–3661 (2019).
26. Zhan, Z. *et al.* Molecular epidemiology and antimicrobial resistance of invasive non-typhoidal salmonella in China, 2007–2016. *Infect. Drug Resist.* **12**, 2885–2897 (2019).
27. Andoh, L. A. *et al.* Prevalence and characterization of Salmonella among humans in Ghana. *Trop. Med. Health* **45**, 1–11 (2017).
28. Harb, A., O’Dea, M., Hanan, Z. K., Abraham, S. & Habib, I. Prevalence, risk factors and antimicrobial resistance of Salmonella diarrhoeal infection among children in Thi-Qar Governorate, Iraq. *Epidemiol. Infect.* **145**, 3486–3496 (2017).
29. Tack, B. *et al.* Non-typhoidal salmonella bloodstream infections in Kisantu, DR Congo: Emergence

of O5-negative salmonella typhimurium and extensive drug resistance. *PLoS Negl. Trop. Dis.* **14**, 1–22 (2020).

1. Luvsansharav, U. O. *et al.* Exploration of risk factors for ceftriaxone resistance in invasive non- typhoidal Salmonella infections in western Kenya. *PLoS One* **15**, 1–13 (2020).
2. Pragasam, A. K. *et al.* Real-Time Cytotoxicity Assays. *Indian J. Med. Microbiol.* 1–5 (2020) doi:10.4103/ijmm.IJMM.
3. Mason, C. J. *et al.* Antibiotic resistance in Campylobacter and other diarrheal pathogens isolated from US military personnel deployed to Thailand in 2002-2004: A case-control study. *Trop. Dis. Travel Med. Vaccines* **3**, 1–7 (2017).
4. Couture, A. *et al.* Calcium sulphate mixed with antibiotics does not decrease efficacy against Cutibacterium acnes (formerly Propionibacterium acnes), in vitro study. *J. Orthop.* **19**, 138–142 (2020).
5. Biswal, I. *et al.* In vitro antimicrobial susceptibility patterns of propionibacterium acnes isolated from patients with acne vulgaris. *J. Infect. Dev. Ctries.* **10**, 1140–1145 (2016).
6. Wright, T. E., Boyle, K. K., Duquin, T. R. & Crane, J. K. Propionibacterium acnes Susceptibility and Correlation with Hemolytic Phenotype . *Infect. Dis. Res. Treat.* **9**, IDRT.S40539 (2016).
7. Zhang, N., Yuan, R., Xin, K. Z., Lu, Z. & Ma, Y. Antimicrobial Susceptibility, Biotypes and Phylotypes of Clinical Cutibacterium (Formerly Propionibacterium) acnes Strains Isolated from Acne Patients: An Observational Study. *Dermatol. Ther. (Heidelb).* **9**, 735–746 (2019).
8. Broly, M. *et al.* Propionibacterium/Cutibacterium species–related positive samples, identification, clinical and resistance features: a 10-year survey in a French hospital. *Eur. J. Clin. Microbiol. Infect. Dis.* **39**, 1357–1364 (2020).
9. Ma, Y., Zhang, N., Wu, S., Huang, H. & Cao, Y. Antimicrobial activity of topical agents against Propionibacterium acnes: an in vitro study of clinical isolates from a hospital in Shanghai, China. *Front. Med.* **10**, 517–521 (2016).
10. Wu, C. T., Lee, H. Y., Chen, C. L., Tuan, P. L. & Chiu, C. H. High prevalence and antimicrobial resistance of urinary tract infection isolates in febrile young children without localizing signs in Taiwan. *J. Microbiol. Immunol. Infect.* **49**, 243–248 (2016).
11. Lin, M. F. *et al.* Antimicrobial Susceptibility and Molecular Epidemiology of Proteus mirabilis Isolates from Three Hospitals in Northern Taiwan. *Microb. Drug Resist.* **25**, 1338–1346 (2019).
12. Hussein, E. I. *et al.* Assessment of Pathogenic Potential, Virulent Genes Profile, and Antibiotic Susceptibility of Proteus mirabilis from Urinary Tract Infection. *Int. J. Microbiol.* **2020**, (2020).
13. Rafalskiy, V. *et al.* Distribution and antibiotic resistance profile of key Gram-negative bacteria that cause community-onset urinary tract infections in the Russian Federation: RESOURCE multicentre surveillance 2017 study. *J. Glob. Antimicrob. Resist.* **21**, 188–194 (2020).
14. Mezzatesta, M. L. *et al.* In vitro activity of fosfomycin trometamol and other oral antibiotics against multidrug-resistant uropathogens. *Int. J. Antimicrob. Agents* **49**, 763–766 (2017).
15. Boudjemaa, H. *et al.* Molecular drivers of emerging multidrug resistance in Proteus mirabilis clinical isolates from Algeria. *J. Glob. Antimicrob. Resist.* **18**, 249–256 (2019).
16. Stone, G. G., Seifert, H. & Nord, C. E. In vitro activity of ceftazidime-avibactam against Gram- negative isolates collected in 18 European countries, 2015–2017. *Int. J. Antimicrob. Agents* **56**, 106045 (2020).
17. Pulcini, C., Clerc-Urmes, I., Attinsounon, C. A., Fougnot, S. & Thilly, N. Antibiotic resistance of Enterobacteriaceae causing urinary tract infections in elderly patients living in the community and in the nursing home: A retrospective observational study. *J. Antimicrob. Chemother.* **74**, 775– 781 (2019).
18. Molla, R., Tiruneh, M., Abebe, W. & Moges, F. Bacterial profile and antimicrobial susceptibility patterns in chronic suppurative otitis media at the University of Gondar Comprehensive Specialized Hospital, Northwest Ethiopia. *BMC Res. Notes* **12**, 1–6 (2019).
19. Honsbeek, M. *et al.* Low antimicrobial resistance in general practice patients in Rotterdam, the city with the largest proportion of immigrants in the Netherlands. *Eur. J. Clin. Microbiol. Infect. Dis.* **39**, 929–935 (2020).
20. Bashir, A. *et al.* Superbugs-related prolonged admissions in three tertiary hospitals, Kano State, Nigeria. *Pan Afr. Med. J.* **32**, 166 (2019).
21. Hubab, M., Ullah, O., Hayat, A., Ur Rehman, M. & Sultana, N. Antibiotic susceptibility profile of bacterial isolates from post-surgical wounds of patients in tertiary care hospitals of Peshawar, Pakistan. *J. Pak. Med. Assoc.* **68**, 1517–1520 (2018).
22. Fazeli, H., Moghim, S. & Zare, D. Antimicrobial Resistance Pattern and Spectrum of Multiple-drug- resistant Enterobacteriaceae in Iranian Hospitalized Patients with Cancer. *Adv. Biomed. Res.* **7**, 69 (2018).
23. Partina, I. *et al.* Surveillance of antimicrobial susceptibility of Enterobacteriaceae pathogens isolated from intensive care units and surgical units in Russia. *Jpn. J. Antibiot.* **69**, 41–51 (2016).
24. Kolar, M. *et al.* Antibiotic resistance in nosocomial bacteria isolated from infected wounds of hospitalized patients in czech republic. *Antibiotics* **9**, 1–8 (2020).
25. Ahmed, N. *et al.* Evaluation of antibiotic resistance and virulence genes among clinical isolates of Pseudomonas aeruginosa from cancer patients. *Asian Pacific J. Cancer Prev.* **21**, 1333–1338 (2020).
26. Goh, T. C. *et al.* Clinical and bacteriological profile of diabetic foot infections in a tertiary care. *J. Foot Ankle Res.* **13**, 1–8 (2020).
27. Yang, T. *et al.* Antimicrobial resistance in clinical Ureaplasma spp. And Mycoplasma hominis and Structural Mechanisms Underlying Quinolone Resistance. *Antimicrob. Agents Chemother.* **64**, 1– 11 (2020).
28. Ekkelenkamp, M. B. *et al.* Susceptibility of Pseudomonas aeruginosa Recovered from. *Antimicrob. Agents Chemother.* 1–7 (2020).
29. Castanheira, M. *et al.* Activity of plazomicin compared with other aminoglycosides against isolates from European and adjacent countries, including Enterobacteriaceae molecularly characterized for aminoglycoside-modifying enzymes and other resistance mechanisms. *J. Antimicrob. Chemother.* **73**, 3346–3354 (2018).
30. Castanheira, M. *et al.* In Vitro Activity of Plazomicin against Gram-Positive Isolates Collected from U . S . Hospitals and Carbapenem-Resistant Enterobacteriaceae and Isolates Carrying Carbapenemase Genes. *Antimicrob. Agents Chemother.* **62**, 1–8 (2018).
31. Sader, H. S., Castanheira, M., Streit, J. M. & Flamm, R. K. Frequency of occurrence and antimicrobial susceptibility of bacteria isolated from patients hospitalized with bloodstream infections in United States medical centers (2015–2017). *Diagn. Microbiol. Infect. Dis.* **95**, 114850 (2019).
32. Liew, S. M., Rajasekaram, G., Puthucheary, S. D. A. & Chua, K. H. Antimicrobial susceptibility and virulence genes of clinical and environmental isolates of Pseudomonas aeruginosa. *PeerJ* **2019**, 1–19 (2019).
33. Micaëlo, M. *et al.* Interpreting carbapenem susceptibility testing results for Pseudomonas aeruginosa. *Med. Mal. Infect.* **48**, 365–371 (2018).
34. Alnimr, A. M. & Alamri, A. M. Antimicrobial activity of cephalosporin–beta-lactamase inhibitor combinations against drug-susceptible and drug-resistant Pseudomonas aeruginosa strains. *J. Taibah Univ. Med. Sci.* **15**, 203–210 (2020).
35. Emami, A. *et al.* Three year study of infection profile and antimicrobial resistance pattern from burn patients in southwest iran. *Infect. Drug Resist.* **13**, 1499–1506 (2020).
36. Devrim, F. *et al.* The emerging resistance in nosocomial urinary tract infections: From the pediatrics perspective. *Mediterr. J. Hematol. Infect. Dis.* **10**, 3–7 (2018).
37. Karlowsky, J. A. *et al.* In vitro activity of imipenem-relebactam against clinical isolates of gram- negative bacilli isolated in hospital laboratories in the United States as part of the SMART 2016 program. *Antimicrob. Agents Chemother.* **62**, 1–11 (2018).
38. Zhang, X., Lu, Q., Liu, T., Li, Z. & Cai, W. Bacterial resistance trends among intraoperative bone culture of chronic osteomyelitis in an affiliated hospital of South China for twelve years. *BMC Infect. Dis.* **19**, 1–8 (2019).
39. Ibrahim, M. E. High antimicrobial resistant rates among gram-negative pathogens in intensive care units a retrospective study at a tertiary care hospital in southwest saudi arabia. *Saudi Med. J.* **39**, 1035–1043 (2018).
40. Roshani-Asl, P., Rashidi, N., Shokoohizadeh, L. & Zarei, J. Relationship among antibiotic resistance, biofilm formation and lasB gene in pseudomonas aeruginosa isolated from burn patients. *Clin. Lab.* **64**, 1477–1484 (2018).
41. Xu, J., Du, Q., Shu, Y., Ji, J. & Dai, C. Bacteriological Profile of Chronic Suppurative Otitis Media and Antibiotic Susceptibility in a Tertiary Care Hospital in Shanghai, China. *Ear, Nose Throat J.* 0–5 (2020) doi:10.1177/0145561320923823.
42. Abebe, M., Tadesse, S., Meseret, G. & Derbie, A. Type of bacterial isolates and antimicrobial resistance profile from different clinical samples at a Referral Hospital, Northwest Ethiopia: Five years data analysis. *BMC Res. Notes* **12**, 1–6 (2019).
43. Malik, N. & Ahmed, M. In vitro effect of new antibiotics against clinical isolates of Salmonella Typhi. *J. Coll. Physicians Surg. Pakistan* **26**, 288–292 (2016).
44. Singh, L. & Cariappa, M. P. Blood culture isolates and antibiogram of Salmonella: Experience of a tertiary care hospital. *Med. J. Armed Forces India* **72**, 281–284 (2016).
45. Ohanu, M. E., Iroezindu, M. O., Maduakor, U., Onodugo, O. D. & Gugnani, H. C. Typhoid fever among febrile Nigerian patients: Prevalence, diagnostic performance of the widal test and antibiotic multi-drug resistance. *Malawi Med. J.* **31**, 184–192 (2019).
46. Lv, D., Zhang, D. & Song, Q. Expansion of salmonella typhi clonal lineages with ampicillin resistance and reduced ciprofloxacin susceptibility in Eastern China. *Infect. Drug Resist.* **12**, 2215– 2221 (2019).
47. Mutai, W. C., Muigai, A. W. T., Waiyaki, P. & Kariuki, S. Multi-drug resistant Salmonella enterica serovar Typhi isolates with reduced susceptibility to ciprofloxacin in Kenya. *BMC Microbiol.* **18**, 4– 8 (2018).
48. Behl, P., Gupta, V., Sachdev, A., Guglani, V. & Chander, J. Patterns in antimicrobial susceptibility of Salmonellae isolated at a tertiary care hospital in northern India. *Indian J. Med. Res.* **145**, 124– 128 (2017).
49. Katiyar, A. *et al.* Genomic profiling of antimicrobial resistance genes in clinical isolates of Salmonella Typhi from patients infected with Typhoid fever in India. *Sci. Rep.* **10**, 1–15 (2020).
50. Browne, A. J. *et al.* Drug-resistant enteric fever worldwide, 1990 to 2018: A systematic review and meta-analysis. *BMC Med.* **18**, 1–22 (2020).
51. Patil, N. & Mule, P. Sensitivity pattern of salmonella typhi and paratyphi a isolates to chloramphenicol and other anti-typhoid drugs: An in vitro study. *Infect. Drug Resist.* **12**, 3217– 3225 (2019).
52. Khatun, H. *et al.* Clinical profile, antibiotic susceptibility pattern of bacterial isolates and factors associated with complications in culture-proven typhoid patients admitted to an urban hospital in Bangladesh. *Trop. Med. Int. Heal.* **23**, 359–366 (2018).
53. Aneley Getahun, S. *et al.* A retrospective study of patients with blood culture-confirmed typhoid fever in Fiji during 2014-2015: Epidemiology, clinical features, treatment and outcome. *Trans. R. Soc. Trop. Med. Hyg.* **113**, 764–770 (2019).
54. Khanal, P. R. *et al.* Renaissance of Conventional First-Line Antibiotics in Salmonella enterica Clinical Isolates: Assessment of MICs for Therapeutic Antimicrobials in Enteric Fever Cases from Nepal. *Biomed Res. Int.* **2017**, (2017).
55. Hardjo Lugito, N. P. & Cucunawangsih. Antimicrobial resistance of salmonella enterica serovars Typhi and paratyphi isolates from a general Hospital in Karawaci, Tangerang, Indonesia: A five- year review. *Int. J. Microbiol.* **2017**, (2017).
56. Bhetwal, A., Maharjan, A., Khanal, P. R. & Parajuli, N. P. Enteric Fever Caused by Salmonella enterica Serovars with Reduced Susceptibility of Fluoroquinolones at a Community Based Teaching Hospital of Nepal. *Int. J. Microbiol.* **2017**, (2017).
57. Ali, A. *et al.* Pattern of antimicrobial drug resistance of salmonella typhi and paratyphi a in a teaching hospital in Islamabad. *J. Pak. Med. Assoc.* **67**, 375–379 (2017).
58. Parente, T. M. A. L., Rebouças, E. de L., dos Santos, V. C. V., Barbosa, F. C. B. & Zanin, I. C. J.

Serratia marcescens resistance profile and its susceptibility to photodynamic antimicrobial chemotherapy. *Photodiagnosis Photodyn. Ther.* **14**, 185–190 (2016).

1. Soltani, J., Poorabbas, B., Miri, N. & Mardaneh, J. Health care associated infections, antibiotic resistance and clinical outcome: A surveillance study from Sanandaj, Iran. *World J. Clin. Cases* **4**, 63 (2016).
2. Rodloff, A. C. & Dowzicky, M. J. Antimicrobial Susceptibility among European Gram-Negative and Gram-Positive Isolates Collected as Part of the Tigecycline Evaluation and Surveillance Trial (2004-2014). *Chemotherapy* **62**, 1–11 (2016).
3. Ghaith, D. M. *et al.* First reported nosocomial outbreak of Serratia marcescens harboring blaIMP- 4 and blaVIM-2 in a neonatal intensive care unit in Cairo, Egypt. *Infect. Drug Resist.* **11**, 2211– 2217 (2018).
4. Seifert, H., Blondeau, J. & Dowzicky, M. J. In vitro activity of tigecycline and comparators (2014– 2016) among key WHO ‘priority pathogens’ and longitudinal assessment (2004–2016) of antimicrobial resistance: a report from the T.E.S.T. study. *Int. J. Antimicrob. Agents* **52**, 474–484 (2018).
5. Mamishi, S. *et al.* Antibiotic resistance and genotyping of gram-positive bacteria causing hospital- acquired infection in patients referring to children’s medical center. *Infect. Drug Resist.* **12**, 3719– 3726 (2019).
6. Şimşek, M. Determination of the Antibiotic Resistance Rates of. *Niger. J. Clin. Pract.* **22(1)**, 125– 130 (2019).
7. Teimourpour, R., Babapour, B., Esmaelizad, M., Arzanlou, M. & Peeri-Doghaheh, H. Molecular characterization of quinolone resistant shigella spp. Isolates from patients in ardabil, Iran. *Iran. J. Microbiol.* **11**, 496–501 (2019).
8. Assefa, A. & Girma, M. Prevalence and antimicrobial susceptibility patterns of Salmonella and Shigella isolates among children aged below five years with diarrhea attending Robe General Hospital and Goba Referral Hospital, South East Ethiopia. *Trop. Dis. Travel Med. Vaccines* **5**, 1–11 (2019).
9. Getie, M., Abebe, W. & Tessema, B. Prevalence of enteric bacteria and their antimicrobial susceptibility patterns among food handlers in Gondar town, Northwest Ethiopia. *Antimicrob. Resist. Infect. Control* **8**, 4–9 (2019).
10. Shahsavan, S., Owlia, P., Rastegar Lari, A., Bakhshi, B. & Nobakht, M. Investigation of efflux- mediated tetracycline resistance in Shigella isolates using the inhibitor and real time polymerase chain reaction method. *Iran. J. Pathol.* **12**, 53–61 (2017).
11. Abbasi, E., Abtahi, H., van Belkum, A. & Ghaznavi-Rad, E. Multidrug-resistant shigella infection in pediatric patients with diarrhea from central Iran. *Infect. Drug Resist.* **12**, 1535–1544 (2019).
12. Sah, S. K. *et al.* Burden of Shigella spp and Vibrio spp, and their antibiotic sensitivity pattern in the patients with acute gastroenteritis in tertiary care hospital in Nepal. *BMC Res. Notes* **12**, 1–5 (2019).
13. Nikfar, R., Shamsizadeh, A., Darbor, M., Khaghani, S. & Moghaddam, M. A study of prevalence of shigella species and antimicrobial resistance patterns in paediatric medical center, Ahvaz, Iran.

*Iran. J. Microbiol.* **9**, 277–282 (2017).

1. Aminshahidi, M., Arastehfar, A., Pouladfar, G., Arman, E. & Fani, F. Diarrheagenic Escherichia coli and Shigella with High Rate of Extended-Spectrum Beta-Lactamase Production: Two Predominant Etiological Agents of Acute Diarrhea in Shiraz, Iran. *Microb. Drug Resist.* **23**, 1037–1044 (2017).
2. Nüesch-Inderbinen, M. *et al.* Shigella antimicrobial drug resistance mechanisms, 2004–2014.

*Emerg. Infect. Dis.* **22**, 1083–1085 (2016).

1. Ngalani, O. J. T., Marbou, W. J. T., Mbaveng, A. T. & Kuete, V. Resistance Profiles of Staphylococcus aureus and Immunological Status in Pregnant Women at Bafang, West Region of Cameroon: A Cross-Sectional Study. *Cureus* **12**, (2020).
2. Sharma, D., Preston, S. E. & Hage, R. Emerging Antibiotic Resistance to Bacterial Isolates from Human Urinary Tract Infections in Grenada. *Cureus* **11**, (2019).
3. Tehrani, F. H. E., Moradi, M. & Ghorbani, N. Bacterial etiology and antibiotic resistance patterns in neonatal sepsis in tehran during 2006-2014. *Iran. J. Pathol.* **12**, 356–361 (2017).
4. Motamedi, H., Asghari, B., Roshanaei, G. & Arabestani, M. R. Identification of hemolysin encoding genes and their association with antimicrobial resistance pattern among clinical isolates of coagulase-negative Staphylococci. *Adv. Biomed. Res.* **7**, (2018).
5. Naimi, H. M., Rasekh, H., Noori, A. Z. & Bahaduri, M. A. Determination of antimicrobial susceptibility patterns in Staphylococcus aureus strains recovered from patients at two main health facilities in Kabul, Afghanistan. *BMC Infect. Dis.* **17**, 1–7 (2017).
6. Zhanel, G. G. *et al.* 42936 pathogens from Canadian hospitals: 10 years of results (2007-16) from the CANWARD surveillance study. *J. Antimicrob. Chemother.* **74**, iv5–iv21 (2019).
7. Tadesse, S. *et al.* Antimicrobial resistance profile of Staphylococcus aureus isolated from patients with infection at Tikur Anbessa Specialized Hospital, Addis Ababa, Ethiopia. *BMC Pharmacol. Toxicol.* **19**, 1–8 (2018).
8. Chinnambedu, R. S. *et al.* Changing antibiotic resistance profile of Staphylococcus aureus isolated from HIV patients (2012–2017) in Southern India. *J. Infect. Public Health* **13**, 75–79 (2020).
9. Tolera, M., Abate, D., Dheresa, M. & Marami, D. Bacterial Nosocomial Infections and Antimicrobial Susceptibility Pattern among Patients Admitted at Hiwot Fana Specialized University Hospital, Eastern Ethiopia. *Adv. Med.* **2018**, 1–7 (2018).
10. Appiah, V. A. *et al.* Staphylococcus aureus nasal colonization among children with sickle cell disease at the children’s hospital, accra: Prevalence, risk factors, and antibiotic resistance. *Pathogens* **9**, (2020).
11. Schulte, R. H. & Munson, E. Staphylococcus aureus resistance patterns in wisconsin: 2018 surveillance of Wisconsin organisms for trends in antimicrobial resistance and epidemiology (swotare) program report. *Clin. Med. Res.* **17**, 72–81 (2019).
12. Tian, L., Zhang, Z. & Sun, Z. Y. Pathogen Analysis of Central Nervous System Infections in a Chinese Teaching Hospital from 2012–2018: A Laboratory-based Retrospective Study. *Curr. Med. Sci.* **39**, 449–454 (2019).
13. Pius, S. *et al.* Neonatal septicaemia, bacterial isolates and antibiogram sensitivity in Maiduguri

North-Eastern Nigeria. *Niger. Postgrad. Med. J.* **23**, 146–151 (2016).

1. Gu, F. *et al.* Antimicrobial Resistance and Molecular Epidemiology of Staphylococcus aureus Causing Bloodstream Infections at Ruijin Hospital in Shanghai from 2013 to 2018. *Sci. Rep.* **10**, 1– 8 (2020).
2. C, V.-E. *et al.* Study of susceptibility to antibiotics and molecular characterization of high virulence Staphylococcus aureus strains isolated from a rural hospital in Ethiopia. *PLoS One* **15**, (2020).
3. Zhang, Z., Chen, M., Yu, Y., Liu, B. & Liu, Y. In vitro activity of ceftaroline and comparators against staphylococcus aureus isolates: Results from 6 years of the ATLAS program (2012 to 2017). *Infect. Drug Resist.* **12**, 3349–3358 (2019).
4. Ai, X. *et al.* Prevalence, Characterization, and Drug Resistance of Staphylococcus Aureus in Feces From Pediatric Patients in Guangzhou, China. *Front. Med.* **7**, 1–10 (2020).
5. Horváth, A. *et al.* Characterisation of antibiotic resistance, virulence, clonality and mortality in MRSA and MSSA bloodstream infections at a tertiary-level hospital in Hungary: A 6-year retrospective study. *Ann. Clin. Microbiol. Antimicrob.* **19**, 1–11 (2020).
6. Bastidas, C. A. *et al.* Antibiotic susceptibility profile and prevalence of mecA and lukS-PV/lukF-PV genes in Staphylococcus aureus isolated from nasal and pharyngeal sources of medical students in Ecuador. *Infect. Drug Resist.* **12**, 2553–2560 (2019).
7. Vahedian-Ardakani, H. A., Moghimi, M., Shayestehpour, M., Doosti, M. & Amid, N. Bacterial spectrum and antimicrobial resistance pattern in cancer patients with febrile neutropenia. *Asian Pacific J. Cancer Prev.* **20**, 1471–1474 (2019).
8. Chabi, R. & Momtaz, H. Virulence factors and antibiotic resistance properties of the Staphylococcus epidermidis strains isolated from hospital infections in Ahvaz, Iran. *Trop. Med. Health* **47**, 1–9 (2019).
9. Hays, C. *et al.* Changing epidemiology of group B Streptococcus susceptibility to fluoroquinolones and aminoglycosides in France. *Antimicrob. Agents Chemother.* **60**, 7424–7430 (2016).
10. Beheshti, M. *et al.* Molecular characterization, antibiotic resistance pattern and capsular types of invasive Streptococcus pneumoniae isolated from clinical samples in Tehran, Iran. *BMC Microbiol.* **20**, 1–9 (2020).
11. D, D. *et al.* In vitro activity of omadacycline against pathogens isolated from Mainland China during 2017-2018. *Eur. J. Clin. Microbiol. Infect. Dis.* **39**, 1559–1572 (2020).
12. Wada, F. W., Tufa, E. G., Berheto, T. M. & Solomon, F. B. Nasopharyngeal carriage of Streptococcus pneumoniae and antimicrobial susceptibility pattern among school children in South Ethiopia: Post-vaccination era. *BMC Res. Notes* **12**, 1–6 (2019).
13. Mayanskiy, N. *et al.* Changing serotype distribution and resistance patterns among pediatric nasopharyngeal pneumococci collected in Moscow, 2010–2017. *Diagn. Microbiol. Infect. Dis.* **94**, 385–390 (2019).
14. Rothe, K. *et al.* Antimicrobial resistance of bacteraemia in the emergency department of a German university hospital (2013-2018): Potential carbapenem-sparing empiric treatment

options in light of the new EUCAST recommendations. *BMC Infect. Dis.* **19**, 1–10 (2019).

1. Lee, M. C. *et al.* The antimicrobial susceptibility in adult invasive pneumococcal disease in the era of pneumococcus vaccination: A hospital-based observational study in Taiwan. *J. Microbiol. Immunol. Infect.* **53**, 836–844 (2020).
2. Micek, S. T., Simmons, J., Hampton, N. & Kollef, M. H. Characteristics and outcomes among a hospitalized patient cohort with Streptococcus pneumoniae infection. *Medicine (Baltimore).* **99**, e20145 (2020).
3. Nakano, S. *et al.* Nationwide surveillance of paediatric invasive and non-invasive pneumococcal disease in Japan after the introduction of the 13-valent conjugated vaccine, 2015–2017. *Vaccine* **38**, 1818–1824 (2020).
4. Park, D. C. *et al.* Serotype distribution and antimicrobial resistance of invasive and noninvasive streptococcus pneumoniae isolates in korea between 2014 and 2016. *Ann. Lab. Med.* **39**, 537– 544 (2019).
5. John, J., Varghese, R., Lionell, J., Neeravi, A. & Veeraraghavan, B. Non-vaccine Pneumococcal Serotypes Among Children with Invasive Pneumococcal Disease. *Indian Pediatr.* **55**, 874–876 (2018).
6. Torumkuney, D. *et al.* Results from the survey of antibiotic resistance (SOAR) 2015-18 in Tunisia, Kenya and Morocco: Data based on CLSI, EUCAST (dose-specific) and pharmacokinetic/pharmacodynamic (PK/PD) breakpoints. *J. Antimicrob. Chemother.* **75**, (2020).
7. LY, G. *et al.* Clinical and pathogenic analysis of 507 children with bacterial meningitis in Beijing, 2010-2014. *Int. J. Infect. Dis.* **50**, 38–43 (2016).
8. Ke, R. *et al.* Bacteriological profiles and drug susceptibility of Streptococcus isolated from conjunctival sac of healthy children. *BMC Pediatr.* **20**, 1–5 (2020).
9. Li, X. X. *et al.* Serotype Distribution, Antimicrobial Susceptibility, and Multilocus Sequencing Type (MLST) of Streptococcus pneumoniae From Adults of Three Hospitals in Shanghai, China. *Front. Cell. Infect. Microbiol.* **9**, 1–11 (2019).
10. Kengne, M., Lebogo, M. B. B., Nwobegahay, J. M. & Ondigui, B. E. Antibiotics susceptibility pattern of Streptococcus pneumoniae isolated from sputum cultures of human immunodeficiency virus infected patients in Yaoundé, Cameroon. *Pan Afr. Med. J.* **31**, 1–6 (2018).
11. Hua, C. Z. *et al.* A multi-center clinical investigation on invasive Streptococcus pyogenes infection in China, 2010-2017. *BMC Pediatr.* **19**, 1–6 (2019).
12. Berwal, A., Chawla, K., Shetty, S. & Gupta, A. Trend of antibiotic Susceptibility of streptococcus pyogenes isolated from respiratory tract infections in tertiary care hospital in South Karnataka. *Iran. J. Microbiol.* **11**, 13–18 (2019).
13. Anja, A., Beyene, G., S/Mariam, Z. & Daka, D. Asymptomatic pharyngeal carriage rate of Streptococcus pyogenes, its associated factors and antibiotic susceptibility pattern among school children in Hawassa town, southern Ethiopia. *BMC Res. Notes* **12**, 564 (2019).
14. Jamsheer, A., Rafay, A. M., Daoud, Z., Morrissey, I. & Torumkuney, D. Results from the Survey of Antibiotic Resistance (SOAR) 2011-13 in the Gulf States. *J. Antimicrob. Chemother.* **71**, i45–i61

(2016).

1. Torumkuney, D. *et al.* Results from the Survey of Antibiotic Resistance (SOAR) 2012-14 in Thailand, India, South Korea and Singapore. *J. Antimicrob. Chemother.* **71**, i3–i19 (2016).
2. Lu, B. *et al.* High prevalence of macrolide-resistance and molecular characterization of Streptococcus pyogenes isolates circulating in China from 2009 to 2016. *Front. Microbiol.* **8**, 1–10 (2017).
3. Jean, S. S., Lee, W. Sen, Ko, W. C. & Hsueh, P. R. In vitro susceptibility of ceftaroline against clinically important Gram-positive cocci, Haemophilus species and Klebsiella pneumoniae in Taiwan: Results from the Antimicrobial Testing Leadership and Surveillance (ATLAS) in 2012– 2018. *J. Microbiol. Immunol. Infect.* **54**, 627–631 (2021).
4. Oppegaard, O., Skrede, S., Mylvaganam, H. & Kittang, B. R. Emerging Threat of Antimicrobial

Resistance in β-Hemolytic Streptococci. *Front. Microbiol.* **11**, 1–12 (2020).

1. Luiz, F. B. de O., Alves, K. B. & Barros, R. R. Prevalence and long-term persistence of beta- haemolytic streptococci throat carriage among children and young adults. *J. Med. Microbiol.* **68**, 1526–1533 (2019).
2. De Muri, G. P. *et al.* Macrolide and clindamycin resistance in group a streptococci isolated from children with pharyngitis. *Pediatr. Infect. Dis. J.* **36**, 342–344 (2017).
3. Sánchez-Encinales, V. *et al.* Molecular Characterization of Streptococcus pyogenes Causing Invasive Disease in Pediatric Population in Spain A 12-year Study. *Pediatr. Infect. Dis. J.* **38**, 1168– 1172 (2020).
4. de Paula, A., Oliva, G., Barraquer, R. I. & de la Paz, M. F. Prevalence and antibiotic susceptibility of bacteria isolated in patients affected with blepharitis in a tertiary eye centre in Spain. *Eur. J. Ophthalmol.* **30**, 991–997 (2020).
5. Guerrero-Del-Cueto, F., Ibanes-Gutiérrez, C., Velázquez-Acosta, C., Cornejo-Juárez, P. & Vilar- Compte, D. Microbiology and clinical characteristics of viridans group streptococci in patients with cancer. *Brazilian J. Infect. Dis.* **22**, 323–327 (2018).
6. Kim, Y. H. & Lee, S. Y. Antibiotic resistance of viridans group streptococci isolated from dental plaques. *Biocontrol Sci.* **25**, 173–178 (2020).
7. P, L., B, S.-L., A, M., A, S.-G. & G, M. An Analysis of Resistance Patterns of Oral Streptococci Obtained from Orofacial Infections Against Beta-lactams, Clindamycin and Vancomycin over 2014-2018. *Oral Health Prev. Dent.* **17**, 585–589 (2019).
8. Loyola-Rodriguez, J. P. *et al.* Determination and identification of antibiotic-resistant oral streptococci isolated from active dental infections in adults. *Acta Odontol. Scand.* **76**, 229–235 (2018).
9. Li, Y. *et al.* Gene subtype analysis of treponema pallidum for drug resistance to azithromycin. *Exp. Ther. Med.* **16**, 1009–1013 (2018).
10. Shuel, M., Hayden, K., Kadkhoda, K. & Tsang, R. S. W. Molecular typing and macrolide resistance of syphilis cases in Manitoba, Canada, from 2012 to 2016. *Sex. Transm. Dis.* **45**, 233–236 (2018).
11. Zhu, X., Li, M., Cao, H., Yang, X. & Zhang, C. Epidemiology of Ureaplasma urealyticum and

Mycoplasma hominis in the semen of male outpatients with reproductive disorders. *Exp. Ther. Med.* **12**, 1165–1170 (2016).

1. Gu, X., Liu, S., Guo, X., Weng, R. & Zhong, Z. Epidemiological investigation and antimicrobial susceptibility analysis of mycoplasma in patients with genital manifestations. *J. Clin. Lab. Anal.* **34**, 1–6 (2020).
2. D, S., D, M. & J, V. Prevalence and antibiotic susceptibility of Mycoplasma hominis and Ureaplasma urealyticum in genital samples collected over 6 years at a Serbian university hospital. *Indian J. Dermatol. Venereol. Leprol.* **82**, 37–41 (2016).
3. MA, V.-K. & MB, B. Antibacterial Resistance in Ureaplasma Species and Mycoplasma hominis Isolates from Urine Cultures in College-Aged Females. *Antimicrob. Agents Chemother.* **61**, (2017).
4. Zeng, X. Y., Xin, N., Tong, X. N., Wang, J. Y. & Liu, Z. W. Prevalence and antibiotic susceptibility of Ureaplasma urealyticum and Mycoplasma hominis in Xi’an, China. *Eur. J. Clin. Microbiol. Infect. Dis.* **35**, 1941–1947 (2016).
5. Lee, M., Kim, M., Lee, W., Kang, S. & Jeon, Y. Prevalence and antibiotic susceptibility of Mycoplasma hominis and Ureaplasma urealyticum in pregnant women. *Yonsei Med. J.* **57**, 1271– 1275 (2016).
6. Liu, Y. *et al.* Chlamydia trachomatis and mycoplasma infections in tubal pregnancy. *Sci. Rep.* **9**, 5– 9 (2019).
7. Zhang, W. *et al.* Infection Prevalence and Antibiotic Resistance Levels in Ureaplasma urealyticum and Mycoplasma hominis in Gynecological Outpatients of a Tertiary Hospital in China from 2015 to 2018. *Can. J. Infect. Dis. Med. Microbiol.* **2021**, (2021).
8. He, M. *et al.* Prevalence and antimicrobial resistance of Mycoplasmas and Chlamydiae in patients with genital tract infections in Shanghai, China. *J. Infect. Chemother.* **22**, 548–552 (2016).
9. Maldonado-Arriaga, B. *et al.* Mollicutes antibiotic resistance profile and presence of genital abnormalities in couples attending an infertility clinic. *J. Int. Med. Res.* **48**, (2020).
10. Fernández, J., Karau, M. J., Cunningham, S. A., Greenwood-Quaintance, K. E. & Patel, R. Antimicrobial susceptibility and clonality of clinical Ureaplasma isolates in the United States. *Antimicrob. Agents Chemother.* **60**, 4793–4798 (2016).
11. Dua, P., Karmakar, A. & Ghosh, C. Virulence gene profiles, biofilm formation, and antimicrobial resistance of Vibrio cholerae non-O1/non-O139 bacteria isolated from West Bengal, India. *Heliyon* **4**, e01040 (2018).
12. Dengo-Baloi, L. C. *et al.* Antibiotics resistance in El Tor Vibrio cholerae 01 isolated during cholera outbreaks in Mozambique from 2012 to 2015. *PLoS One* **12**, 1–10 (2017).
13. N, R., B, B. & S, N.-P. Distribution of resistance genetic determinants among Vibrio cholerae isolates of 2012 and 2013 outbreaks in IR Iran. *Microb. Pathog.* **104**, 12–16 (2017).
14. Yousefi, A., Vaez, H., Sahebkar, A. & Khademi, F. A systematic review and meta-analysis on the epidemiology of antibiotic resistance of Vibrio cholerae in Iran. *Ann. di Ig. Med. Prev. e di Comunita* **31**, 279–290 (2019).

# Supplemental Table S5: Pathogen factors and citations

| Pathogen | Pathogen type | Reservoir | Trans-mission Mode* | **Noso-comial (Yes-1 or No-0)** | **Zoo-notic**  **(Yes-1 or** No-0) | **Human to Human trans-mission** (Yes-1 or No-0) | **Com-mensals** (Yes-1 or No-0) | **Conju-gation** (Yes-1 or No-0) | **Naturally Competent** (Yes-1 or No-0) | Citations |
| --- | --- | --- | --- | --- | --- | --- | --- | --- | --- | --- |
| *Acinetobacter spp* | Gram positive | Environment | Vehicle-Borne | 1 | No | 1 | Yes | 1 | Yes | 1–4 |
| *Actinomyces spp* | Other | Human | Direct Contact | 0 | No | 0 | Yes | 0 | No | 4–6 |
| *Bacillus anthracis* | Gram positive | Animal | Direct Contact | 0 | Yes | 0 | No | 1 | No | 4,7–10 |
| *Bacteroides spp* | Anaerobic | Human | Vehicle-Borne | 1 | No | 0 | Yes | 1 | No | 4,11–14 |
| *Bordetella pertussis* | Gram negative | Human | Droplet | 1 | No | 1 | No | 1 | No | 4,15–17 |
| *Borrelia burgdorferi* | Other | Animal | Vector-Borne | 0 | Yes | 0 | No | 0 | No | 4,18,19 |
| *Brucella spp* | Gram negative | Animal | Vehicle-Borne | 0 | Yes | 0 | No | 1 | No | 4,20–22 |
| *Campylobacter jejuni* | Gram negative | Animal | Vehicle-Borne | 0 | Yes | 0 | No | 1 | Yes | 4,23–26 |
| *Chlamydia pneumoniae* | Other | Human | Droplet | 1 | No | 1 | No | 0 | No | 4,27–29 |
| *Chlamydia psittaci* | Other | Animal | Airborne | 0 | Yes | 0 | No | 0 | No | 4,30–32 |
| *Chlamydia trachomatis* | Other | Human | Direct Contact | 0 | No | 1 | No | 0 | No | 4,33,34 |
| *Citrobacter spp* | Gram negative | Human | Vehicle-Borne | 1 | No | 0 | Yes | 1 | No | 4,35–38 |
| *Clostridium difficile* | Anaerobic | Environment | Vehicle-Borne | 1 | No | 1 | Yes | 1 | No | 4,39–41 |
| *Clostridium perfringens* | Anaerobic | Environment | Vehicle-Borne | 0 | No | 0 | Yes | 1 | No | 4,42,43 |
| *Clostridium spp* | Anaerobic | Environment | Direct Contact (excluding C. botulinum) | 0 | No | 0 | Yes | 1 | No | 4,44–46 |
| *Clostridium tetani* | Anaerobic | Environment | Direct Contact | 0 | No | 0 | No | 0 | No | 4,47 |
| *Corynebacterium diptheriae* | Gram positive | Human | Droplet | 0 | No | 1 | No | 1 | No | 4,48,49 |
| *Enterobacter aerogenes* | Gram negative | Human | Vehicle-Borne | 1 | No | 1 | Yes | 1 | No | 4,50–53 |
| *Enterococcus faecalis* | Gram positive | Human | Vehicle-Borne | 1 | Yes | 1 | Yes | 1 | No | 4,54–57 |
| *Escherichia coli (ETEC)* | Gram negative | Animal | Vehicle-Borne | 1 | Yes | 1 | Yes | 1 | Yes | 4,58–60 |
| *Francisella tularensis* | Gram negative | Animal | Vehicle-Borne | 0 | Yes | 0 | No | 0 | No | 4,61–63 |
| *Fuseobacterium spp* | Anaerobic | Human | Direct Contact | 1 | No | 0 | Yes | 1 | No | 4,64–67 |
| *Gardnerella vaginalis* | Gram negative | Human | Direct Contact | 0 | No | 1 | Yes | 0 | No | 4,68–73 |
| *GPAC* | Anaerobic | Human | Vehicle-Borne (Fecal-Oral) | 0 | No | 1 | Yes | 0 | No | 4,74 |
| *Haemophilus influenzae* | Gram negative | Human | Droplet | 1 | No | 1 | Yes | 1 | Yes | 4,75–78 |
| *Klebsiella oxytoca* | Gram negative | Human | Direct Contact | 1 | No | 1 | Yes | 1 | No | 4,64,79–81 |
| *Klebsiella pneumoniae* | Gram negative | Human | Direct Contact | 1 | No | 1 | Yes | 1 | No | 4,59,82,83 |
| *Klebsiella spp* | Gram negative | Human | Direct Contact | 1 | No | 1 | Yes | 1 | No | 4,80,84,85 |
| *Legionella pneumophila* | Gram negative | Environment | Airborne | 1 | No | 0 | No | 1 | Yes | 4,30,86,87 |
| *Leptospira interrogans* | Other | Animal | Direct Contact | 0 | Yes | 0 | No | 1 | No | 4,88–90 |
| *Listeria monocytogenes* | Gram positive | Environment | Vehicle-Borne | 0 | No | 1 | No | 1 | No | 4,91–93 |
| *Moraxella catarrhalis* | Gram negative | Human | Direct Contact | 1 | No | 0 | Yes | 1 | Yes | 4,94–97 |
| *Mycoplasma pneumoniae* | Other | Human | Droplet | 0 | No | 1 | No | 0 | No | 4,98,99 |
| *Neisseria gonorrhoeae* | Gram negative | Human | Direct Contact | 0 | No | 1 | No | 1 | Yes | 4,100–103 |
| *Neisseria meningitidis* | Gram negative | Human | Direct | 0 | No | 1 | Yes | 1 | Yes | 4,104–106 |
| *Nocardia spp* | Other | Environment | Airborne | 0 | No | 0 | No | 1 | No | 4,107–109 |
| *Non-typhoidal Salmonella* | Gram negative | Animal | Vehicle-Borne | 0 | Yes | 1 | No | 1 | No | 4,110–112 |
| *Propiniobacterium acnes* | Anaerobic | Human | Vehicle-Borne | 1 | No | 0 | Yes | 1 | No | 4,113–115 |
| *Proteus mirabilis* | Gram negative | Human | Vehicle-Borne | 1 | No | 0 | Yes | 1 | No | 4,116–118 |
| *Proteus spp* | Gram negative | Human | Vehicle-Borne | 1 | No | 0 | Yes | 1 | No | 4,119–121 |
| *Providencia spp* | Gram negative | Human | Vehicle-Borne | 1 | No | 0 | Yes | 1 | No | 4,122–125 |
| *Pseudomonas aeruginosa* | Gram negative | Environment | Vehicle-Borne | 1 | No | 1 | Yes | 1 | Yes | 4,126–128 |
| *Rickettsia rickettsii* | Other | Animal | Vector-Borne | 0 | Yes | 0 | No | 0 | No | 4,129,130 |
| *Salmonella typhi* | Gram negative | Human | Vehicle-Borne | 0 | No | 1 | No | 1 | No | 4,93,131,132 |
| *Serratia marcescens* | Gram negative | Environment | Direct Contact | 1 | No | 0 | No | 1 | No | 4,133–136 |
| *Shigella species* | Gram negative | Human | Vehicle-Borne | 0 | No | 1 | No | 1 | No | 4,137,138 |
| *Staphylococcus aureus* | Gram positive | Human | Direct Contact | 1 | No | 1 | Yes | 1 | Yes | 4,139–141 |
| *Staphylococcus epidermidis* | Gram positive | Human | Vehicle-Borne | 1 | No | 0 | Yes | 1 | No | 4,142,143 |
| *Streptococcus agalactiae* | Gram positive | Human | Direct Contact | 1 | No | 0 | Yes | 1 | No | 4,144–146 |
| *Streptococcus pneumoniae* | Gram positive | Human | Droplet | 1 | No | 1 | Yes | 1 | Yes | 4,147,148 |
| *Streptococcus pyogenes* | Gram positive | Human | Droplet | 1 | No | 1 | Yes | 1 | No | 4,149,150 |
| *Streptococcus viridans* | Gram positive | Human | Direct | 1 | No | 0 | Yes | 1 | No | 4,151–153 |
| *Treponema pallidum* | Other | Human | Direct Contact | 0 | No | 1 | No | 0 | No | 4,154 |
| *Treponema pallidum pertenue* | Other | Human | Direct Contact | 0 | No | 1 | No | 0 | No | 4,155 |
| *Ureaplasma urealyticum* | Other | Human | Direct Contact | 0 | No | 1 | Yes | 1 | No | 4,156,157 |
| *Vibrio cholerae* | Gram negative | Environment | Vehicle-Borne | 0 | No | 1 | No | 1 | Yes | 4,158–160 |
| *Yersinia pestis* | Gram negative | Animal | Vector-Borne | 0 | Yes | 0 | No | 1 | No | 4,135,161 |

*Transmission modes were analysed as Direct or Indirect. Direct modes of transmission include ‘Direct Contact’ and 'Droplet' whereas Indirect transmission includes ‘Vector-Borne’, ‘Air-borne’, and 'Vehicle-borne'. Note that water is considered a vehicle.

1. Atrouni, A. Al, Joly-Guillou, M. L., Hamze, M. & Kempf, M. Reservoirs of non-baumannii Acinetobacter species. *Front. Microbiol.* **7**, 1–12 (2016).
2. Leungtongkam, U., Thummeepak, R., Tasanapak, K. & Sitthisak, S. Acquisition and transfer of antibiotic resistance genes in association with conjugative plasmid or class 1 integrons of Acinetobacter baumannii. *PLoS One* **13**, 1–12 (2018).
3. Venanzio, G. Di *et al.* Multidrug-resistant plasmids repress chromosomally encoded T6SS to enable their dissemination. *Proc. Natl. Acad. Sci. U. S. A.* **116**, 1378–1383 (2019).
4. Johnsborg, O., Eldholm, V. & Håvarstein, L. S. Natural genetic transformation: prevalence, mechanisms and function. *Res. Microbiol.* **158**, 767–778 (2007).
5. Könönen, E. & Wade, W. G. Actinomyces and related organisms in human infections. *Clin. Microbiol. Rev.* **28**, 419–442 (2015).
6. Bowden, G. H. W. *Actinomyces, Propionibacterium propionicus, and Streptomyces*. *Medical Microbiology* (University of Texas Medical Branch at Galveston, 1996).
7. What is Anthrax? | CDC. *Centers for Disease Control and Prevention, National Center for Emerging and Zoonotic Infectious Diseases (NCEZID)* [https://w](http://www.cdc.gov/anthrax/basics/index.html)ww.cdc.g[ov/anthrax/basics/index.html](http://www.cdc.gov/anthrax/basics/index.html) (2020).
8. Koehler, T. M. Bacillus anthracis Physiology and Genetics. *Mol. Aspects Med.* **30**, 386–396 (2009).
9. Saile, E. & Koehler, T. M. Bacillus anthracis multiplication, persistence, and genetic exchange in the rhizosphere of grass plants. *Appl. Environ. Microbiol.* **72**, 3168–3174 (2006).
10. Yuan, Y., Zheng, D., Hu, X., Cai, Q. & Yuan, Z. Conjugative Transfer of Insecticidal Plasmid pHT73 from Bacillus thuringiensis to B. anthracis and Compatibility of This Plasmid with pXO1 and pXO2. *Appl. Environ. Microbiol.* **76**, 468–473 (2010).
11. Patrick, S. Bacteroides. in *Molecular Medical microbiology* 917–944 (Elsevier, 2014).
12. Shoemaker, N. B., Vlamakis, H., Hayes, K. & Salyers, A. A. Evidence for extensive resistance gene transfer among Bacteroides spp. and among Bacteroides and other genera in the human colon. *Appl. Environ. Microbiol.* **67**, 561–568 (2001).
13. Nguyen, M. & Vedantam, G. Mobile genetic elements in the genus Bacteroides, and their mechanism(s) of dissemination. *Mob. Genet. Elements* **1**, 187–196 (2011).
14. Pathogen Safety Data Sheets: Infectious Substances – Bacteroides spp. - Canada.ca. [https://w](http://www.canada.ca/en/public-)ww.[canada.](http://www.canada.ca/en/public-)ca/e[n/publ](http://www.canada.ca/en/public-)i[c-](http://www.canada.ca/en/public-) health/services/laboratory-biosafety-biosecurity/pathogen-safety-data-sheets-risk-assessment/bacteroides.html.
15. Weiss, A. A. & Falkow, S. Genetic analysis of phase change in Bordetella pertussis. *Infect. Immun.* **43**, 263–269 (1984).
16. Weiss, A. A. & Falkow, S. Plasmid transfer to Bordetella pertussis: Conjugation and transformation. *J. Bacteriol.* **152**, 549–552 (1982).
17. OCHMAN, H. Evolution of Bacterial Pathogens. *Princ. Bact. Pathog.* 1–41 (2001) doi:10.1016/B978-012304220-0/50002-9.
18. Transmission | Lyme Disease | CDC. [https://w](http://www.cdc.gov/lyme/transmission/index.html)ww.cdc.g[ov/lyme/transmission/index.html.](http://www.cdc.gov/lyme/transmission/index.html)
19. Brisson, D., Drecktrah, D., Eggers, C. H. & Samuels, D. S. Genetics of Borrelia burgdorferi. *Annu. Rev. Genet.* **46**, 515–536 (2012).
20. Verger, J. M., Grayon, M., Chaslus-Dancla, E., Meurisse, M. & Lafont, J. P. Conjugative Transfer and in Vitro/in Vivo Stability of the Broad- Host-Range IncP R751 Plasmid in Brucella spp. *Plasmid* **29**, 142–146 (1992).
21. Wattam, A. R. *et al.* Analysis of ten Brucella genomes reveals evidence for horizontal gene transfer despite a preferred intracellular lifestyle. *J. Bacteriol.* **191**, 3569–3579 (2009).
22. Transmission | Brucellosis | CDC. [https://w](http://www.cdc.gov/brucellosis/transmission/index.html)ww.cdc.g[ov/brucellosis/transmission/index.html.](http://www.cdc.gov/brucellosis/transmission/index.html)
23. Questions and Answers | Campylobacter | CDC. [https://w](http://www.cdc.gov/campylobacter/faq.html)ww.cdc.g[ov/campylobacter/faq.html.](http://www.cdc.gov/campylobacter/faq.html)
24. Wilson, D. J. *et al.* Tracing the source of campylobacteriosis. *PLoS Genet.* **4**, (2008).
25. Zeng, X., Wu, Z., Zhang, Q. & Lin, J. A cotransformation method to identify a restriction-modification enzyme that reduces conjugation efficiency in Campylobacter jejuni. *Appl. Environ. Microbiol.* **84**, 1–13 (2018).
26. Avrain, L., Vernozy-Rozand, C. & Kempf, I. Evidence for natural horizontal transfer of tetO gene between Campylobacter jejuni strains in chickens. *J. Appl. Microbiol.* **97**, 134–140 (2004).
27. Chlamydia pneumoniae: Causes, How It Spreads, and Risk Factors | CDC. [https://w](http://www.cdc.gov/pneumonia/atypical/cpneumoniae/about/causes.html)ww.cdc.g[ov/pneumonia/atypical/cpneumoniae/about/causes.html.](http://www.cdc.gov/pneumonia/atypical/cpneumoniae/about/causes.html)
28. Sixt, B. S. & Valdivia, R. H. Molecular Genetic Analysis of Chlamydia Species. *Annu. Rev. Microbiol.* **70**, 179–198 (2016).
29. M, R. & KA, F. Transformation of Chlamydia: current approaches and impact on our understanding of chlamydial infection biology.

*Microbes Infect.* **20**, 445–450 (2018).

1. González-Rivera, E. M. *et al.* Antibiotic resistance, virulence factors and genotyping of pseudomonas aeruginosa in public hospitals of northeastern mexico. *J. Infect. Dev. Ctries.* **13**, 374–383 (2019).
2. Hooppaw, A. J. & Fisher, D. J. A coming of age story: Chlamydia in the post-genetic era. *Infect. Immun.* **84**, 612–621 (2016).
3. Psittacosis: Clinical Disease Specifics | CDC. [https://w](http://www.cdc.gov/pneumonia/atypical/psittacosis/hcp/disease-specifics.html)ww.cdc.g[ov/pneumonia/atypical/psittacosis/hcp/disease-specifics.html.](http://www.cdc.gov/pneumonia/atypical/psittacosis/hcp/disease-specifics.html)
4. Detailed STD Facts - Chlamydia. https://[www.cdc.gov/std/chlamydia/stdfact-chlamydia-detailed.htm.](http://www.cdc.gov/std/chlamydia/stdfact-chlamydia-detailed.htm)
5. DeMars, R., Weinfurter, J., Guex, E., Lin, J. & Potucek, Y. Lateral gene transfer in vitro in the intracellular pathogen Chlamydia trachomatis.

*J. Bacteriol.* **189**, 991–1003 (2007).

1. Yuan, C. *et al.* Comparative Genomic Analysis of Citrobacter and Key Genes Essential for the Pathogenicity of Citrobacter koseri. *Front. Microbiol.* **10**, 1–15 (2019).
2. Doran, T. I. The role of Citrobacter in clinical disease of children: Review. *Clin. Infect. Dis.* **28**, 384–394 (1999).
3. Nayar, Ritu., Shukla, A. I. Epidemiology, Prevalence and identification of Citobacter Spesies in Clinical Spesimen in a Tertiary Care Hospital in India. *Int. J. Sci. Res. Publ.* **4**, 2250–3153 (2014).
4. Virolle, C., Goldlust, K., Djermoun, S., Bigot, S. & Lesterlin, C. Plasmid transfer by conjugation in gram-negative bacteria: From the cellular to the community level. *Genes (Basel).* **11**, 1–33 (2020).
5. Brouwer, M. S. M. *et al.* Horizontal gene transfer converts non-toxigenic Clostridium difficile strains into toxin producers. *Nat. Commun.*

**4**, 1–6 (2013).

1. Sebaihia, M. *et al.* The multidrug-resistant human pathogen Clostridium difficile has a highly mobile, mosaic genome. *Nat. Genet.* **38**, 779–786 (2006).
2. What is C. diff? | CDC. https://[www.cdc.gov/cdiff/what-is.html#factsheet.](http://www.cdc.gov/cdiff/what-is.html#factsheet)
3. C. perfringens | CDC. [https://w](http://www.cdc.gov/foodsafety/diseases/clostridium-perfringens.html)ww.cdc.g[ov/foodsafety/diseases/clostridium-perfringens.html.](http://www.cdc.gov/foodsafety/diseases/clostridium-perfringens.html)
4. Wisniewski, J. A. & Rood, J. I. The Tcp conjugation system of Clostridium perfringens. *Plasmid* **91**, 28–36 (2017).
5. Pathogen Safety Data Sheets: Infectious Substances – Clostridium spp. - Canada.ca. https://[www.canada.ca/en/public-](http://www.canada.ca/en/public-) health/services/laboratory-biosafety-biosecurity/pathogen-safety-data-sheets-risk-assessment/clostridium.html.
6. Philipps, G., De Vries, S. & Jennewein, S. Development of a metabolic pathway transfer and genomic integration system for the syngas- fermenting bacterium Clostridium ljungdahlii. *Biotechnol. Biofuels* **12**, 1–14 (2019).
7. Vidor, C. J. *et al.* Clostridium sordellii Pathogenicity Locus Plasmid pCS1-1 Encodes a Novel Clostridial Conjugation Locus. *Am. Soc. Microbiol.* **9**, 1–14 (2018).
8. Tetanus Causes and Transmission | CDC. [https://w](http://www.cdc.gov/tetanus/about/causes-transmission.html)ww.cdc.g[ov/tetanus/about/causes-transmission.html.](http://www.cdc.gov/tetanus/about/causes-transmission.html)
9. Diphtheria: Causes and Spread to Others | CDC. https://[www.cdc.gov/diphtheria/about/causes-transmission.html.](http://www.cdc.gov/diphtheria/about/causes-transmission.html)
10. Hennart, M. *et al.* Population genomics and antimicrobial resistance in Corynebacterium diphtheriae. *Genome Med.* **12**, 1–18 (2020).
11. Pathogen Safety Data Sheets: Infectious Substances – Enterobacter spp. - Canada.ca. https://[www.canada.ca/en/public-](http://www.canada.ca/en/public-) health/services/laboratory-biosafety-biosecurity/pathogen-safety-data-sheets-risk-assessment/enterobacter.html.
12. Davin-Regli, A. *et al.* Molecular epidemiology of Enterobacter aerogenes acquisition: One-year prospective study in two intensive care units. *J. Clin. Microbiol.* **34**, 1474–1480 (1996).
13. Burmølle, M., Bahl, M. I., Jensen, L. B., Sørensen, S. J. & Hansen, L. H. Type 3 fimbriae, encoded by the conjugative plasmid pOLA52, enhance biofilm formation and transfer frequencies in Enterobacteriaceae strains. *Microbiology* **154**, 187–195 (2008).
14. Chavda, K. D. *et al.* Comprehensive genome analysis of carbapenemase-producing Enterobacter spp.: New insights into phylogeny, population structure, and resistance mechanisms. *MBio* **7**, 1–16 (2016).
15. Audrey Wanger, V. C. et al. Chapter 6. Overview of Bacteria | Elsevier Enhanced Reader. in *Microbiology and Molecular Diagnosis in Pathology* 97–98 (2017).
16. Pathogen Safety Data Sheets: Infectious Substances – Enterococcus faecalis and Enterococcus faecium - Canada.ca. [https://w](http://www.canada.ca/en/public-health/services/laboratory-biosafety-biosecurity/pathogen-safety-data-sheets-risk-)ww.[canada.](http://www.canada.ca/en/public-health/services/laboratory-biosafety-biosecurity/pathogen-safety-data-sheets-risk-)ca/e[n/publ](http://www.canada.ca/en/public-health/services/laboratory-biosafety-biosecurity/pathogen-safety-data-sheets-risk-)i[c-health/services/laboratory-biosafety-biosecurity/pathogen-safety-data-sheets-risk-](http://www.canada.ca/en/public-health/services/laboratory-biosafety-biosecurity/pathogen-safety-data-sheets-risk-) assessment/enterococcus-faecalis.html.
17. Frost, S. Bacterial conjugation : everybody ’ s doin ’ it. *Can. J. Microbiol.* 1091–1096 (1992).
18. Hirt, H. *et al.* Enterococcus faecalis sex pheromone cCF10 enhances conjugative plasmid transfer in vivo. *MBio* **9**, (2018).
19. Enterotoxigenic E. coli (ETEC) | E. coli | CDC. [https://w](http://www.cdc.gov/ecoli/etec.html)ww.cdc.g[ov/ecoli/etec.html.](http://www.cdc.gov/ecoli/etec.html)
20. Lerminiaux, N. A. & Cameron, A. D. S. Horizontal transfer of antibiotic resistance genes in clinical environments. *Can. J. Microbiol.* **65**, 34– 44 (2019).
21. Murray, B. E., Evans, D. J., Penaranda, M. E. & Evans, D. G. CFA/I-ST plasmids: Comparison of enterotoxigenic Escherichia coli (ETEC) of serogroups O25, O63, O78, and O128 and mobilization from an R factor-containing epidemic ETEC isolate. *J. Bacteriol.* **153**, 566–570 (1983).
22. Transmission | Tularemia | CDC. [https://w](http://www.cdc.gov/tularemia/transmission/index.html)ww.cdc.g[ov/tularemia/transmission/index.html.](http://www.cdc.gov/tularemia/transmission/index.html)
23. World Health Organization. *WHO guidelines on tularaemia: epidemic and pandemic alert and response*. <http://www.who.int/csr/resources/publications/WHO_CDS_EPR_2007_7.pdf> (2007).
24. Siddaramappa, S., Challacombe, J. F., Petersen, J. M., Pillai, S. & Kuske, C. R. Comparative analyses of a putative Francisella conjugative

element. *Genome* **57**, 137–144 (2014).

1. Garrett, W. S. & Onderdonk, A. B. Bacteroides, Prevotella, Porphyromonas, and Fusobacterium Species (and Other Medically Important Anaerobic Gram-Negative Bacilli). in *Mandell, Douglas, and Bennett’s Principles and Practice of Infectious Diseases* vol. 2 2773–2780 (2014).
2. Roberts, M. C. & Lansciardi, J. Transferable Tet M in Fusobacterium nucleatum. *Antimicrob. Agents Chemother.* **34**, 1836–1838 (1990).
3. Claypool, B. M. *et al.* Mobilization and prevalence of a fusobacterial plasmid. *Plasmid* **63**, 11–19 (2010).
4. Riordan, T. Human infection with Fusobacterium necrophorum (Necrobacillosis), with a focus on Lemierre’s syndrome. *Clin. Microbiol. Rev.* **20**, 622–659 (2007).
5. STD Facts - Bacterial Vaginosis. [https://w](http://www.cdc.gov/std/bv/stdfact-bacterial-vaginosis.htm)ww.cdc.g[ov/std/bv/stdfact-bacterial-vaginosis.htm.](http://www.cdc.gov/std/bv/stdfact-bacterial-vaginosis.htm)
6. Harwich, M. D. *et al.* Drawing the line between commensal and pathogenic Gardnerella vaginalis through genome analysis and virulence studies. *BMC Genomics* **11**, (2010).
7. Schwebke, J. R., Muzny, C. A. & Josey, W. E. Role of Gardnerella vaginalis in the pathogenesis of bacterial vaginosis: A conceptual model.

*J. Infect. Dis.* **210**, 338–343 (2014).

1. Catlin, B. W. Gardnerella vaginalis: Characteristics, clinical considerations, and controversies. *Clin. Microbiol. Rev.* **5**, 213–237 (1992).
2. Huang, R. *et al.* Molecular evolution of the tet(M) gene in Gardnerella vaginalis. *J. Antimicrob. Chemother.* **40**, 561–565 (1997).
3. Roberts, M. C. Characterization of the Tet M determinants in urogenital and respiratory bacteria. *Antimicrob. Agents Chemother.* **34**, 476– 478 (1990).
4. Murphy, E. C. & Frick, I. M. Gram-positive anaerobic cocci - commensals and opportunistic pathogens. *FEMS Microbiol. Rev.* **37**, 520–553 (2013).
5. Barreiro, B. *et al.* Risk factors for the development of Haemophilus influenzae pneumonia in hospitalized adults. *Eur. Respir. J.* **8**, 1543– 1547 (1995).
6. Haemophilus influenzae: Causes and Transmission | CDC. https://[www.cdc.gov/hi-disease/about/causes-transmission.html.](http://www.cdc.gov/hi-disease/about/causes-transmission.html)
7. Hegstad, K. *et al.* Role of Horizontal Gene Transfer in the Development of Multidrug Resistance in Haemophilus influenzae. *mSphere* **5**, (2020).
8. Stuy, J. H. Chromosomally integrated conjugative plasmids are common in antibiotic-resistant Haemophilus influenzae. *J. Bacteriol.* **142**,

925–930 (1980).

1. Podschun, R. & Ullmann, U. Klebsiella spp. as nosocomial pathogens: Epidemiology, taxonomy, typing methods, and pathogenicity factors. *Clin. Microbiol. Rev.* **11**, 589–603 (1998).
2. Evans, D. R. *et al.* Systematic detection of horizontal gene transfer across genera among multidrug-resistant bacteria in a single hospital.

*Elife* **9**, 1–20 (2020).

1. Yigit, H. *et al.* Carbapenem-Resistant Strain of Klebsiella oxytoca Harboring Carbapenem-Hydrolyzing β-Lactamase KPC-2. *Antimicrob. Agents Chemother.* **47**, 3881–3889 (2003).
2. Klebsiella pneumoniae in Healthcare Settings | HAI | CDC. https://[www.cdc.gov/hai/organisms/klebsiella/klebsiella.html.](http://www.cdc.gov/hai/organisms/klebsiella/klebsiella.html)
3. Dixon, R. A. & Postgate, J. R. Transfer of nitrogen-fixation genes by conjugation in Klebsiella pneumoniae [12]. *Nature* vol. 234 47–48 (1971).
4. Samanta, I. & Bandyopadhyay, S. Klebsiella. in *Antimicrobial Resistance in Agriculture* 153–169 (Academic Press, 2020). doi:10.1016/b978- 0-12-815770-1.00014-6.
5. Pathogen Safety Data Sheets: Infectious Substances – Klebsiella spp. - Canada.ca. https://[www.canada.ca/en/public-](http://www.canada.ca/en/public-) health/services/laboratory-biosafety-biosecurity/pathogen-safety-data-sheets-risk-assessment/klebsiella.html.
6. Legionnaires Disease Cause and Spread | CDC. [https://w](http://www.cdc.gov/legionella/about/causes-transmission.html)ww.cdc.g[ov/legionella/about/causes-transmission.html.](http://www.cdc.gov/legionella/about/causes-transmission.html)
7. Gomez-Valero, L. *et al.* Extensive recombination events and horizontal gene transfer shaped the Legionella pneumophila genomes. *BMC Genomics* **12**, 536 (2011).
8. Infection | Leptospirosis | CDC. http[s://w](http://www.cdc.gov/leptospirosis/infection/index.html)ww.c[dc.gov/leptospirosis/infection/index.html.](http://www.cdc.gov/leptospirosis/infection/index.html)
9. Haake, D. A. *et al.* Molecular Evolution and Mosaicism of Leptospiral Outer Membrane Proteins Involves Horizontal DNA Transfer. *J. Bacteriol.* **186**, 2818–2828 (2004).
10. Picardeau, M. Conjugative transfer between Escherichia coli and Leptospira spp. as a new genetic tool. *Appl. Environ. Microbiol.* **74**, 319– 322 (2008).
11. Information for Health Professionals and Laboratories | Listeria | CDC. [https://w](http://www.cdc.gov/listeria/technical.html)ww.cdc.g[ov/listeria/technical.html.](http://www.cdc.gov/listeria/technical.html)
12. Orsi, R. H., Bakker, H. C. de. & Wiedmann, M. Listeria monocytogenes lineages: Genomics, evolution, ecology, and phenotypic characteristics. *Int. J. Med. Microbiol.* **301**, 79–96 (2011).
13. Kelly, B. G., Vespermann, A. & Bolton, D. J. Horizontal gene transfer of virulence determinants in selected bacterial foodborne pathogens.

*Food Chem. Toxicol.* **47**, 969–977 (2009).

1. Murphy, T. F. & Parameswaran, G. I. Moraxella catarrhalis, a human respiratory tract pathogen. *Clin. Infect. Dis.* **49**, 124–131 (2009).
2. Bootsma, H. J., Van Dijk, H., Vauterin, P., Verhoef, J. & Mooi, F. R. Genesis of BRO β-lactamase-producing Moraxella catarrhalis: Evidence for transformation-mediated horizontal transfer. *Mol. Microbiol.* **36**, 93–104 (2000).
3. Hays, J. Mobile Genetic Elements in Moraxella catarrhalis . *Mob. Genet. Elements* **1**, 155–158 (2011).
4. Wallace, R. J. *et al.* BRO β-lactamases of Branhamella catarrhalis and Moraxella subgenus moraxella, including evidence for chromosomal β-lactamase transfer by conjugation in B. catarrhalis, M. nonliquefaciens, and M. lacunata. *Antimicrob. Agents Chemother.* **33**, 1845–1854 (1989).
5. Mycoplasma pneumoniae Causes and Transmission | CDC. https://[www.cdc.gov/pneumonia/atypical/mycoplasma/about/causes-](http://www.cdc.gov/pneumonia/atypical/mycoplasma/about/causes-) transmission.html.
6. Xiao, L. *et al.* Comparative genome analysis of Mycoplasma pneumoniae. *BMC Genomics* **16**, 1–16 (2015).
7. Limeres Posse, J., Diz Dios, P. & Scully, C. Systemic Bacteria Transmissible by Kissing. *Saliva Prot. Transm. Dis.* 29–51 (2017) doi:10.1016/b978-0-12-813681-2.00003-2.
8. STD Facts - Gonorrhea. https://[www.cdc.gov/std/gonorrhea/stdfact-gonorrhea.htm.](http://www.cdc.gov/std/gonorrhea/stdfact-gonorrhea.htm)
9. Cehovin, A. & Lewis, S. B. Mobile genetic elements in Neisseria gonorrhoeae: Movement for change. *Pathog. Dis.* **75**, 1–12 (2017).
10. Pachulec, E. & van der Does, C. Conjugative plasmids of Neisseria gonorrhoeae. *PLoS One* **5**, (2010).
11. Meningococcal Disease (Neisseria meningitidis) | Disease Directory | Travelers’ Health | CDC. https://wwwnc.cdc.gov/travel/diseases/meningococcal-disease.
12. Brett, M. S. Y. Conjugal transfer of gonococcal βlactamase and conjugative plasmids to neisseria meningitidis. *J. Antimicrob. Chemother.*

**24**, 875–879 (1989).

1. Roberts, M. C. & Knapp, J. S. Transfer of β-lactamase plasmids from Neisseria gonorrhoeae to Neisseria meningitidis and commensal Neisseria species by the 25.2-megadalton conjugative plasmid. *Antimicrob. Agents Chemother.* **32**, 1430–1432 (1988).
2. Transmission | Nocardiosis | CDC. [https://w](http://www.cdc.gov/nocardiosis/transmission/index.html)ww.cdc.g[ov/nocardiosis/transmission/index.html.](http://www.cdc.gov/nocardiosis/transmission/index.html)
3. Pathogen Safety Data Sheets: Infectious Substances – Nocardia spp. - Canada.ca. https://[www.canada.ca/en/public-](http://www.canada.ca/en/public-)

health/services/laboratory-biosafety-biosecurity/pathogen-safety-data-sheets-risk-assessment/nocardia.html.

1. Jung, C. M., Crocker, F. H., Eberly, J. O. & Indest, K. J. Horizontal gene transfer (HGT) as a mechanism of disseminating RDX-degrading activity among Actinomycete bacteria. *J. Appl. Microbiol.* **110**, 1449–1459 (2011).
2. Salmonella (non-typhoidal). [https://w](http://www.who.int/news-room/fact-sheets/detail/salmonella-%28non-typhoidal%29)ww.who.i[nt/news-room/fact-sheets/detail/salmonella-%28non-typhoidal%29.](http://www.who.int/news-room/fact-sheets/detail/salmonella-%28non-typhoidal%29)
3. McMillan, E. A., Jackson, C. R. & Frye, J. G. Transferable Plasmids of Salmonella enterica Associated With Antibiotic Resistance Genes.

*Front. Microbiol.* **11**, (2020).

1. Rychlik, I., Gregorova, D. & Hradecka, H. Distribution and function of plasmids in Salmonella enterica. *Vet. Microbiol.* **112**, 1–10 (2006).
2. Mollerup, S. *et al.* Propionibacterium acnes: Disease-causing agent or common contaminant? detection in diverse patient samples by next- generation sequencing. *J. Clin. Microbiol.* **54**, 980–987 (2016).
3. Aoki, S., Nakase, K., Hayashi, N. & Noguchi, N. Transconjugation of erm(X) conferring high-level resistance of clindamycin for cutibacterium acnes. *J. Med. Microbiol.* **68**, 26–30 (2019).
4. Davidsson, S. *et al.* Prevalence of Flp Pili-encoding plasmids in Cutibacterium acnes isolates obtained from prostatic tissue. *Front. Microbiol.* **8**, 1–13 (2017).
5. Chen, C. Y. *et al.* Proteus mirabilis urinary tract infection and bacteremia: Risk factors, clinical presentation, and outcomes. *J. Microbiol. Immunol. Infect.* **45**, 228–236 (2012).
6. Harada, S., Ishii, Y., Saga, T., Tateda, K. & Yamaguchi, K. Chromosomally encoded blaCMY-2 located on a novel SXT/R391-related integrating conjugative element in a Proteus mirabilis clinical isolate. *Antimicrob. Agents Chemother.* **54**, 3545–3550 (2010).
7. Armbruster, C. E. & Mobley, H. L. T. Merging Mythology and Morphology: the multifaceted lifestyle of Proteus mirabilis. *Nat Rev Microbiol* **30**, 186–194 (2013).
8. Pathogen Safety Data Sheets: Infectious Substances – Proteus spp. - Canada.ca. https://[www.canada.ca/en/public-](http://www.canada.ca/en/public-) health/services/laboratory-biosafety-biosecurity/pathogen-safety-data-sheets-risk-assessment/proteus.html.
9. Girlich, D., Bonnin, R. A., Dortet, L. & Naas, T. Genetics of Acquired Antibiotic Resistance Genes in Proteus spp. *Front. Microbiol.* **11**, 1–21 (2020).
10. Li, X. *et al.* SXT/R391 integrative and conjugative elements in Proteus species reveal abundant genetic diversity and multidrug resistance.

*Sci. Rep.* **6**, 4–12 (2016).

1. Providencia species - Infectious Disease and Antimicrobial Agents. [http://antimicrobe.org/b227.asp.](http://antimicrobe.org/b227.asp)
2. Wie, S. H. Clinical significance of providencia bacteremia or bacteriuria. *Korean J. Intern. Med.* **30**, 167–169 (2015).
3. Mahrouki, S. *et al.* Nosocomial dissemination of plasmids carrying blaTEM-24, blaDHA-1, aac(6’)-Ib-cr, and qnrA6 in Providencia spp. strains isolated from a Tunisian hospital. *Diagn. Microbiol. Infect. Dis.* **81**, 50–52 (2015).
4. Olumuyiwa Olaitan, A., Diene, S. M., Victor Assous, M. & Rolain, J. M. Genomic plasticity of multidrug-resistant NDM-1 positive clinical isolate of providencia rettgeri. *Genome Biol. Evol.* **8**, 723–728 (2016).
5. Pseudomonas aeruginosa Infection | HAI | CDC. [https://w](http://www.cdc.gov/hai/organisms/pseudomonas.html)ww.cdc.g[ov/hai/organisms/pseudomonas.html.](http://www.cdc.gov/hai/organisms/pseudomonas.html)
6. Botelho, J., Grosso, F. & Peixe, L. Antibiotic resistance in Pseudomonas aeruginosa – Mechanisms, epidemiology and evolution. *Drug Resist. Updat.* **44**, 100640 (2019).
7. Zeng, L. *et al.* Genetic characterization of a blaVIM-24-Carrying IncP-7ß plasmid p1160-VIM and a blaVIM-4-harboring integrative and conjugative element Tn6413 from clinical pseudomonas aeruginosa. *Front. Microbiol.* **10**, 1–9 (2019).
8. Rickettsial Diseases (Including Spotted Fever & Typhus Fever Rickettsioses, Scrub Typhus, Anaplasmosis, and Ehrlichioses) - Chapter 4 - 2020 Yellow Book | Travelers’ Health | CDC. https://wwwnc.cdc.gov/travel/yellowbook/2020/travel-related-infectious- diseases/rickettsial-including-spotted-fever-and-typhus-fever-rickettsioses-scrub-typhus-anaplasmosis-and-ehr.
9. Merhej, V. & Raoult, D. Rickettsial evolution in the light of comparative genomics. *Biol. Rev.* **86**, 379–405 (2011).
10. Questions and Answers | Typhoid Fever | CDC. https://[www.cdc.gov/typhoid-fever/sources.html.](http://www.cdc.gov/typhoid-fever/sources.html)
11. Seth-Smith, H. M. B. *et al.* Structure, diversity, and mobility of the salmonella pathogenicity island 7 family of integrative and conjugative elements within enterobacteriaceae. *J. Bacteriol.* **194**, 1494–1504 (2012).
12. Buckle, J. & Buckle, J. Chapter 7 – Infection. *Clin. Aromather.* 130–167 (2015).
13. Nazzaro, G. Serratia marcescens. *Etymologia* **1**, 41–57 (2019).
14. Partridge, S. R., Kwong, S. M., Firth, N. & Jensen, S. O. Mobile genetic elements associated with antimicrobial resistance. *Clin. Microbiol. Rev.* **31**, 1–61 (2018).
15. Gruber, T. M. *et al.* Pathogenicity of pan-drug-resistant Serratia marcescens harbouring blaNDM-1. *J. Antimicrob. Chemother.* **70**, 1026– 1030 (2014).
16. Questions & Answers | Shigella – Shigellosis | CDC. [https://w](http://www.cdc.gov/shigella/general-information.html)ww.cdc.g[ov/shigella/general-information.html.](http://www.cdc.gov/shigella/general-information.html)
17. J, I., D, S., A, S., PD, C. & A, D. Characterization of antimicrobial resistance, plasmids, and gene cassettes in Shigella spp. from patients in

vietnam. *Microb. Drug Resist.* **9 Suppl 1**, (2003).

1. Staphylococcus aureus in Healthcare Settings | HAI | CDC. [https://w](http://www.cdc.gov/hai/organisms/staph.html)ww.cdc.g[ov/hai/organisms/staph.html.](http://www.cdc.gov/hai/organisms/staph.html)
2. Moskowitz, S. M. & Wiener-Kronish, J. P. Mechanisms of bacterial virulence in pulmonary infections. *Curr. Opin. Crit. Care* **16**, 8–12 (2010).
3. Denis, O. Route of transmission of Staphylococcus aureus. *Lancet Infect. Dis.* **17**, 124–125 (2017).
4. M, O. Staphylococcus epidermidis--the ‘accidental’ pathogen. *Nat. Rev. Microbiol.* **7**, 555–567 (2009).
5. Cafini, F. *et al.* Horizontal gene transmission of the cfr gene to MRSA and Enterococcus: Role of Staphylococcus epidermidis as a reservoir and alternative pathway for the spread of linezolid resistance. *J. Antimicrob. Chemother.* **71**, 587–592 (2016).
6. Sellner, J., Täuber, M. G. & Leib, S. L. Pathogenesis and pathophysiology of bacterial CNS infections. *Handb. Clin. Neurol.* **96**, 1–16 (2010).
7. Brochet, M. *et al.* Shaping a bacterial genome by large chromosomal replacements, the evolutionary history of Streptococcus agalactiae.

*Proc. Natl. Acad. Sci. U. S. A.* **105**, 15961–15966 (2008).

1. Clinical Information about Group B Strep | CDC. https://[www.cdc.gov/groupbstrep/clinicians/index.html.](http://www.cdc.gov/groupbstrep/clinicians/index.html)
2. Pinkbook: Pneumococcal Disease | CDC. https://[www.cdc.gov/vaccines/pubs/pinkbook/pneumo.html.](http://www.cdc.gov/vaccines/pubs/pinkbook/pneumo.html)
3. Lehtinen, S. *et al.* Horizontal gene transfer rate is not the primary determinant of observed antibiotic resistance frequencies in streptococcus pneumonia. *Sci. Adv.* **6**, 1–9 (2020).
4. Pharyngitis (Strep Throat): Information For Clinicians | CDC. https://[www.cdc.gov/groupastrep/diseases-hcp/strep-throat.html.](http://www.cdc.gov/groupastrep/diseases-hcp/strep-throat.html)
5. Del Grosso, M. *et al.* ICESpy009, a conjugative genetic element carrying mef(E) in Streptococcus pyogenes. *Antimicrob. Agents Chemother.* **60**, 3906–3912 (2016).
6. Haslam, D. B. & St. Geme, J. W. Viridans Streptococci, Abiotrophia and Granulicatella Species, and Streptococcus bovis. in *Principles and Practice of Pediatric Infectious Disease* (eds. Long, S. S., Pickering, L. K. & Prober, C. G.) 719–723 (Elsevier Saunders, 2008). doi:10.1016/b978-0-7020-3468-8.50127-9.
7. Doern, C. D. & Burnham, C. A. D. It’s not easy being green: The viridans group streptococci, with a focus on pediatric clinical manifestations. *J. Clin. Microbiol.* **48**, 3829–3835 (2010).
8. Balsalobre, L., Ferrándiz, M. J., Liñares, J., Tubau, F. & De la Campa, A. G. Viridans group streptococci are donors in horizontal transfer of topoisomerase IV genes to Streptococcus pneumoniae. *Antimicrob. Agents Chemother.* **47**, 2072–2081 (2003).
9. STD Facts - Syphilis (Detailed). [https://w](http://www.cdc.gov/std/syphilis/stdfact-syphilis-detailed.htm)ww.cdc.g[ov/std/syphilis/stdfact-syphilis-detailed.htm.](http://www.cdc.gov/std/syphilis/stdfact-syphilis-detailed.htm)
10. Yaws. [https://w](http://www.who.int/news-room/fact-sheets/detail/yaws)ww.who.i[nt/news-room/fact-sheets/detail/yaws.](http://www.who.int/news-room/fact-sheets/detail/yaws)
11. Pathogen Safety Data Sheets: Infectious Substances – Ureaplasma urealyticum - Canada.ca. https://[www.canada.ca/en/public-](http://www.canada.ca/en/public-) health/services/laboratory-biosafety-biosecurity/pathogen-safety-data-sheets-risk-assessment/ureaplasma-urealyticum.html.
12. Waites, K. B., Katz, B. & Schelonka, R. L. Mycoplasmas and ureaplasmas as neonatal pathogens. *Clin. Microbiol. Rev.* **18**, 757–789 (2005).
13. General Information | Cholera | CDC. https://[www.cdc.gov/cholera/general/index.html.](http://www.cdc.gov/cholera/general/index.html)
14. Verma, J. *et al.* Genomic plasticity associated with antimicrobial resistance in Vibrio cholerae. *Proc. Natl. Acad. Sci. U. S. A.* **116**, 6226– 6231 (2019).
15. Nelson, J. D. & McCracken, G. H. The pediatric infectious disease journal(r) newsletter: march 2009. *Pediatr. Infect. Dis. J.* **28**, A5 (2009).
16. Ecology and Transmission | Plague | CDC. [https://w](http://www.cdc.gov/plague/transmission/index.html)ww.cdc.g[ov/plague/transmission/index.html.](http://www.cdc.gov/plague/transmission/index.html)
